# Asynchronous telerehabilitation in prehabilitation and postoperative recovery for colorectal cancer: A protocol for a randomized controlled trial

**DOI:** 10.1371/journal.pone.0333649

**Published:** 2026-04-30

**Authors:** José Manuel Burgos-Bragado, Carolina Jiménez-Sánchez, Natalia Brandín-de la Cruz, Beatriz Carpallo-Porcar, Lilian Le Roux-Ethève, Juan Luis Blas-Laina, Jorge Alamillo-Salas, Paola Gracia Gimeno, Sandra Calvo

**Affiliations:** 1 Department of Physical Therapy, Faculty of Health Sciences, Universidad San Jorge, Villanueva de Gállego, Zaragoza, Spain; 2 Hospital Royo Villanova, Zaragoza, Spain; 3 Department of Physiatry and Nursing, Faculty of Health Sciences, University of Zaragoza, Zaragoza, Spain; 4 IIS Aragón, Aragon Health Research Institute, Zaragoza, Spain; Sichuan University, CHINA

## Abstract

**Introduction:**

Colorectal cancer (CRC) is a leading global malignancy, and surgery is frequently followed by complications, functional decline, and reduced quality of life. Multimodal prehabilitation and rehabilitation can improve physical recovery and psychosocial outcomes, but uptake is often limited by logistical and mobility barriers. Asynchronous telerehabilitation offers a flexible, patient**-**centered, and scalable approach; however, its effectiveness across the perioperative CRC pathway has not been rigorously evaluated. This trial will evaluate a multimodal asynchronous program delivered in prehabilitation and postoperative phases, against a booklet-based usual-care approach reflecting the pre-existing perioperative pathway in the study setting before trial initiation.

**Methods:**

This single-blind, parallel-group randomized controlled trial will compare an asynchronous multimodal telerehabilitation program with a booklet-based usual-care program in adults scheduled for elective CRC resection. Fifty-six participants will be randomized 1:1 to the telerehabilitation group (HEFORA platform) or the usual-care control group. The intervention includes a 2-week prehabilitation phase and a 4-week postoperative rehabilitation phase. Assessments will be performed at five time points: baseline (pre-prehabilitation), post-prehabilitation (pre-surgery), post-surgery (pre-rehabilitation), post-rehabilitation, and 3-month follow-up.

**Outcomes:**

The primary outcome is functional capacity, measured by the Six-Minute Walk Test distance. Secondary outcomes include muscle strength, body composition, pulmonary function, physical activity, sleep quality, psychosocial variables, health-related quality of life, treatment expectancy, usability, satisfaction, and adherence. Analyses will follow the intention-to-treat principle using longitudinal models and sensitivity analyses for missing data and adherence. **Conclusions:** This study will provide evidence on the role of asynchronous telerehabilitation in perioperative colorectal cancer care. Positive results could inform clinical guidelines, promote wider adoption of digital rehabilitation strategies, and support a more accessible, patient-centered, and cost-effective approach to oncologic recovery.

**Trial registration:**

ClinicalTrials.gov identifier: NCT06593678.

## Introduction

Colorectal cancer (CRC) represents a major global health challenge, combining high incidence and mortality with persistent functional decline and impaired quality of life (QoL). The perioperative course is often complicated by preoperative deconditioning, postoperative morbidity, and prolonged recovery, which increase healthcare utilization and impose significant indirect costs on patients, families, and health systems [1–4]. Consequently, strategies targeting modifiable risk factors before and after surgery have become a priority to optimize outcomes and reduce costs [5–7].

CRC comprises malignant epithelial tumors of the colon and rectum, predominantly adenocarcinomas that develop through the adenoma–carcinoma sequence. This process results from the stepwise accumulation of genetic and epigenetic alterations, frequently involving APC, KRAS, and TP53 pathways, which drive uncontrolled proliferation, invasion, and metastasis [8]. Clinical manifestations vary with tumor site and stage: early disease is often asymptomatic, while advanced stages may present with rectal bleeding, changes in bowel habits, abdominal pain, iron-deficiency anemia, weight loss, obstruction, or perforation [9]. Because early CRC usually lacks specific symptoms, population-based screening is essential to enable timely detection and treatment. Stool-based tests and colonoscopy have substantially improved prognosis by facilitating early-stage diagnosis [10].

The World Health Organization (WHO) reports that CRC is the third most frequently diagnosed cancer and the second leading cause of cancer-related death worldwide, accounting for nearly 10% of all cases and 9.6% of annual cancer deaths [11]. More than 1.9 million new cases and over 903,000 deaths occur globally each year [12]. In high-income countries, screening programs have shifted diagnosis toward earlier stages, but demographic ageing and lifestyle factors sustain the burden. In low- and middle-income countries, incidence continues to rise, driven by dietary westernization and reduced physical activity [13]. The risk factors for CRC reflect the interplay of modifiable and non-modifiable determinants. Smoking, harmful alcohol consumption, excess adiposity, sedentary behavior, low dietary fiber intake, and high consumption of red and processed meats are consistently associated with increased risk [10,11]. Age, family history, and hereditary syndromes such as Lynch syndrome and familial adenomatous polyposis [14], represent key non-modifiable risks that are critical for guiding personalized prevention strategies and surveillance protocols [14,15].

The management of CRC requires a comprehensive, multidisciplinary approach integrating surgery, chemotherapy, radiotherapy and, when indicated, immunotherapy. Current guidelines emphasize multidisciplinary team (MDT) involvement, especially through tumor board conferences, to optimize individualized treatment decisions, which rely on tumor stage, molecular profiling, and patient characteristics [16]. Surgical resection, often accompanied by lymphadenectomy, remains the cornerstone of curative treatment, while total mesorectal excision (TME) is the standard surgical technique for rectal cancer, enhancing oncologic control and sphincter preservation [17]. However, postoperative recovery is frequently hindered by pain, fatigue, sarcopenia, and psychological distress, particularly among frail and older adults. These complications contribute to functional decline, reduced autonomy, delayed return to daily activities, and compromised adherence to adjuvant therapies [18,19].

Despite advances in surgical and oncologic therapies, CRC surgery remains associated with significant morbidity and long-term functional deterioration [20]. Structured rehabilitation programs, initiated both preoperatively (prehabilitation) and postoperatively, have been shown to enhance functional recovery, reduce complication rates, shorten hospital stays, and improve QoL [21–23]. Multimodal prehabilitation strategies, combining structured physical exercise, nutritional optimization and therapeutic education, have demonstrated improvements in functional capacity, accelerated independence, and reduced healthcare costs. Similarly, postoperative rehabilitation integrated into Enhanced Recovery After Surgery (ERAS) protocols has proven effective in restoring muscle strength, mobility, and overall well-being [24–26].

Conventional rehabilitation programs, while effective, often face barriers such as travel distance, scheduling conflicts and mobility limitations, leading to reduced access and adherence. Telerehabilitation, defined as the remote delivery of rehabilitation services via digital technologies, offers a flexible, scalable, and patient-centered alternative [27]. By providing exercise guidance, education, and monitoring through digital platforms, telerehabilitation supports continuity of care while reducing logistical burdens. Evidence from oncology and other fields indicates that this modality may improve adherence, functional outcomes and quality of life, though rigorous trials in perioperative CRC care remain scarce [27–31]. Moreover, telerehabilitation has shown promise in cardiovascular, neurological and musculoskeletal conditions [29–31], demonstrating improvements in functional capacity, cardiorespiratory fitness, cognitive functioning, pain, fatigue and QoL, along with high feasibility and acceptability [28,29].

Most studies in oncology have focused on synchronous telerehabilitation or have lacked structured follow-up, with limited evidence on psychosocial outcomes or adherence. Importantly, no randomized controlled trial has yet evaluated the effectiveness of an asynchronous multimodal telerehabilitation program spanning both prehabilitation and postoperative phases in CRC patients [30,31].

Therefore, this protocol aims to evaluate the effectiveness of a structured asynchronous telerehabilitation program delivered across the prehabilitation and postoperative rehabilitation phases on physical, psychosocial, and health-related outcomes in patients undergoing CRC surgery, compared with a booklet-based usual-care comparator reflecting the pre-existing perioperative pathway available in the study setting before trial initiation. The study is intended to generate novel evidence on the role of digital rehabilitation strategies in enhancing recovery, promoting autonomy, and improving longer-term outcomes in oncologic surgery.

## Methods

### Study design

This study is designed as a single-blind, parallel-group randomized controlled trial (RCT) to evaluate the effectiveness of an asynchronous telerehabilitation program integrated into the prehabilitation and postoperative rehabilitation pathway for patients undergoing elective CRC surgery. The protocol follows the Standard Protocol Items: Recommendations for Interventional Trials (SPIRIT) guidelines and is informed by the Consolidated Standards of Reporting Trials (CONSORT) 2010 recommendations for reporting randomized trials.

**Fig 1:**
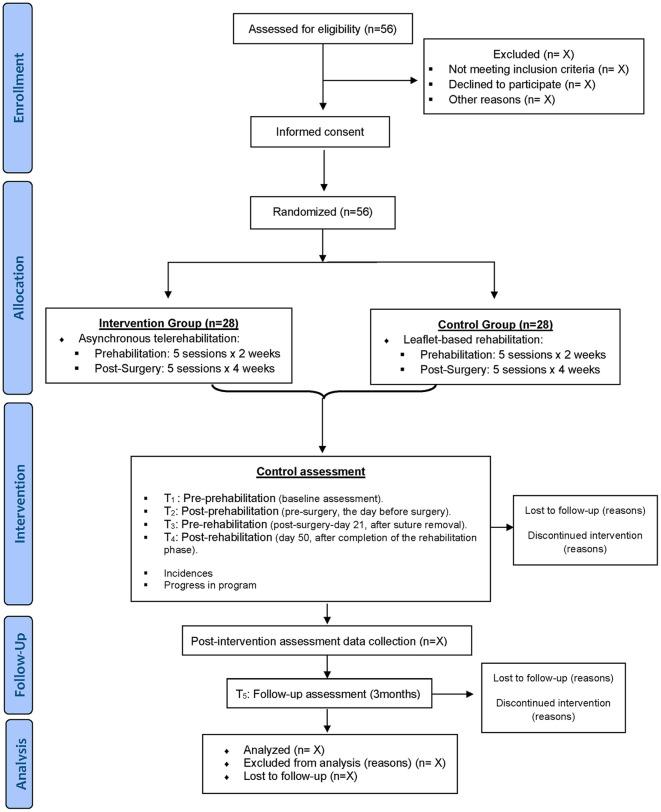
Study diagram.

Participants will be randomly allocated to one of two groups: an intervention group (IG), which will receive an asynchronous multimodal telerehabilitation program, and a usual-care control group (CG), which will receive the same multimodal physiotherapy content through an educational booklet reflecting the perioperative pathway available in the study setting prior to study initiation. The study period will comprise 18 weeks: a 2-week prehabilitation phase, a 4-week postoperative rehabilitation phase, and a follow-up assessment conducted 12 weeks after completion of the intervention (Fig 1). Recruitment for this randomized controlled trial began on August 30, 2024. Participants are currently actively enrolled in the study, with recruitment expected to be completed by the end of 2025. Data collection is anticipated to be completed by April 2026, and the study results are expected to be available by July 2026.

This trial protocol has been reviewed and approved by the Ethics Committee of Aragón (Reference: PI23/557) and prospectively registered in ClinicalTrials.gov (NCT06593678.).

### Setting and population

The study will be conducted in the Department of General and Digestive Surgery of the Royo Villanova Hospital (Zaragoza, Spain), in collaboration with the hospital’s Departments of Endocrinology and Oncology to ensure a multidisciplinary approach. Patient recruitment will be carried out under the direction of the Head of the Department of General and Digestive Surgery, who will identify eligible candidates during preoperative consultations. Participants will be adult patients diagnosed with CRC, scheduled for elective surgical resection, and meeting the predefined eligibility criteria.

### Eligibility criteria

All potential participants will be informed about the study and must provide written consent before participating. The inclusion criteria will be as follows: 1) Adults aged 18–80 years; 2) Patients scheduled for elective CRC surgery at Hospital Royo Villanova; 3) Initial consultation at the Department of General and Digestive Surgery; 4) Functionally independent individuals able to perform walking and pulmonary function tests; 5) Preoperative classification of I, II or III according to the American Society of Anesthesiologists (ASA) classification and 6) Willingness to participate in the study and signed informed consent.

The exclusion criteria will be: 1) Patients over 80 years old; 2) Preoperative ASA classification IV; 3) Musculoskeletal, inflammatory or other pathological conditions preventing physical exercise; 4) Central and/or peripheral neurological disorders limiting participation in the rehabilitation program; 5) Unstable concomitant cardiac conditions, including cardiac arrhythmias, hypertension, angina or other conditions contraindicating moderate-intensity exercise; 6) Psychiatric disorders diagnosed by a psychiatrist; 7) Lack of access to an internet-enabled mobile device or computer at home; and 8) Refusal to participate or lack of a signed consent form.

### Allocation and blinding

Participants will be randomly assigned in a 1:1 ratio to either the IG or CG using computer-generated block randomization (www.randomizer.org), stratified by age and sex. Allocation will be concealed using sequentially numbered, opaque, sealed envelopes handled by an independent researcher not involved in the intervention or outcome assessment.

All participants will be assigned a unique identification code to ensure confidentiality. The outcome assessor will be blinded to the group allocation, and the researcher conducting the intervention will not be involved in any assessment procedures, preserving single-blind conditions.

### Procedure

The recruitment process will be carried out in the Department of General and Digestive Surgery of the Royo Villanova Hospital (Zaragoza, Spain) under the coordination of the Head of Department. During the first preoperative consultation, eligible candidates will receive a comprehensive information sheet detailing the study objectives, procedures, potential risks and benefits, and contact details of the principal investigator for further questions.

Patients who express an interest in participating will be screened by a trained researcher to ensure that they meet the inclusion and exclusion criteria. Once enrolled, participants will sign the informed consent form and complete a baseline evaluation (T1: pre-prehabilitation) conducted by a physiotherapist blinded to group allocation. This assessment will include standardized questionnaires and physical performance tests. Questionnaires will be administered in a standardized manner in both groups, and the physiotherapist conducting the assessment will be available only to clarify item wording and ensure consistent understanding, without influencing participants’ responses. Each session will last approximately 45 minutes and will be performed in the hospital under standardized conditions to enhance the measurement reliability and minimize external variability.

After the initial assessment, participants will be randomized into one of two study groups: (1) the IG, which will receive the structured asynchronous telerehabilitation program; or (2) the CG, which will receive the same multimodal physiotherapy content through a booklet-based usual-care program.

Participants allocated to the IG will receive access to the HEFORA platform, an asynchronous digital tool designed for therapeutic exercise prescription and education. A physiotherapist will assist each participant with installation and navigation on a personal mobile phone, tablet, or computer, and will provide instructions on how to access the exercise videos, complete session logs, review progress, and communicate with the clinical team. Participants in the CG will receive a printed booklet containing equivalent educational content, exercise instructions, and progression guidelines for home-based completion of the program. They will also receive an initial face-to-face explanation from the treating physiotherapist regarding exercise performance, progression across phases, and completion of the paper adherence diary. In our setting, this booklet-based pathway represented usual perioperative care before the trial, as patients were not routinely referred to the Physiotherapy Service because no structured referral pathway and insufficient resources were available within the hospital.

**Fig 2:**
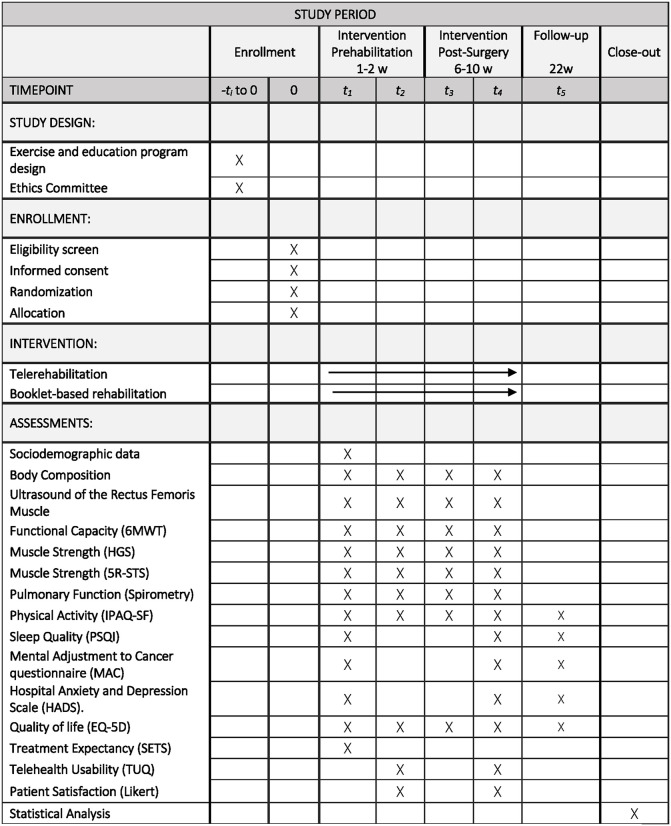
Schedule of enrollment, interventions, and assessments.

A schedule of enrollment, interventions, and assessments is outlined in Fig 2.

All participants will be instructed to maintain a daily exercise log, either digital or paper-based, to document adherence. Adherence will be recorded using a structured adherence diary indicating whether each prescribed exercise session was completed fully, partially, with modifications, or not completed, together with post-session fatigue rated on a 0–10 numerical rating scale and the occurrence of any adverse effects. In the IG, exercise progression and safety will be monitored asynchronously through the HEFORA platform. In the CG, follow-up will be conducted through the booklet, routine clinical reviews, scheduled assessments, and verification of the paper-based adherence diary during those visits by a physiotherapist not involved in blinded outcome assessment. Reminders and adaptive exercise progression will also be provided in the CG, but only within the context of face-to-face clinical follow-up rather than through asynchronous digital monitoring.

Study outcomes will be evaluated at five key time points: Time 1 (T1): pre-prehabilitation (baseline assessment); Time 2 (T2): post-prehabilitation (pre-surgery, the day before surgery); Time 3 (T3): pre-rehabilitation (post-surgery-day 21, after suture removal); Time 4 (T4): post-rehabilitation (day 50, after completion of the rehabilitation phase) and Time 5 (T5): follow-up (three months after T4 by telephone assessment).

Assessments at T1, T2, T3, and T4 will be conducted in person by the same physiotherapist, who will remain blinded to group allocation throughout the study. All evaluations will follow a standardized protocol to ensure consistency across time points. Performance-based assessments will be administered using identical instructions and procedures across groups and time points in order to reduce the risk of differential assessment bias. The final follow-up (T5) will be conducted remotely using a structured telephone interview to assess long-term psychosocial well-being and health-related quality of life.

### Intervention

The intervention will consist of a six-week multimodal telerehabilitation program, structured into two phases: a two-week prehabilitation phase and a four-week postoperative rehabilitation phase. The program has been developed in accordance with international clinical guidelines for the perioperative rehabilitation in CRC patients [19,32].

The prehabilitation period will be limited to two weeks, reflecting the usual interval between diagnosis and elective CRC surgery in the study setting and the logistical constraints that govern surgical scheduling. During this phase, all participants will follow the institutional ERAS protocol [33]. This protocol includes preoperative education sessions, covering surgical procedures, the expected postoperative course, and self-care strategies; perioperative medical optimization, including medication adjustment and nutritional assessment; and a structured physical training component designed to improve aerobic capacity, muscular strength, and mobility before surgery. In addition, participants will be advised to follow a low-fiber diet during the eight days preceding surgery in order to reduce intestinal residue, improve intraoperative visibility, and decrease the risk of postoperative complications.

Within the ERAS framework, the physiotherapy team will deliver and monitor the rehabilitation intervention throughout both the prehabilitation and postoperative phases.

**Intervention Group (IG):** the participants allocated to the IG will access the program through HEFORA, an asynchronous telerehabilitation platform available via mobile application or web interface (HEFORA, Fisio Consultores, Zaragoza, Spain). Each exercise will be presented as a video demonstration with detailed written instructions. The program will be individually tailored according to each participant’s baseline functional assessment and structured into three progression levels (basic, intermediate, advanced) for each exercise category. Initial allocation to a level will be determined by baseline performance, comorbidities, and symptom profile. Progression will be re-evaluated weekly by the assigned physiotherapist, based on participants’ self-reported exertion, symptoms, and adherence data recorded on the HEFORA platform.

Exercise intensity will be guided using a modified Borg Rating of Perceived Exertion scale (target range 3–7) to ensure both safety and effectiveness. Within each progression level, training parameters (including sets, repetitions, tempo, and rest intervals) will be adjusted to promote gradual overload while respecting surgical timelines and recovery phases.

The physiotherapy program will include the following core components:

(1) **Therapeutic education**: participants will receive evidence-based information (via video or printed materials, depending on group allocation) on the principles of prehabilitation, self-care during the postoperative period, strategies for early mobilization, and healthy lifestyle recommendations to promote recovery and autonomy.
(2) **Respiratory exercises**: a daily regimen of breathing exercises will be prescribed, including diaphragmatic breathing, costal expansion techniques, controlled breathing patterns, and incentive spirometry. These exercises aim to improve pulmonary function and reduce the incidence of postoperative pulmonary complications.
(3) **Aerobic exercise**: participants will be encouraged to engage in moderate-to-vigorous aerobic activity for a total of 150–300 minutes per week, tailored to their physical condition and surgical timeline, with the goal of enhancing cardiovascular endurance and general fitness.
(4) **Strength training**: resistance exercises targeting major muscle groups will be performed at least four times per week. Typical movements will include bent-over rows, wall push-ups, arm curls, front and lateral shoulder raises with weights, squats (including wall squats), lunges, hip flexor activations, and gluteus maximus and medius strengthening. Training will progress in a structured manner, increasing from 2–3 sets of 8 repetitions to 3 sets of 15 repetitions as tolerated. The program will be updated weekly based on self-reported exertion, symptoms, and recovery status. Adjustments to exercise type, load, or frequency will be made by the physiotherapist responsible for the intervention.

Adherence in the IG will be digitally monitored via the HEFORA platform. Each exercise will include a “Done” button to confirm completion, and participants will be able to record partial completion, reasons for missed sessions, and post-session fatigue using a 0–10 scale. Asynchronous messaging between participants and physiotherapists will enable timely feedback, individualized progression, and the resolution of technical or clinical issues. Automated adherence reminders will be triggered if ≥48 hours elapse without a recorded session. Data from HEFORA will be exported as structured logs to support process evaluation and exploratory analyses.

**Control Group (CG):** Participants allocated to the CG will follow the same exercise and education protocol as those in the IG, structured according to the same three progression levels: basic, intermediate, and advanced. However, all intervention materials will be delivered in the form of a printed booklet containing, step-by-step written instructions and illustrative photographs for each exercise. The strength training component will be organized into three progressive phases, with specific exercises, sets, and repetitions prescribed on the basis of the baseline assessment and explained by the treating physiotherapist during the baseline visit. Progression across these phases will follow the pre-established booklet instructions and the participant’s clinical course. In the CG, follow-up will be conducted through the booklet, routine clinical reviews, scheduled evaluations, and verification of the paper-based adherence diary by the physiotherapist during those visits, without platform-based monitoring or asynchronous digital communication between visits. Reminders and adaptive progression will also be provided in the CG, but only within the context of face-to-face clinical follow-up rather than asynchronous digital monitoring. Accordingly, this group represents booklet-based usual perioperative care rather than an inactive comparator.

Adherence in the CG will be monitored using a standardized paper exercise diary incorporated into the booklet. Participants will be instructed to perform the prescribed exercises independently at home and to complete a daily log documenting whether each exercise was performed fully, partially, or not performed, together with perceived exertion and any adverse effects. These diaries will be reviewed by the medical and physiotherapy team during scheduled clinical follow-up visits at T2, T3, and T4, in order to support progression decisions and ensure accurate interpretation of adherence. Adherence records will also be verified at each assessment time point, and any discrepancies between self-reported adherence and clinical observations will be documented to inform sensitivity analyses.

### Outcome measures

All study variables will be assessed under standardized conditions at each evaluation point in order to minimize measurement variability.

#### Primary outcome.

The primary outcome of this trial will be the functional capacity, assessed using the Six-Minute Walk Test (6MWT). This submaximal exercise test will be administered following standardized procedures as established by the American Thoracic Society (ATS) and the European Respiratory Society (ERS) [34,35]. The 6MWT quantifies the maximum distance (in meters) that a person can walk in six minutes along a flat, 30-meter corridor, providing an objective measure of submaximal aerobic endurance, cardiorespiratory fitness, and overall physical function [35].

The 6MWT is considered a valid and reliable outcome measure in patients undergoing CRC surgery, as it reflects functional recovery trajectories and physical performance throughout the perioperative period [35,36]. By capturing the integrative physiological response of the cardiovascular, respiratory, and musculoskeletal systems, the test offers a comprehensive indicator of physical capacity and is sensitive to clinically meaningful changes over time [37,38].

#### Secondary outcomes.

Secondary outcomes are prespecified as either key supportive secondary outcomes, including muscle strength, body composition, pulmonary function, and health-related quality of life, or as exploratory and implementation-focused outcomes, including physical activity, sleep quality, psychosocial variables, treatment expectancy, usability, satisfaction, and adherence. This distinction is intended to enhance interpretability and to clarify that not all secondary endpoints are meant to support confirmatory inference to the same extent.

#### Body composition.

Body composition will be assessed using bioelectrical impedance analysis (BIA) with the Tanita BC-601 InnerScan®V (Japan) device. This validated, non-invasive method provides estimates of visceral fat percentage, total body weight, body fat percentage, skeletal muscle mass, and total body water [39,40]. BIA is widely used in both clinical and research settings because of its reproducibility, ease of use, and ability to track changes in body composition over time [39,41]. Because BIA-derived estimates may be influenced by hydration status and perioperative inflammatory changes, these measures will be interpreted cautiously and in conjunction with anthropometric variables and ultrasound assessment of the rectus femoris muscle rather than as stand-alone indicators of true changes in muscle or fat mass. Whenever feasible, BIA assessments will be scheduled at the same time of day and under consistent pre-assessment conditions across time points in order to reduce short-term measurement variability. These parameters nevertheless provide useful complementary information on metabolic status, body composition, and exercise tolerance in patients undergoing CRC treatment.

In addition to BIA, waist and hip circumference will be measured according to a standardized anthropometric protocol to assess central adiposity. These anthropometric indices are clinically relevant because they are strongly associated with metabolic dysfunction, systemic inflammation and cancer-related prognostic factors [42]. In the context of CRC rehabilitation, increased visceral adiposity and low muscle mass (i.e., sarcopenia) have been associated with poorer functional outcomes, higher rates of postoperative complications, and delayed recovery [43,44]. These anthropometric measures will therefore be interpreted alongside BIA and ultrasound findings to support a more comprehensive evaluation of perioperative body-composition changes.

#### Ultrasound assessment of the rectus femoris muscle.

Ultrasound imaging of the rectus femoris muscle will be used to assess skeletal muscle morphology and to monitor changes in muscle mass as a surrogate marker for nutritional status and sarcopenia. This non-invasive, bedside technique has shown high validity and reliability in both oncology and surgical populations and is widely used to detect early muscle wasting in patients undergoing cancer treatment or major surgery [45,46].

The assessments will be performed using a high-frequency (7–12 MHz) linear transducer, with the participant in a relaxed supine position. The transducer will be placed perpendicularly at the lower third (distal third) of the distance between the anterior superior iliac spine (ASIS) and the superior border of the patella on the dominant leg, following standardized anatomical landmarks to ensure reproducibility and accuracy across different time points [47].

The main parameters recorded will include muscle thickness, cross-sectional area (CSA) and echogenicity, the latter being analyzed using grayscale histogram analysis as a marker of muscle quality and intramuscular adiposity. This method allows the monitoring of musculoskeletal adaptations during the perioperative period and the response to therapeutic exercise and nutritional interventions [48].

In patients with CCR, reduced muscle thickness and increased echogenicity of the rectus femoris have been independently associated with higher postoperative complication rates, longer hospital stays, and impaired clinical outcomes [49]. Therefore, serial ultrasound assessments provide valuable complementary data to conventional anthropometric and functional measurements, supporting a comprehensive and dynamic evaluation of the patient status throughout the rehabilitation process.

#### Muscle strength.

Muscle strength will be assessed using two validated functional tests: the Handgrip Strength Test (HGS) for upper limb strength, and the Five-Repetition Sit-to-Stand Test (5R-STS) for lower limb function. Both measures are widely used in oncologic rehabilitation as indicators of neuromuscular performance, physical independence, and postoperative recovery potential [50,51].

Upper limb strength will be evaluated using a hydraulic hand-held dynamometer (Jamar® Hand Dynamometer, Sammons Preston Rolyan, Bolingbrook, IL, USA) following standardized testing procedures [52]. The participants will be seated in a shoulder neutral position, the elbow flexed at 90°, and the wrist in a neutral posture. Each participant will perform three maximal voluntary contractions with each hand, holding the grip for three seconds. The highest value (in kilograms) will be recorded for analysis. Handgrip strength is considered a robust surrogate marker for sarcopenia and nutritional status and is predictive of postoperative complications and overall prognosis in CRC patients [53].

Lower limb strength will be assessed using the 5R-STS, in which participants are instructed to stand up and return from a sitting position five times as quickly as possible, with their arms crossed over the chest to avoid upper limb compensation. The total time (in seconds) will be recorded using a standardized chair height (43–47 cm) to ensure consistency. This test reflects the functional mobility and the lower-extremity muscle power, and is strongly associated with fall risk, frailty, and recovery of autonomy after surgery [51,54].

#### Pulmonary function (spirometry).

Pulmonary function will be evaluated using standardized spirometry to assess changes in respiratory capacity associated with the telerehabilitation intervention. The two primary parameters to be measured will be: 1) Forced Vital Capacity (FVC): the maximum volume of air forcibly exhaled following a full inhalation and 2) Forced Expiratory Volume in 1 second (FEV₁): the volume of air exhaled during the first second of the forced expiratory maneuver [55].

The spirometry measurements will be conducted using a calibrated digital spirometer, following the technical standards established by the ERS and the ATS [56]. Each participant will perform a minimum of three acceptable and reproducible forced expiratory maneuvers while seated, and the highest consistent value will be retained for analysis. The results will be expressed both in absolute terms (liters) and as a percentage of the predicted values, adjusted for age, gender, height, and body weight [56].

In the perioperative management of CRC, a reduction in lung function is frequently observed, which is associated with a higher rate of postoperative pulmonary complications and delayed mobilization. Specifically, decreased preoperative FVC has been identified as an independent predictor of pulmonary complications such as pneumonia after colorectal cancer surgery [57]. Similarly, a large cohort study in high-risk abdominal surgery patients demonstrated that each 1% increase in preoperative FVC was associated with a 2% reduction in the risk of postoperative pulmonary complications [58]. Spirometry provides a clinically meaningful and objective indicator of respiratory performance and preoperative risk stratification [59].

In this study, spirometry assessments will be performed at several time points to compare the development of respiratory function between the groups. This will help to determine whether participation in the telerehabilitation program, particularly the structured respiratory training component, leads to better maintenance or improvement of lung capacity compared to standard leaflet-based rehabilitation.

#### Physical activity level (IPAQ-SF).

Self-reported level of physical activity will be assessed using the International Physical Activity Questionnaire – Short Form (IPAQ-SF). This validated tool is widely used in clinical and epidemiologic research to quantify habitual physical activity in adults and to monitor changes in response to health interventions [60,61].

The IPAQ-SF consists of seven items that measure the frequency and duration (minutes per day and days per week) of physical activity performed in the past seven days across three intensity domains: vigorous activity, moderate activity and walking, and time spent sitting. The answers will be used to calculate total physical activity in minutes per week (Metabolic Equivalent Task, MET-min/week) and allow categorization into low, moderate or high physical activity according to the IPAQ scoring protocol [60].

This questionnaire has shown acceptable reliability and validity for the assessment of physical activity levels in cancer survivors and perioperative patients [62,63]. In the context of CRC rehabilitation, physical activity monitoring is critical to understanding behavioral adaptations, adherence to exercise recommendations, and their potential associations with clinical recovery, functional outcomes, and QoL.

The IPAQ-SF will be administered at each of the five time points (T1–T5), allowing longitudinal analysis of changes in physical activity levels during the intervention and follow-up periods.

#### Sleep quality (pittsburgh sleep quality index – PSQI).

Sleep quality will be assessed using the Pittsburgh Sleep Quality Index (PSQI), a widely validated self-report questionnaire to measure sleep disturbances and habitual sleep patterns in the past month [64]. The PSQI is considered the gold standard for assessing sleep in clinical and oncology patients’ populations due to its reliability, multidimensional structure, and sensitivity to change [65,66].

The questionnaire consists of 19 items, which are divided into seven components: Subjective sleep quality, sleep latency, sleep duration, habitual sleep efficiency, sleep disturbances, use of sleep medication, daytime sleep disturbances.

Each component is scored on a scale from 0–3, resulting in a global score ranging from 0 to 21, with higher scores indicating poorer sleep quality. A total PSQI score of >5 is generally used as a cut-off point for clinically significant sleep disturbance [64].

In CRC patients, poor sleep quality has been associated with increased fatigue, mood disturbances, impaired immune function and lower health-related quality of life, particularly during the perioperative and adjuvant treatment phases [67].

Assessment of sleep patterns throughout the rehabilitation process will provide important insights into the psychosomatic impact of telerehabilitation and help to identify potential areas for supportive care interventions. The PSQI will be administered at all five evaluation time points (T1–T5) to monitor changes in sleep quality over time and compare results between groups.

#### Psychosocial factors.

Psychological response to cancer and emotional distress will be assessed using two validated instruments: the Mental Adjustment to Cancer questionnaire (MAC) and the Hospital Anxiety and Depression Scale (HADS).

The MAC questionnaire evaluates patients’ cognitive-behavioral coping styles in the context of cancer. It distinguishes between adaptive (positive adjustment) and maladaptive (negative adjustment) responses. Higher scores on the positive dimensions, such as fighting spirit and positive orientation to the illness, reflect active, goal-oriented coping strategies that have been associated with greater psychological resilience and improved treatment adherence [68,69]. Conversely, elevated scores in the negative domains, such as helpless–hopelessness, anxious preoccupation, or fatalism, indicate passive or avoidant coping mechanisms, which are typically associated with emotional distress, reduced QoL, and poorer clinical prognosis [70].

The HADS, a screening tool widely used in oncology for detecting clinically relevant symptoms of anxiety and depression, will be used in the study to assess emotional symptoms. The scale consists of 14 items, equally divided into two subscales: HADS-Anxiety (HADS-A) and HADS-Depression (HADS-D). Each item is rated from 0 to 3, yielding subscale scores ranging from 0 to 21. In clinical practice, the following cut-off thresholds are commonly used: 0–7 (normal), 8–10 (borderline or mild symptoms), and ≥11 (probable clinical case) [71,72].

In CRC populations, elevated HADS scores have been consistently associated with increased psychological burden, diminished QoL, and reduced engagement with treatment regimens. Therefore, the interpretation of MAC and HADS scores will be placed in the context of each patient’s overall clinical profile. In the case of high-risk scores, a comprehensive psychosocial evaluation and referral to mental health or psycho-oncology services when indicated [70].

#### Quality of life EuroQol 5D.

Health-related quality of life (HRQoL) will be assessed using the EuroQol-5D (EQ-5D) questionnaire, a validated and widely used self-administered instrument to measure general health status and patient-reported outcomes [73,74]. The EQ-5D captures key domains of physical and psychosocial functioning and is particularly valuable for monitoring changes in health-related quality of life during cancer treatment and rehabilitation [75,76].

The instrument comprises two components: 1) The EQ-5D descriptive system, which evaluates five health dimensions: mobility, self-care, usual activities, pain/discomfort, and anxiety/depression. Each dimension is rated on five levels in the EQ-5D-5L version (ranging from “no problems” to “extreme problems”). The combination of these ratings produces a five-digit health profile, which can be converted into a utility index (EQ-5D index) using a country-specific assessment algorithm. This index ranges from 0.000 (equivalent to death) to 1.000 (full health) and may also include negative values, representing health states perceived as worse than death [74]. 2) The EQ Visual Analogue Scale (EQ-VAS), which captures the participant’s self-perceived overall health status. Participants indicate their current state of health on a vertical scale ranging from 0 (worst imaginable health state) to 100 (best imaginable health state) [73].

In the context of oncological rehabilitation, especially for patients undergoing CRC surgery, the EQ-5D is a widely used tool to assess the treatment burden, the course of recovery and the impact of supportive interventions on physical, emotional and social well-being [75,77,78].

#### Treatment expectancy (Stanford Expectations of Treatment Scale – SETS).

Treatment expectancy will be assessed using the Stanford Expectations of Treatment Scale (SETS), a validated self-report instrument designed to measure patients’ beliefs and expectations regarding the effectiveness and potential benefits of a therapeutic intervention [79]. This construct has been shown to influence treatment adherence, engagement, and clinical outcomes, particularly in the context of behavioral and rehabilitative interventions [80].

The SETS consists of 6 items, each of which is rated on a 7-point Likert scale ranging from 1 (“strongly disagree”) to 7 (“strongly agree”). The scale captures two dimensions of expectancy: (1) outcome expectancy, defined as the belief that the treatment will be beneficial, and (2) process expectancy, defined as the belief that the treatment will be delivered competently and professionally [79].

The item scores are summed to produce a global expectancy score, with higher scores indicating more positive treatment expectations. The SETS has demonstrated good psychometric properties, including internal consistency and predictive validity, in populations undergoing various physical and psychological interventions [79,80].

In the context of prehabilitation and postoperative rehabilitation for CCR, patient expectations may play a critical role in motivation, engagement with home-based programs, and perceived benefit of telerehabilitation. By including the SETS, this study will explore the potential moderating effect of expectancy on treatment response [79].

The SETS will be administered at baseline (T1) to capture initial expectations before the intervention and at post-intervention (T4) to investigate possible changes in perception over time.

#### Telehealth usability (Telehealth Usability Questionnaire – TUQ).

The usability and user experience of the telerehabilitation platform will be assessed using the Telehealth Usability Questionnaire (TUQ), a validated instrument specifically designed to evaluate patients’ perceptions of telehealth systems across multiple domains [81]. The TUQ provides a comprehensive assessment of the practicality, satisfaction and perceived value of remote healthcare delivery tools, and has been validated in a variety of clinical and technological contexts, including applications in cancer rehabilitation and chronic disease management [82,83].

The TUQ consists of 21 items, each of which is rated on a 7-point Likert scale (1 = strongly disagree to 7 = strongly agree), covering six core dimensions: Usefulness, ease of use and learnability, interface quality, interaction quality, reliability, satisfaction and future use [81].

A total and subscale score will be calculated from the item responses, with higher values indicating better usability and user satisfaction. The questionnaire will be administered exclusively to the participants of the IG (telerehabilitation) at the end of the postoperative rehabilitation phase (T4), to evaluate their experience with the HEFORA platform.

Understanding the usability of digital health tools is essential for interpreting adherence and engagement in the telerehabilitation context. The TUQ will provide key insights into the acceptability, accessibility, and perceived value of the asynchronous platform used in this study, which can serve as a basis for future implementation and scalability strategies.

#### Patient satisfaction.

Patient satisfaction with the rehabilitation program will be evaluated using an ad hoc satisfaction survey. This approach has been widely employed in oncology rehabilitation and digital health studies, as patient-reported satisfaction is a key determinant of intervention feasibility, adherence, and long-term sustainability [84,85]. The instrument aims to assess the participants’ subjective perception of the intervention received, whether through the telerehabilitation platform or the standard leaflet-based approach, focusing on the quality of the content, the delivery modality and the overall user experience.

The survey includes two core questions that apply to all participants, which evaluate: 1) Satisfaction with the overall exercise program and 2) Satisfaction with the attention and support received during the intervention.

In addition, participants in the IG will rate: 1) The HEFORA platform (accessibility and ease of use); 2) The exercise video materials; 3) The clarity of exercise instructions and 4) The educational video content. Participants in the CG will provide feedback on the educational leaflet.

Each item will be scored on a 5-point Likert scale ranging from 0 (“very dissatisfied”) to 4 (“very satisfied”). The Satisfaction Survey will be administered at two time points: T2 (post-prehabilitation): To assess early impressions and engagement following the prehabilitation phase, immediately before surgery. T4 (post-rehabilitation): to evaluate overall satisfaction after completion of the full intervention protocol (4 weeks postoperatively).

Measuring patient satisfaction at both stages of the intervention will provide important insights into adherence and participant engagement, factors that are critical to the success and future implementation of telerehabilitation in oncologic surgical pathways [86,87].

### Sample size calculation

The primary outcome of this trial is the 6MWT distance (meters). The sample size was calculated for a two-arm parallel randomized controlled trial using the standard formula for comparing two independent means [88]. Assumptions included a two-sided α = 0.05, statistical power (1 − β) = 0.80, a clinically relevant between-group difference (Δ) of 60 m, based on perioperative colorectal cancer studies reporting minimal clinically important differences of approximately 60–75 m in the 6MWT [7], and a standard deviation (σ) of 80 m, consistent with previous randomized prehabilitation trials in similar populations [5,89]. Under these parameters, the estimated requirement was 23 participants per group (n = 46 in total). To account for an anticipated dropout rate of approximately 20%, the final target sample size was increased to 28 participants per group, resulting in a total of 56 participants. This sample size is expected to provide adequate statistical power to detect clinically meaningful differences in functional capacity while mitigating the impact of potential attrition [90].

### Data management

A secure and structured database will be developed to systematically track each participant’s progress during the study, including all clinical assessments and outcome measures. Data will be recorded in real time and stored on password-protected, encrypted computers, with access strictly limited to authorized members of the research team directly involved in the study.

All participants will be assigned a unique identification code to ensure the confidentiality and anonymization of personal information. An independent researcher will oversee the data collection process to ensure protocol adherence, monitor data quality and completeness, and safeguard participant safety throughout the study.

Data analysis will be performed only after participant recruitment is complete and all outcome assessments have been finalized.

### Statistical analysis

All statistical analyses will be performed using the Statistical Package for the Social Sciences (SPSS), version 30.0 (IBM Corp., Armonk, NY, USA). A two-sided p-value < 0.05 will be considered statistically significant for all tests.

Descriptive statistics will be used to summarize the baseline characteristics and outcome variables. Categorical variables will be reported as frequencies and percentages, whereas continuous variables will be expressed as means and standard deviations (or medians and interquartile ranges, as appropriate), together with 95% confidence intervals. The Shapiro–Wilk test will be used to assess the normality of continuous variables. To verify baseline comparability between the two groups, parametric or non-parametric tests will be selected based on data distribution: t-tests or Mann–Whitney U for continuous variables, and Chi-square tests for categorical variables.

To evaluate the effect of the intervention over time, linear mixed-effects models will be applied to account for within-subject correlations in longitudinal data. The primary models will include participant-level random intercepts, with time modeled as a categorical fixed effect, together with group and the group-by-time interaction. The covariance structure will be selected on the basis of model fit and parsimony. Random slopes for time will be explored in sensitivity analyses if supported by model fit and data structure. Prespecified adjustment variables beyond the main fixed effects will include the stratification factors used in randomization, namely age and sex. When significant interaction effects are observed, Bonferroni-adjusted post hoc tests will be used to identify pairwise differences.

Between-group comparisons at each time point will be conducted using analysis of covariance (ANCOVA), adjusting for baseline values (T1). Differences in categorical outcomes will be analyzed using the Chi-square test or Fisher’s exact test, as appropriate.

All analyses will be conducted under the intention-to-treat (ITT) principle, including all randomized participants in the groups to which they were originally assigned, regardless of adherence or protocol deviations. Missing data will be handled using multiple imputation under a missing-at-random assumption, with the imputation model incorporating treatment group, time, baseline measures, and other variables associated with missingness, as appropriate.

Effect sizes will be calculated to quantify the magnitude of differences observed, using Cohen’s d for parametric comparisons and effect size r for non-parametric comparisons, both for within- and between-group analyses of the main outcomes.

### Ethical aspects and dissemination

This study will be conducted in full compliance with the ethical principles outlined in the Declaration of Helsinki and the guidelines for Good Clinical Practice (GCP). The protocol has been reviewed and approved by the Clinical Research Ethics Committee of Aragón (reference number: PI23/557) and has been prospectively registered in the ClinicalTrials.gov database (identifier: NCT06593678).

Prior to participation, all individuals will receive detailed verbal and written information about the study objectives, the procedures, the potential risks and benefits, and the data protection measures. Written informed consent will be obtained from each participant before enrollment in the study.

To ensure the data confidentiality, each participant will be assigned a unique alphanumeric identification code. All study data will be pseudonymized and stored in encrypted, password-protected digital databases accessible only to authorized study personnel. The final dataset will only be accessible to the statistician responsible for data analysis.

The findings of this study will be disseminated regardless of the direction or significance of the results. Dissemination will include peer-reviewed publications, scientific conference presentations, and open-access repositories, in accordance with the FAIR (Findable, Accessible, Interoperable, Reusable) data principles.

## Discussion

The main objective of this randomized controlled trial is to evaluate the effectiveness of an asynchronous telerehabilitation program delivered through the HEFORA digital platform, across both the prehabilitation and postoperative recovery phases, compared with a booklet-based usual-care program reflecting the perioperative pathway routinely available in the study setting before trial implementation. The study responds to the growing clinical need to mitigate perioperative deterioration while addressing the logistical and geographic barriers that often limit access to center-based rehabilitation programs. By integrating multimodal components into a home-based, digitally supported protocol, this trial proposes a patient-centered strategy that may improve continuity of care during one of the most vulnerable phases of the oncologic trajectory.

The 6MWT was selected as the primary outcome because it is a valid and clinically meaningful measure of functional capacity in surgical and oncology populations. Its clinical relevance is supported by consistent associations between improvements exceeding the minimal clinically important difference (MCID) and reduced postoperative morbidity and shorter hospital stays [5,91,92]. By assessing this variable at four perioperative time points, this study acknowledges the dynamic and non-linear functional trajectories described in patients with CRC [92]. At the same time, performance in the 6MWT may reflect not only physiological adaptation but also motivation, engagement, familiarization, and confidence fostered by ongoing support. Accordingly, any between-group differences in walking performance should be interpreted as potentially arising from a combination of physiological and behavioral mechanisms. To reduce the risk of differential assessment bias, performance-based outcomes will be administered by a blinded assessor using the same standardized instructions and procedures in both groups at each time point. This interpretation is further strengthened by the inclusion of additional objective outcomes, including muscle strength, spirometry, body composition, and ultrasound-based muscle assessment.

Muscle strength, measured using handgrip dynamometry and the 5R-STS, provides a complementary view of neuromuscular performance and frailty status. Reduced preoperative grip strength is a predictor of complications and delayed recovery [49,93]. Although short-term interventions may not yield large improvements, studies by Minnella et al. and Carli et al. suggest that even modest improvements can influence postoperative exercise capacity when combined with structured rehabilitation [94,95]. Recent evidence from an RCT in breast cancer survivors showed that a 16-week multimodal exercise program, including resistance training, delivered both online and in person significantly improved lean body mass, with no significant differences between delivery formats [96]. These results support the feasibility and potential effectiveness of remote resistance training interventions in oncology care and highlight the relevance of including upper and lower limb strength measurements in the evaluation of the physical impact of telerehabilitation.

Telerehabilitation is expected to positively influence key variables such as body composition, sarcopenia and visceral adiposity, all of which are closely associated with postoperative outcomes in CRC patients [43,97]. Sarcopenia and visceral adiposity have been independently associated with higher postoperative complication rates and lower overall survival in CRC [97,98]. The scientific literature supports that multimodal interventions, particularly those that combine exercise and nutritional support, can mitigate muscle loss and improve patients’ functional status [99,100]. Although Carli et al. [101] and Gillis et al. [26] have shown that such programs can reduce muscle loss, nutritional optimization is often required for sustained benefits, with real-time monitoring of muscle changes emphasizing the need for more sensitive, individualized tools as highlighted by Suárez-Alcázar et al. [102]. In parallel, psychological adaptation, measured with the MAC scale, and emotional distress, assessed with the HADS, will provide information on the psychosocial impact of the multimodal intervention. Maladaptive coping styles such as “helpless–hopelessness” have been associated with poorer recovery, whereas active strategies (e.g., “fighting spirit”) correlate with better treatment adherence and emotional resilience [26,68]. Although physical activity can alleviate symptoms of anxiety and depression [103], evidence from Wu et al. suggests that preoperative anxiety may persist despite functional improvement [104], justifying the inclusion of specific psychosocial support elements in telerehabilitation. However, telerehabilitation has shown promising results in improving psychological adaptation and reducing emotional distress in cancer patients [80,82,105].

Sleep quality plays a central role in cancer recovery, although it is often underestimated. The use of the PSQI will allow examination of the relationship between structured exercise and rest-activity patterns [64,66]. In oncology populations, poor sleep has been associated with fatigue, immune dysregulation, and impaired QoL [106]. Palesh et al. [107] demonstrated that physical activity interventions may lead to subjective improvement in sleep, although causality has not yet been established in CRC cohorts. Compared with booklet-based care without platform-enabled asynchronous monitoring and with lower supervision intensity, telerehabilitation provides more immediate, interactive, and personalized support, together with closer progression and symptom monitoring that has been associated with greater improvements in patient engagement, symptom management and recovery [82]. This approach may be particularly relevant in the context of sleep quality, where structured exercise and psychosocial support, delivered remotely could positively influence the rest-activity rhythms and reduce fatigue-related sleep disturbances [104].

The assessment of HRQoL using the EQ-5D-5L and the EQ-VAS will provide both a descriptive and self-perceived perspectives on overall health status. While studies such as those by Dowing et al. [108] and Borchert et al. [109] found that EQ-5D scores often remain relatively high in CRC survivors, self-perceived health status as reflected in the EQ-VAS may fluctuate in response to symptoms, pain, or mood disturbances. By capturing both domains across five time points, this study aims to provide a more nuanced understanding of recovery [110]. Multimodal prehabilitation and postoperative rehabilitation programs that combine physical exercise with nutritional and psychological support have consistently shown benefits in surgical oncology, particularly for functional capacity, emotional well-being, and overall QoL [99,101]. When delivered through telerehabilitation, these interventions may preserve clinical effectiveness while improving accessibility and adherence, thereby offering a scalable and patient-centered alternative to traditional models [86].

The IPAQ-SF will provide contextual data on patient engagement and behavior change. Perioperative decrements in physical activity are common among patients with colorectal cancer and other surgical oncology populations, but these declines can be mitigated through digital reinforcement strategies [62,111].

Similarly, patients´ treatment expectations and perceptions of the telerehabilitation intervention will be assessed using the Stanford Expectations of Treatment Scale, the TUQ, and a customized satisfaction survey. These tools will offer insight into acceptability, usability, and adherence, dimensions that are essential for evaluating real-world scalability. Parmanto et al. [81] and Orlando et al. [112] highlight that usability and perceived benefit are strong predictors of digital adherence, particularly in cancer survivors.

A key limitation of this trial is the brief two-week prehabilitation phase, which is determined by the usual interval between diagnosis and surgery in the study setting. Although this timeframe is clinically realistic, it may limit the extent of physiological gains achievable before surgery, particularly in relation to muscle strength and functional reserve. Accordingly, the prehabilitation component should be interpreted primarily as a pragmatic optimization window embedded within routine surgical care rather than as a stand-alone training period expected to produce substantial physiological adaptations in all participants. Additional limitations arise from the pragmatic comparator design. Both groups receive the same multimodal physiotherapy content, and both include physiotherapist-led follow-up; therefore, any between-group differences are more likely to reflect differences in delivery, monitoring, progression, reminders, and continuity of support than differences in exercise content itself. Because the telerehabilitation group receives more continuous platform-supported monitoring between visits, this design may introduce cointervention imbalance, performance bias, and some uncertainty in attributing observed effects specifically to the digital component in isolation. In addition, several secondary outcomes are derived from self-reported questionnaires and may therefore be influenced by expectancy effects, engagement bias, and novelty-related responses associated with digital delivery. To reduce misunderstanding during questionnaire completion, these instruments will be administered in a standardized manner in both groups, and the physiotherapist will be available only to clarify item wording and ensure consistent understanding rather than to guide responses. While these measures are necessary to assess psychosocial, behavioral, usability, and satisfaction-related domains that are not fully captured by objective performance-based assessments, their findings should be interpreted cautiously and in the context of the objective assessor-administered outcomes. Similarly, BIA-derived estimates may be influenced by perioperative shifts in hydration and inflammation and should therefore be interpreted alongside anthropometric and ultrasound-based assessments rather than as direct evidence of true changes in muscle or fat mass. Whenever feasible, BIA assessments will also be scheduled at the same time of day to reduce short-term variability related to fluid balance and daily fluctuations. Finally, this is a single-center study with a relatively small sample drawn from a selected population of patients undergoing elective colorectal cancer surgery. In addition, the exclusion of frail patients and adults older than 80 years may limit the generalizability of the findings to more vulnerable perioperative populations. Future multicenter studies including broader eligibility criteria and longer or more individualized prehabilitation periods will be necessary to establish the external validity of this intervention.

To date, no randomized controlled trial has explored asynchronous digital prehabilitation into the postoperative period using a multidimensional assessment strategy that includes physical, psychosocial, behavioral, and experiential outcomes. The present study addresses this gap by providing a structured, scalable model based on validated tools and evidence-based practices [101,113].

If this digitally enabled, patient-centered approach proves effective, it could redefine perioperative care in colorectal oncology, by promoting equitable access, supporting patient autonomy, and addressing both the physical and emotional dimensions of recovery [86,111]. In this way, it could pave the way for wider adoption of asynchronous telerehabilitation as a standard component of value-based cancer care [85].

## Conclusion

This RCT protocol presents an innovative, patient-centered strategy for integrating asynchronous telerehabilitation into the prehabilitation and postoperative recovery of individuals undergoing colorectal cancer surgery. The proposed intervention is based on current evidence and clinical needs and addresses key challenges in perioperative care, such as limited accessibility, lack of time and the need for personalized, multidimensional rehabilitation.

By combining structured therapeutic exercises, patient education and remote monitoring via a digital platform, this study aims to determine whether asynchronous delivery can lead to significant improvements in functional capacity, muscle strength, body composition, psychological adjustment, sleep quality, physical activity and HRQoL.

In contrast to traditional center-based programs, this model emphasizes autonomy, scalability and continuity of care, which may be particularly relevant for patients with logistical or mobility challenges. In addition, the comprehensive five-time point assessment design provides the opportunity to explore the course of recovery in detail and to identify the key phases in which support may be most effective.

If results confirm clinical effectiveness, this intervention could contribute to redefining standard perioperative procedures in oncology by providing an accessible and evidence-based alternative that empowers patients while optimizing outcomes. Ultimately, this protocol is a response to the demand for innovative, equitable and cost-effective rehabil

## Abbreviations

CRC: Colorectal Cancer
QoL: Quality of Life
WHO: World Health Organization
MDT: Multidisciplinary Team
TME: Total Mesorectal Excision
ERAS: Enhanced Recovery After Surgery
RCT: Randomized Controlled Trial
SPIRIT: Standard Protocol Items: Recommendations for Interventional Trials
CONSORT: Consolidated Standards of Reporting Trials
IG: Intervention Group
CG: Control Group
ASA: American Society of Anesthesiologists
T1: Pre-prehabilitation
T2: Post-prehabilitation
T3: Pre-rehabilitation
T4: Post-rehabilitation
T5: Follow-up
6MWT: Six-Minute Walk Test
ATS: American Thoracic Society
ERS: European Respiratory Society
BIA: Bioelectrical Impedance Analysis
ASIS: Anterior Superior Iliac Spine
CSA: Cross-Sectional Area
HGS: Handgrip Strength Test
5R-STS: Five-Repetition Sit-to-Stand Test
FVC: Forced Vital Capacity
FEV₁: Forced Expiratory Volume in 1 second
IPAQ-SF: International Physical Activity Questionnaire – Short Form
MET: Metabolic Equivalent Task
PSQI: Pittsburgh Sleep Quality Index
MAC: Mental Adjustment to Cancer
HADS: Hospital Anxiety and Depression Scale
HADS-A: Hospital Anxiety and Depression Scale-Anxiety
HADS-D: Hospital Anxiety and Depression Scale-Depression
HRQoL: Health-Related Quality of Life
EQ-5D: EuroQol-5D
EQ-VAS: Visual Analogue Scale
SETS: Stanford Expectations of Treatment Scale
TUQ: Telehealth Usability Questionnaire
ITT: Intention-to-Treat
LOCF: Last Observation Carried Forward
GCP: Good Clinical Practice
FAIR: Findable, Accessible, Interoperable, Reusable
MDC: Minimum Detectable Change
