# Correction: Asynchronous telerehabilitation in prehabilitation and postoperative recovery for colorectal cancer: A protocol for a randomized controlled trial

**DOI:** 10.1371/journal.pone.0350693

**Published:** 2026-06-03

**Authors:** The PLOS One Editors

## Notice of Republication

This article [1] was republished on May 13, 2026, to address an issue identified post-publication. Updated versions of S1 Appendix and S1–S4 Files are provided with this notice. Please download this article again to view the correct version.

In the Study Design subsection of the Methods section, the second sentence of the first paragraph is updated to: The protocol follows the Standard Protocol Items: Recommendations for Interventional Trials (SPIRIT) guidelines and is informed by the Consolidated Standards of Reporting Trials (CONSORT) 2010 recommendations for reporting randomized trials (S1 Appendix).

## Supporting information

S1 Appendix SPIRIT 2025 checklist of items to address in a randomized trial protocol. (DOCX)

S1 File Participant Information Sheet_English. (DOCX)

S2 File Participant Information Sheet_Spanish. (DOCX)

S3 File Project submitted to the ethics committee_English. (PDF)

S4 File Project submitted to the ethics committee_Spanish. (PDF)
