# Supplementary material for: Correction: Asynchronous telerehabilitation in prehabilitation and postoperative recovery for colorectal cancer: A protocol for a randomized controlled trial
Source: PLoS One. 2026 Jun 3;21(6):e0350693. doi: 10.1371/journal.pone.0350693 (PMC13232795; doi:10.1371/journal.pone.0350693)
Supplement: S1 File — (DOCX) [file pone.0350693.s002.docx]

**PARTICIPANT INFORMATION SHEET**

**Study Title:** “Tele-Rehabilitation in Oncology Patients: Optimization of Prehabilitation and Rehabilitation Following Colorectal Resection. Randomized Clinical Trial”.

**Sponsor:** University of Zaragoza.

**Principal Investigator:** José Manuel Burgos Bragado

**Telephone:** XXX XXX XXX **e-mail:** xxxxxxxxxxx@gmail.com

1. **Introduction:**

We are inviting you to take part in a research project being conducted by Universidad San Jorge in collaboration with Hospital Royo Villanova in Zaragoza. Your participation is entirely voluntary; under no circumstances should you feel obliged to take part. However, your involvement is important in order to obtain the knowledge we require. This project has been approved by the Ethics Committee of Aragón. Before making a decision, it is important that you:

- - read this document in full
  - understand the information it contains
  - ask any questions you may consider necessary
  - take time to carefully consider your decision
  - sign the informed consent form if you decide to participate

If you agree to participate, you will be given a copy of this information sheet and the signed consent form. Please keep them in case you need to refer to them in the future.

1. **Why are you being asked to participate?**

You are being invited to participate because you will undergo treatment and surgery for colorectal cancer at Hospital Royo Villanova in Zaragoza. This study plans to include a total of 56 patients with similar characteristics, all of whom are being treated at Hospital Royo Villanova in Zaragoza.

1. **What is the purpose of this study?**

The study aims to determine the impact of a home-based program combining exercise and health education, delivered either through a web application or via written information. The main objective is to evaluate the potential benefits of a home-based program in promoting physical well-being and quality of life in people who have undergone colorectal cancer surgery and have been discharged from the hospital.

1. **What will I need to do if I decide to participate?**

The program consists of performing an exercise and health education program for patients diagnosed with colorectal cancer. Some patients will follow a booklet containing conventional advice and exercises, while others will use an online application called **HEFORA**, provided free of charge by the Professional College of Physiotherapists of Aragón, available on computers and mobile phones for the same purpose. Both groups will receive the same treatment, but one will do so through the web application and the other through the printed booklet.

To protect your personal information, you will be assigned an alphanumeric code. This code is designed to represent you within the study but will not contain any identifiable personal data. This is done to ensure your privacy and confidentiality while participating in the study.

Participants will be randomly assigned to either the conventional or the intervention group. This random assignment is a fair and unbiased way of determining which group each participant will join. The assignment will be done using sealed envelopes prepared at Universidad de Zaragoza, which will be handed to participants in the order they join the study at Dr. Blas’s consultation at Hospital Royo Villanova.

If you agree to participate, meet the inclusion criteria, and sign the informed consent form, you will be scheduled on the same day as your appointment with the Chief Surgeon, Dr. Blas Juan Luis, at Hospital Royo Villanova for a prehabilitation functional assessment. On the day of your hospital admission for surgery, a second assessment will be performed. Approximately 21 days after surgery, a post-operative functional assessment will take place, and finally, a last evaluation will be carried out 50 days after surgery. Each of these four assessments will last approximately 30 minutes.

Please note that for these four assessments, you will need to attend the hospital on the same day as your appointment with the General and Digestive Surgery Department. Travel costs will not be reimbursed, as this study does not have financial support.

During the study, you will be asked to complete a diary, either in written form or digitally via a web platform, recording whether you completed the scheduled exercises, if they were done with the prescribed load (weight and repetitions), any additional exercise performed, and your level of fatigue (from 0 to 10) upon completion.

All participants will be informed about the physiotherapy treatment they will receive and will be able to ask questions at any time during the study. Participants in the intervention group will be able to send messages through the web platform to communicate with the physiotherapist, report fatigue levels during exercises, and resolve any issues with the program. Control group participants will have a phone number for one of the researchers to contact in case of any questions, problems, or concerns.

The program will last a total of 18 weeks, from group assignment and the first assessment to the final evaluation three months after surgery. All assessment, intervention, and follow-up sessions will be provided free of charge for all participants.

1. **What risks or inconveniences are involved?**

Participating in a therapeutic exercise and health education program does not involve any adverse effects if it is carried out according to the prescribed recommendations. Each participant will have the phone number of the principal investigators in case of any incident and will be able to communicate by phone with the researchers regarding any questions or adverse circumstances during the treatment. In addition, the HEFORA web platform includes a messaging channel where participants can report any such issues.

1. **Will I receive any benefits from participating?**

As this is a research study aimed at generating knowledge, you will not receive any direct personal benefit from your participation. However, your involvement will contribute to scientific advancement and social benefit. You will not receive any financial compensation for participating.

1. **How will my personal data be handled?**

**Basic information on data protection:**

Data Controller: University of Zaragoza.

Internal Responsible Party: Dra. Sandra Calvo (Principal Investigator of the Project)

Internal Processor: D. José Manuel Burgos Bragado (Investigator)

Purpose: Your personal data will be processed exclusively for the project or study referred to in this document. The processing of your personal data will be carried out using techniques to maintain your anonymity through the use of random codes, so that your personal identity remains completely concealed during the research process.

Legal Basis: The processing of data in this project or study is legitimized by your consent to participate.

Recipients: Data will not be shared with third parties unless legally required.

Duration: Personal data will be destroyed once the purpose for which it was collected has been fulfilled, including any possible audits or accountability reviews. The results, fully anonymized and containing no personal data, may be retained for possible reuse in other research projects. Based on the research results, scientific communications may be prepared for presentation at conferences or in scientific journals, always using aggregated data and never disclosing any information that could identify you.

Rights: You may exercise your rights to access, rectify, erase, and port your data, as well as to restrict or object to its processing, in accordance with the General Data Protection Regulation (GDPR), by contacting the internal responsible party for this project, whose contact details appear at the beginning of this document, or by emailing the Data Protection Officer of the University of Zaragoza (dpd@unizar.es). If your request is not addressed, you may file a complaint with the Spanish Data Protection Agency (https://www.aepd.es). Additional and detailed information on this data processing can be consulted in the Inventory of Processing Activities of the University of Zaragoza, accessible at: Inventario de actividades de tratamiento | Unidad de Protección de Datos (unizar.es).

As a user of the HEFORA platform, you have the right to access, rectify, and erase your data, as well as other rights indicated in the additional information, via email at [info@hefora.com](mailto:info@hefora.com). The platform will anonymously and encryptedly record the number of times you access it and view the videos. This data will be retained until the end of the study, at which point it will be provided to the principal investigators for analysis, solely for research purposes and while maintaining participants’ anonymity.

As a user of the HEFORA platform, you have the right to access, rectify, and erase your data, as well as other rights indicated in the additional information, via the email address [info@hefora.com](mailto:info@hefora.com).

Furthermore, in compliance with the GDPR, you are informed that, if you wish, you may contact the Spanish Data Protection Agency (<https://www.aepd.es>) to file a complaint if you believe that your rights have not been properly addressed.

The processing of your personal data will be carried out using techniques to maintain your anonymity through the use of random codes, ensuring that your personal identity remains completely concealed during the research process. No personal data will be collected on the HEFORA platform; it will only be used as a tool for exercise prescription, educational videos, and as a communication channel. Upon registration, you as a participant will only need to enter the code assigned to you in the study and an email address of your choice.

Based on the results of the research, scientific communications may be prepared for presentation at conferences or in scientific journals, always using aggregated data and never disclosing any information that could identify you.

Data from interviews and in-person assessments will be recorded by transferring the paper results into a data management system (data collection notebook).

1. **Who is funding the study?**

This project does not have external funding.

1. **Will I be informed of the study results?**

You have the right to be informed of the results of this study, both the overall findings and those derived from your specific data. You also have the right not to receive this information if you prefer. For this reason, the informed consent form will ask you which option you prefer. If you choose to receive the results, the investigator will provide them to you.

1. **Can I change my mind?**

Your participation is entirely voluntary. You may decide not to participate or to withdraw from the study at any time without giving any reason, and this will not affect your medical care. You only need to inform the principal investigator of the study. Additionally, if you choose to withdraw from the study at any time, you have the right to request that all personal data collected up to that point be deleted. The research team is committed to protecting your privacy and will respect your wish to have your information removed from the study if you so desire.

1. **What if I have any questions during my participation?**

The first page of this document lists the names and contact telephone numbers of the researchers responsible for the study. You may contact them if you have any questions regarding your participation.

Thank you very much for your attention. If you decide to participate, please sign the attached informed consent form.

**INFORMED CONSENT FORM**

**Project Title**: Tele-Rehabilitation in Oncology Patients: Optimization of Prehabilitation and Rehabilitation Following Colorectal Resection***.***

I, .............................................................................. (participant's full name)

have read the information sheet provided to me.

I have been able to ask questions about the study and have received sufficient information regarding it.

I have spoken with: José Manuel Burgos Bragado (name of the investigator)

I understand that my participation is voluntary.

I understand that I can withdraw from the study:

1) at any time

2) without giving any reason

3) without this affecting my medical care.

I freely give my consent to participate in this study and consent to the access and use of my data as outlined in the information sheet provided to me.

I wish to be informed of the study results: (tick as appropriate)

No

Si

I consent to my clinical data being reviewed by personnel outside the center for the purposes of this study, and I am aware that this consent is revocable.

I have received a signed copy of this Informed Consent Form.

| Participant’s signature: |  |
| --- | --- |
| Date: |  |

I have explained the nature and purpose of the study to the above-mentioned participant.

| Participant’s signature: |  |
| --- | --- |
| Date: |  |
