# Supplementary material for: Correction: Asynchronous telerehabilitation in prehabilitation and postoperative recovery for colorectal cancer: A protocol for a randomized controlled trial
Source: PLoS One. 2026 Jun 3;21(6):e0350693. doi: 10.1371/journal.pone.0350693 (PMC13232795; doi:10.1371/journal.pone.0350693)
Supplement: S2 File — (DOCX) [file pone.0350693.s003.docx]

**DOCUMENTO DE INFORMACIÓN PARA EL PARTICIPANTE**

**Título de la investigación:** “Tele-Rehabilitación en pacientes oncológicos: optimización de la prehabilitación y rehabilitación tras una resección colorrectal. Ensayo Clínico Aleatorizado”.

**Promotor:** Universidad de Zaragoza.

**Investigador Principal:** José Manuel Burgos Bragado

**Teléfono:** XXX XXX XXX **e-mail:** xxxxxxxxxxxxxxxxx@gmail.com

1. **Introducción:**

Nos dirigimos a usted para solicitar su participación en un proyecto de investigación que estamos realizando desde la Universidad San Jorge en colaboración con el Hospital Royo Villanova de Zaragoza. Su participación es absolutamente voluntaria, en ningún caso debe sentirse obligado a participar, pero es importante para obtener el conocimiento que necesitamos. Este proyecto ha sido aprobado por el Comité de Ética de Aragón. Antes de tomar una decisión es necesario que:

- - lea este documento entero
  - entienda la información que contiene el documento
  - haga todas las preguntas que considere necesarias
  - tome una decisión meditada
  - firme el consentimiento informado, si finalmente desea participar.

Si decide participar se le entregará una copia de esta hoja y del documento de consentimiento firmado. Por favor, consérvelo por si lo necesitara en un futuro.

1. **¿Por qué se le pide participar?**

Se le solicita su colaboración porque va a ser tratado e intervenido de cáncer colorrectal en el Hospital Royo Villanova de Zaragoza. En este estudio, se tiene prevista la participación de un total de 56 pacientes con características similares, todos ellos pertenecientes al Hospital Royo Villanova de Zaragoza.

1. **¿Cuál es el objeto de este estudio?**

El estudio busca determinar el impacto de la prescripción de un programa que combina ejercicios y educación para la salud que se realizan de forma domiciliaria mediante una aplicación web o mediante información escrita. El objetivo principal es evaluar los posibles beneficios potenciales de un programa domiciliario en la promoción del bienestar físico y la calidad de vida de las personas que han padecido un cáncer colorrectal y han sido dadas de alta hospitalaria tras la cirugía.

1. **¿Qué tengo que hacer si decido participar?**

El programa consistirá en la realización de un programa de ejercicios y educación para la salud en pacientes diagnosticados de cáncer colorrectal. Algunos pacientes seguirán un folleto con consejos y ejercicios convencionales, mientras que otros utilizarán una aplicación on-line llamada HEFORA que ofrece gratuitamente el Colegio Profesional de Fisioterapeutas de Aragón, disponible en ordenadores y teléfonos móviles, para el mismo propósito. Ambos grupos recibirán el mismo tratamiento, pero uno lo hará a través de la aplicación web y el otro mediante un folleto impreso.

Para mantener la privacidad de su información personal, le asignaremos un código alfanumérico. Este código está diseñado para representarle dentro del estudio, pero no contendrá ninguna información relevante sobre sus datos personales. Esto se hace para proteger su privacidad y confidencialidad mientras participa en el estudio.

Para decidir quién estará en cada grupo, usaremos un método llamado asignación aleatoria. Esto significa que su participación en el grupo convencional o en el grupo intervención será al azar. Es una forma justa y aleatoria de decidir quién va a formar parte de cada grupo en el estudio. Para ello, utilizaremos sobres cerrados que contienen la asignación a uno de los dos grupos. Estos sobres ya están preparados en la Universidad de Zaragoza y se entregarán a los participantes en el orden en que se unan al estudio en la consulta del Dr. Blas, en el Hospital Royo Villanova.

Si desea participar, cumple los criterios de inclusión, y firma el consentimiento informado, se le citará el mismo día de la cita con el cirujano jefe, Dr, Blas Juan Luis, en el Hospital Royo Villanova y se le realizará una evaluación funcional de prehabilitación. El día de su ingreso en el hospital para la cirugía, se le efectuará una segunda valoración. A continuación, aproximadamente a los 21 días después de la cirugía, se llevará a cabo una nueva valoración funcional postoperatoria y, finalmente, se realizará una última evaluación a los 50 días después de la cirugía. Las cuatro valoraciones tendrán una duración de 30 minutos aproximadamente cada una de ellas. Es importante informarle que, para estas cuatro valoraciones, se le citará el mismo día de la cita con el Servicio de Cirugía General y Aparato Digestivo, asumiendo que tendrá que desplazarse al Hospital Royo Villanova y que dichos desplazamientos no serán compensados económicamente ya que este estudio no tiene financiación económica. Además, durante el tiempo que dure el estudio se le pedirá que complete un diario bien de forma escrita o bien digitalmente, mediante una plataforma web, donde deberá registrar si ha completado los ejercicios programados, si los ha realizado con la carga (peso y repeticiones) indicada, si ha realizado ejercicio adicional al pautado y su nivel de fatiga (de 0 a 10) al terminarlos.

Todos los participantes serán informados del tratamiento fisioterapéutico que se les aplicará, y podrán realizar, en cualquier momento del estudio, las preguntas necesarias. Los pacientes del grupo intervención podrán enviar mensajes de control a través de la plataforma web para intercambiar mensajes entre fisioterapeuta-paciente y solucionar posibles problemas con la realización de los ejercicios propuestos, valorar el nivel de fatiga durante la realización del programa y analizar posibles incidencias. Por otro lado, los pacientes del grupo control tendrán un número de teléfono de uno de los investigadores para llamar si les surgiese algún contratiempo, duda o necesitasen cualquier aspecto.

El programa tendrá una duración total de 18 semanas desde que se le asigne un grupo y realice la primera evaluación hasta llevar a cabo la última evaluación a los 3 meses de la cirugía.

Todas las sesiones de evaluación, intervención y seguimiento se realizarán de forma gratuita para todos los participantes.

1. **¿Qué riesgos o molestias supone?**

La realización de un programa de ejercicio terapéutico y educación para la salud no presenta efectos adversos si se realiza siguiendo las recomendaciones pautadas. Cada paciente tendrá el teléfono de los investigadores principales en caso de cualquier incidencia y podrá comunicar telefónicamente con los investigadores para cualquier duda o circunstancia adversa durante el tratamiento. La plataforma web HEFORA cuenta además con un canal de mensajería donde reflejar cualquier circunstancia de esta índole.

1. **¿Obtendré algún beneficio por mi participación?**

Al tratarse de un estudio de investigación orientado a generar conocimiento no obtendrá ningún beneficio por su participación, si bien, usted contribuirá al avance científico y al beneficio social. Usted no recibirá ninguna compensación económica por su participación.

1. **¿Cómo se van a tratar mis datos personales?**

**Información básica sobre protección de datos.**

Responsable del tratamiento: Universidad de Zaragoza

Responsable interno: Dra. Sandra Calvo (Investigador Principal del Proyecto)

Encargado interno: D. José Manuel Burgos Bragado (Investigador)

Finalidad: sus datos personales serán tratados exclusivamente para el proyecto o estudio al que hace referencia este documento. El tratamiento de sus datos personales se realizará utilizando técnicas para mantener su anonimato mediante el uso de códigos aleatorios, con el fin de que su identidad personal quede completamente oculta durante el proceso de investigación.

Legitimación: el tratamiento de los datos de este proyecto o estudio queda legitimado por su consentimiento a participar.

Destinatarios: no se cederán datos a terceros salvo obligación legal.

Duración: los datos personales serán destruidos una vez se haya cumplido con la finalidad para la que se recabaron y para las posibles revisiones o determinación de responsabilidades. Los resultados objeto de explotación, ya completamente anonimizados y sin datos personales, podrán ser conservados para su posible reutilización en otros trabajos de investigación. A partir de los resultados de la investigación, se podrán elaborar comunicaciones científicas para ser presentadas en congresos o revistas científicas, pero se harán siempre con datos agrupados y nunca se divulgará nada que le pueda identificar.

Derechos: podrá ejercer sus derechos de acceso, rectificación, supresión y portabilidad de sus datos, de limitación y oposición a su tratamiento, de conformidad con lo dispuesto en el Reglamento General de Protección de Datos (RGPD) ante el responsable interno de este proyecto, cuyos datos de contacto figuran en el encabezamiento de este documento, o dirigiendo un correo electrónico al Delegado/a de Protección de Datos de la Universidad de Zaragoza (dpd@unizar.es). Si no viera atendida su petición podrá dirigirse en reclamación a la Agencia Española de Protección de Datos (https://www.aepd.es). Podrá consultar información adicional y detallada de este tratamiento de datos en el Inventario de Actividades de Tratamiento de la Universidad de Zaragoza, accesible en el siguiente enlace: Inventario de actividades de tratamiento | Unidad de Protección de Datos (unizar.es).

Como usuario de la plataforma HEFORA tiene derecho a acceder, rectificar y suprimir los datos, así como otros derechos indicados en la información adicional a través de dirección de correo electrónico [info@hefora.com](mailto:info@hefora.com). La plataforma recogerá de forma anónima y encriptada las veces que ha accedido a la plataforma y las veces que ha visualizado los vídeos. Estos datos los guardará hasta el final del estudio, momento en que los cederá a los investigadores principales para su análisis, únicamente con fines de investigación y manteniendo en todo momento el anonimato de los participantes.

Como usuario de la plataforma HEFORA tiene derecho a acceder, rectificar y suprimir los datos, así como otros derechos indicados en la información adicional a través de dirección de correo electrónico [info@hefora.com](mailto:info@hefora.com).

Así mismo, en cumplimiento de los dispuesto en el RGPD, se informa que, si así lo desea, podrá acudir a la Agencia de Protección de Datos (https://www.aepd.es) para presentar una reclamación cuando considere que no se hayan atendido debidamente sus derechos.

El tratamiento de sus datos personales se realizará utilizando técnicas para mantener su anonimato mediante el uso de códigos aleatorios, con el fin de que su identidad personal quede completamente oculta durante el proceso de investigación. En la plataforma HEFORA no se recogerá ningún dato personal, solo se utilizará como medio para la prescripción de los ejercicios, los vídeos educativos y como canal de comunicación. Usted como participante al registrarse solo ha de poner el código que le asignarán en el estudio y un email que usted decida.

A partir de los resultados del trabajo de investigación, se podrán elaborar comunicaciones científicas para ser presentadas en congresos o revistas científicas, pero se harán siempre con datos agrupados y nunca se divulgará nada que le pueda identificar.

Los datos de las entrevistas y valoraciones presenciales se realizarán mediante la incorporación de estos resultados en papel a un programa de tratamiento de datos (cuaderno de recogida de datos).

1. **¿Quién financia el estudio?**

Este proyecto no tiene financiación externa.

1. **¿Se me informará de los resultados del estudio?**

Usted tiene derecho a conocer los resultados del presente estudio, tanto los resultados generales como los derivados de sus datos específicos. También tiene derecho a no conocer dichos resultados si así lo desea. Por este motivo en el documento de consentimiento informado le preguntaremos qué opción prefiere. En caso de que desee conocer los resultados, el investigador le hará llegar los resultados.

1. **¿Puedo cambiar de opinión?**

Su participación es totalmente voluntaria, puede decidir no participar o retirarse del estudio en cualquier momento sin tener que dar explicaciones y sin que esto repercuta en su atención sanitaria. Basta con que le manifieste su intención al investigador principal del estudio. Además, si en cualquier momento decide retirarse del estudio, tiene el derecho de solicitar que se eliminen todos los datos personales que se hayan recopilado hasta el momento de su retirada del estudio. El equipo investigador está comprometido con su privacidad y respetará su deseo de retirar su información del estudio si así lo desea.

1. **¿Qué pasa si me surge alguna duda durante mi participación?**

En la primera página de este documento está recogido el nombre y el teléfono de contacto de los investigadores responsables del estudio. Puede dirigirse a él en caso de que le surja cualquier duda sobre su participación.

Muchas gracias por su atención, si finalmente desea participar le rogamos que firme el documento de consentimiento que se adjunta.

**DOCUMENTO DE CONSENTIMIENTO INFORMADO**

**Título del PROYECTO: “**Tele-Rehabilitación en pacientes oncológicos: optimización de la prehabilitación y rehabilitación tras una resección colorrectal”.

Yo, .............................................................................. (nombre y apellidos del participante)

He leído la hoja de información que se me ha entregado.

He podido hacer preguntas sobre el estudio y he recibido suficiente información sobre el mismo.

He hablado con: José Manuel Burgos Bragado (nombre del investigador)

Comprendo que mi participación es voluntaria.

Comprendo que puedo retirarme del estudio:

1) cuando quiera

2) sin tener que dar explicaciones

3) sin que esto repercuta en mis cuidados médico.

Presto libremente mi consentimiento para participar en este estudio y doy mi consentimiento para el acceso y utilización de mis datos conforme se estipula en la hoja de información que se me ha entregado.

Si

No

Deseo ser informado sobre los resultados del estudio: (marque lo que proceda)

Doy mi conformidad para que mis datos clínicos sean revisados por personal ajeno al centro, para los fines del estudio, y soy consciente de que este consentimiento es revocable.

He recibido una copia firmada de este Consentimiento Informado.

| Firma del participante: |  |
| --- | --- |
| Fecha: |  |

He explicado la naturaleza y el propósito del estudio al paciente mencionado

| Firma del Investigador: |  |
| --- | --- |
| Fecha: |  |
