# Supplementary material for: Correction: Asynchronous telerehabilitation in prehabilitation and postoperative recovery for colorectal cancer: A protocol for a randomized controlled trial
Source: PLoS One. 2026 Jun 3;21(6):e0350693. doi: 10.1371/journal.pone.0350693 (PMC13232795; doi:10.1371/journal.pone.0350693)
Supplement: S3 File — (PDF) [file pone.0350693.s004.pdf]

RESEARCH FOR CEICA

TITLE	**Tele-Rehabilitation in cancer patients: optimization of prehabilitation and rehabilitation after colorectal resection. Randomized Clinical Trial.**
VERSION AND DATE	Version 3. November 13, 2023
CENTER WHERE THE STUDY IS BEING CONDUCTED	**Hospital Royo Villanova, Zaragoza, Spain**

PRINCIPAL INVESTIGATOR OF THE PROJECT	
FIRST NAME AND LAST NAME*	José Manuel Burgos Bragado
ID	XXXXXXXXXX
EMAIL	xxxxxxxxxxx@usj.es
PHONE NUMBER*	XXX XXX XXX
JOB	University lecturer at San Jorge University.<br>PhD candidate at the University of Zaragoza.
SERVICE/DEPARTMENT	Bachelor's Degree in Physical Therapy
CENTER/FACULTY-UNIVERSITY/OTHER	SAN JORGE UNIVERSITY

SPONSOR (Essential for clinical trials and observational studies involving medicinal products)	
COMPANY IDENTIFICATION/ FIRST AND LAST NAME*	None
Tax ID/National ID*	
EMAIL*	
PHONE NUMBER*	
**CONTACT DETAILS OF THE PERSON RESPONSIBLE FOR MANAGING THE APPLICATION**<br>*If you wish, add the contact details of the person responsible for managing the details of the request*	
FIRST AND LAST NAME	José Manuel Burgos Bragado
EMAIL	jxxxxxxxxxxxxxxxxxxxx@gmail.com
PHONE	XXX XXX XXX

Any personal data contained in this communication will be incorporated into the processing system for which the Aragon Health Sciences Institute (IACS) is responsible. The data will be processed for the management and monitoring of studies evaluated by CEICA. The data will be deleted once the request has been responded to and/or processed and is no longer necessary. You have the right to access, rectify, and delete the data, as well as other rights granted to you by the data protection regulations before the IACS, with address at the Aragon Biomedical Research Center. Avda. San Juan Bosco, nº 13, 500009, Zaragoza, or by requesting it via email at protecciondedatos.iacs@aragon.es.

GLOSSARY

- Anonymization: process by which it is no longer possible to establish by reasonable means the link between a piece of data and the subject to which it refers. It is also applicable to biological samples.
- BIGAN: healthcare big data platform (managed by the IACS) that allows access to data from the Aragon Health System in pseudonymized form for use in management and research.
- Biobank: a public or private, non-profit establishment that houses one or more collections of biological samples of human origin for biomedical research purposes, organized as a technical unit with criteria of quality, order, and destination.
- Center: Institution where a study is conducted (hospital, health center, residence, faculty, private clinic, school, etc.). **In case of doubt, reference should be made to the place where the participants come from** (hospital, school, sports club, etc.).
- Confidentiality Agreement: Document that must be signed by students and residents who carry out any activity in the public health system (model established in Order SSI/81/2017).
- Informed consent: a valid expression of free and informed will by a capable person, or their authorized representative, preceded by adequate information.
- CEICA ruling: document certifying that the CEICA has evaluated a research project and that said project complies with applicable legal regulations and ethical criteria.
- Survey: collection of information in physical or digital format, with or without direct interaction with the source subject.
- Interview: collection of information with direct interaction with the source subject, through verbal responses.
- Research team: A group of researchers who jointly carry out a specific project.
- Primary data source: When data is collected directly from the study participant and for the purpose of the study.
- Secondary data source: When data already collected (and therefore recorded) for a purpose other than the study (health care, teaching, etc.) is used for the study.
- Research group: A group of researchers with a common background (publications, funding) led or coordinated by a Principal Investigator, grouped around a research topic and not necessarily by their healthcare or departmental affiliation.
- Participant information sheet: Document informing potential participants of the nature of the study so that they can give their informed consent.
- Intervention (intervention study): Any action to be performed on a person due to their participation in a study (this may be pharmacological treatment, physiotherapy, or an educational, behavioral, or psychological intervention).
- Principal investigator: The researcher who leads the project and is responsible for its design, implementation, and dissemination of results. If the study is multicenter, there must be a principal investigator at each center who is responsible for patients, data, and/or samples.
- Biological sample: any biological material of human origin that can be preserved and may contain information about a person's genetic makeup.
- Standard clinical practice: Procedures carried out purely for healthcare reasons, regardless of whether or not a person is participating in a research study.
- Research project: scientific procedure designed to gather information and formulate hypotheses about a particular social or scientific phenomenon.
- Sponsor: individual, company, institution, or organization responsible for initiating, managing, organizing, and financing a study.
- Pseudonymization: the processing of personal data in such a way that it can no longer be attributed to a data subject without the use of additional information, provided that such additional information is kept separately and is subject to technical and organizational measures to ensure that the personal data is not attributed to an identified or identifiable natural person.

SCOPE AND FINANCING OF THE PROJECT	
Is it a multicenter project? Yes☐ No ☒	If so, the complete list of centers must be submitted and a commitment form must be filled out by the research team<br>**for each center in Aragon** (see Annex I).
Do you have specific funding for the study? Y e s ☐ No ☒	In **all cases**, Annex II: Authorization for use of resources must be completed. In addition, if yes, the budget and source of<br>of funding.

2. STUDY CHARACTERISTICS
**2.1 Is this a drug trial?** Yes☐ No ☒
If yes, choose one option:<br><br>1) ☐ This is an observational study of drug treatment (EOM). In this case, specify:<br>☐ Prospective data collection☐ Retrospective data collection☐ Cross-sectional data collection<br><br>2) ☐ This is an intervention study: clinical trial with drugs<br>In this case, it must be submitted in accordance with the instructions of the AEMPS (https://www.aemps.gob.es/medicamentos-de-uso-humano/investigacionclinica_medicamentos/ensayosclinicos/#n-espanola)
**2.2 Is this research involving medical devices or medical products?** Yes☐ No ☒
If yes, choose an option:<br><br>1) ☐ This is an observational study on the use of the medical device<br><br>2) ☐ This is an intervention study: clinical trial with medical devices. In this case, it must be submitted in accordance with CEICA's PNT for this type of study (https://www.iacs.es/investigacion/comite-de-etica-de-la-investigacion-de-aragon-ceica/).

**2.3 Is this research involving invasive procedures?**<br>(Definition: any intervention carried out for research purposes that involves a physical or psychological risk to the participant).<br><br>If so, an insurance policy must be taken out or the risk must be justified as minimal.	Yes☐ No ☒
**2.4 Does the research include minors or persons incapable of giving their consent?**<br><br>If so, an information and informed consent document must be submitted to the guardian/legal representative/family member and another to the minor (adapted to their capacity). Review the CEICA template.	Yes☐ No ☒

2. STUDY CHARACTERISTICS	
**2.5 Are biological samples used in the study?**	Yes☐ No ☒
If yes, select one or more options:<br><br>1) ☐ Surplus samples from healthcare settings are used with consent for the project The information and consent document (CEICA template) must be submitted<br><br>2) ☐ Surplus samples from healthcare settings are used without consent This must be adequately justified in the section on ethical aspects (Art. 58.2 Law 14/2007).<br><br>3) ☐ Samples are collected specifically for this study. The information and consent document (CEICA template) must be presented.<br><br>4) ☐ A private sample collection is created. To create a new collection, submit the necessary documentation for evaluation and indicate the registration number (_________________) (see CEICA website).<br><br>5) ☐ Samples already collected in a private sample collection are used In this case, identify the collection number and person responsible:____________<br><br>6) ☐ Samples are requested from an authorized biobank. In this case, identify the biobank:_____ The request must be submitted to the biobank	
**2.6 Are genetic analyses performed?**	Yes☐ No ☒
**2.7 Are embryos, human embryonic cells, human fetal cells or tissues, or human pluripotent cells obtained through cell reprogramming used?**<br>You must contact the IACS or the responsible institution for further authorizations	Yes☐ No ☒

3. PROCESSING OF PERSONAL DATA

Personal data is considered to be any data (age, sex) or any information (numerical, alphabetical, graphic, acoustic) about an identified or identifiable natural person; any person whose identity can be determined, directly or indirectly (i.e., who has not been irreversibly anonymized at source) shall be considered identifiable.

**3.1 Is personal data collected or processed in the study?**	Yes☒ No ☐

If yes, check the applicable option:

☒The informed consent of the data subject is requested
Present the information and consent document (CEICA template)

☐Data obtained for another purpose that has been pseudonymized (e.g., medical history, other research, other records) is used in accordance with D.A. 17 of Organic Law 3/2018.

☐Other. Specify:

Check the **categories** to which the collected data belong:

☐ **Identifying data** (name, address, email, ID number, medical record number, telephone number, signature, IP address, geolocation, image/voice, others)

☒ **personal data:** date of birth, place of birth, parents' names, place of work, financial data, gender, marital status, children, academic qualifications, others.

☐ **Opinion data**

☐ **particularly sensitive data**: health, ethnicity, religion, political opinion, sexual life or orientation, union membership, special educational needs

3.2 If the data is collected directly from the data subject (primary source), specify the procedure
(Example: interview, paper survey, email, telephone, web applications, etc.)

Personal interview with the data subject.

3.3 If not collected directly from the data subject (secondary source), check the option and specify:

☐ Data from another similar study, for which the data subject's consent was obtained, is reused. Permission from the data controller, the consent form used to obtain consent, and the commitment to use pseudonymized data must be submitted (see website).

☐ Data obtained for another purpose and without consent for research is used (e.g., medical history or other records).
In this case, indicate:

☐ The researcher (if a member of staff at the center) has direct access to the medical record. Submit permission from the data controller (in the case of medical records, authorization from the management for access to data for this study must be submitted).

☐ The researcher receives the data already pseudonymized. Submit the commitment to use pseudonymized data (see website).

Note: **researchers who do** not **have an employment relationship with the hospital/center** do not have access to medical records, so ALWAYS when using this source, they must obtain pseudonymized data.

In all cases, explain: origin of the data, data controller

All data will be obtained from a personal interview with the data subject and transferred to the CRD in pseudonymized form. The CRDs will be stored in accordance with the appropriate protection measures.

☐ Data from BIGAN

Submit a report from the Biocomputation Unit (link to the request) and a commitment to use pseudonymized data (see website).

3.4 Once the information and data have been obtained, how is the privacy of participants guaranteed?

☐ Only aggregated data is used (i.e., data that corresponds to groups of people and not to each of those individuals).

☐ The data is anonymized (the data cannot be associated with an identified or identifiable person because the link to any information that identifies the subject has been irreversibly destroyed).

☒ The data is pseudonymized or encoded (direct identifiers are replaced by a code/pseudonym known only to the research team).

Explain how and who carries out the measure adopted:

Assignment of an alphanumeric identification code. The researcher José Manuel Burgos will carry out this procedure. All paper documents will be kept at the university, in a locked location. Pseudonymized data will be organized in an Excel spreadsheet and an SPSS file, which will be password-protected.

3.5 Data retention period: specify date of destruction:

September 2027, when all data extracted from this project is expected to have been published.

It is generally considered appropriate to retain data until publication; otherwise, justification must be provided.

3.6 Data processors (do not complete in the case of anonymous data)

A **data processor** is any natural or legal person, public authority, service, or other body that processes personal data on behalf of the data controller.

If a third party (outside the institution) processes project data, a **data processor agreement** must be signed. A template can be downloaded from https://seguridad.salud.aragon.es/plantillas/

Indicate which persons will process the data collected, specifying who will have access to the identifying data:

José Manuel Burgos will have access to all data through interviews with the individuals concerned, which will be collected at the CRDs and encoded with an alphanumeric code. José Manuel Burgos will be in charge of data processing, and Sandra Calvo will be responsible for data processing at the University of Zaragoza. The other researchers will only have access to pseudonymized data.

Are all persons authorized to process the data subject to a confidentiality agreement signed with the center?

Yes☒ No ☐

3.7 Will data be transferred to third parties? Yes☐ No ☒

If data is transferred, the following must be specified:

- the data transferred are:☐ identified,☐ pseudonymized,☐ anonymized

- to whom it is transferred:

- What data is transferred:

- for what purpose:

- Explain how the data is pseudonymized or anonymized:

Each user will receive an alphanumeric identification code in the study that will not include any data that could personally identify them (encoded CRD). The PI (José Manuel Burgos) will have a separate list that will allow the identification codes of the study participants to be linked to their personal details. This document will be stored on the researcher's corporate laptop with a private access code.

The PI will transfer the data of all study participants, collected in pseudonymized form on paper, to an Excel spreadsheet and an SPSS file, which will be made available to the researchers responsible for statistical data management.

- If there are international transfers: specify the company and country (in this case, the participant's express consent must be requested for this transfer).

PROCESSING OF PERSONAL DATA	
**3.8 Will recordings (audio/video) be made?**<br>Remember that the express consent of the data subject is required to make recordings; this information must be included in the consent document.	Yes☐ No ☒

In the case of recording, the following must be specified:

- Where they will be stored, who has access to them, and the security measures to be applied:

- Retention period for the recordings:

- for what purpose:

- If computer applications or cloud storage are used, the service provider and its legal residence must be indicated, as well as a link to its privacy policy:

3.9 Information security measures: description of the computer systems to be used

Please note that servers containing personal data must be located within the EU (RDL 14/2019).

- System on which the data will be stored (personal computer, corporate servers, external company or organization, cloud service provider, etc.)

Unizar corporate computer with inventory number 258280. Pseudonymized data in the cloud (access to Google Drive website for Unizar accounts), accessible via the researchers' corporate emails and passwords. This pseudonymized data will be organized in an Excel spreadsheet and an SPSS file, which will contain a password for access.

- Applications to be used for data processing (Excel, SPSS, etc.) will be

password protected.

- If online computer applications or cloud storage are used, the service provider and its legal residence must be indicated, as well as a link to its privacy policy.

Service provider: Microsoft. https://policies.google.com/privacy. Google Ireland Limited for users of Google services located in the European Economic Area or Switzerland, with registered office at Gordon House, Barrow Street, Dublin 4, Ireland.

3.10 Information security measures: Devices

- Indicate whether any type of removable device (portable USB, external hard drive, etc.) will be used and whether they will be encrypted

Backup on José Manuel Burgos' removable external hard drive, documents encrypted with zip, using a compressed folder and access password.

- If corporate computer systems are not used, indicate whether backups are made.

- Indicate the security measures for paper documents (custody, access). Paper documents

will be kept at the university, in a locked location.

General recommendations on data use

- Do not use Wi-Fi networks to transmit sensitive information.

- Use strong passwords and change them regularly.
- Always encrypt sensitive information that is to be sent by email.
- Ensure that operating system and application versions are always up to date.
- On personal computers, always use antivirus software and keep it up to date.
- Never open email attachments from senders you do not recognize.
- Do not use social media to communicate sensitive information.
- Mobile phones are not secure devices for handling sensitive information, and the antivirus software that can be installed on them offers little protection.
- Use corporate applications whenever possible.
- The use of USB or other removable devices is strongly discouraged.

4. DESCRIPTION OF THE RESEARCH PROJECT (complete the fields or attach the complete protocol with the equivalent information)

4.1 Tasks of the research team
Briefly explain who is participating in the study, in what capacity, and what tasks they will perform, as well as their affiliation (current job position). The CV and signature of all participants must be submitted in Annex I
If the study is multicenter, an Annex I must be submitted for each center.

- **José Manuel Burgos Bragado**. Physical therapist. Professor of the Bachelor's Degree in Physical Therapy. Faculty of Health Sciences. San Jorge University. iPhysio Research Group, San Jorge University of Zaragoza. *iHealthy* Research Group of the Aragon Health Research Institute (IISA25). PhD candidate at the University of Zaragoza.

  Tasks: Principal Investigator. Preparation of the report. Selection of participants and pseudonymization of the same. Carrying out the study intervention.
- **Sandra Calvo Carrión**. Physiotherapist. Lecturer in the Bachelor's Degree in Physiotherapy. Faculty of Health Sciences. University of Zaragoza. *iHealthy* Research Group at the Aragon Health Research Institute (IISA25).

  Tasks: Researcher. Methodological design. Final review of the report and future publications.
- **Carolina Jiménez Sánchez**. Physical therapist. Professor of Physical Therapy. Faculty of Health Sciences. San Jorge University. iPhysio Research Group, San Jorge University of Zaragoza. *iHealthy* Research Group of the Aragon Health Research Institute (IISA25).

  Tasks: Researcher. Methodological design. Analysis of results. Final review of the report and future publications.
- **Juan Luis Blas Laina.** Head of the General and Digestive Surgery Department, Royo Villanova Hospital, Zaragoza.

  Tasks: Researcher. Recruitment of study participants.
- **Paula Gracia Gimeno**. Graduate in Medicine and Surgery from the Faculty of Medicine of Zaragoza. Doctor in the Endocrinology and Nutrition Department, Royo Villanova Hospital in Zaragoza.

  Tasks: Researcher. Recruitment of study participants.
- **Jorge Alamillo Salas**. Bachelor of Medicine and Surgery from the Faculty of Medicine of Zaragoza. Physician in the Rehabilitation Department, Royo Villanova Hospital, Zaragoza.

Tasks: Researcher. Recruitment of study participants.

- **Daniel Fernández Sanchis**. Pharmacist and Nutritionist. Professor of Pharmacy. Faculty of Health Sciences. San Jorge University. iHealthy Research Group of the Aragon Health Research Institute (IISA25).

  Tasks: Researcher. Preparation of cost-effectiveness reports.

- **Beatriz Carpallo Porcar**. Physical therapist. Professor of Physical Therapy. Faculty of Health Sciences. San Jorge University. iPhysio Research Group, San Jorge University of Zaragoza. iHealthy Research Group of the Aragon Health Research Institute (IISA25).

  Tasks: Researcher. Conducting assessments of study participants.

- **Natalia Brandín de la Cruz**. Physiotherapist. Lecturer on the Bachelor's Degree in Physiotherapy. Faculty of Health Sciences. San Jorge University. iPhysio Research Group, San Jorge University of Zaragoza. *iHealthy* Research Group of the Aragon Health Research Institute (IISA25).

  Tasks: Researcher. Conducting assessments of study participants.

4.2 Justification of the study: Background, current status of the issue, relevance (Cite bibliographic references in the following section)

Over the last few decades, advances in anticancer therapies, including surgical techniques, radiotherapy, and cytotoxic drugs, have achieved significant progress in terms of increased survival and decreased local recurrence in colorectal cancer (CRC), avoiding the need for a colostomy (1.Araghi et al. 2019). However, this vital medical progress has not been accompanied by an improvement in the functional outcomes of patients undergoing surgery, who commonly experience postoperative complications (POC) and a notable decrease in their quality of life (2.Abarca et al. 2021). This type of malignant tumor places a significant burden on primary care and hospital systems and also has a dramatic impact on the workplace (3.Koopmanschap et al. 2008).

CRC is a malignant tumor that appears when a polyp develops in the colon or rectum. It is the second most prevalent malignant neoplasm and the fourth leading cause of cancer death worldwide, with nearly 1.9 million new cases and 935,000 deaths in 2020 (4.Gupta et al. 2020). In 2017, a total of 41,441 new cases of CRC were diagnosed in Spain, and 14,700 deaths related to this disease were recorded. However, the Spanish Society of Medical Oncology (SEOM) points out that, in general, cancer survival in Spain has increased significantly in recent decades thanks to therapeutic advances, preventive measures, and screening or early detection programs. In addition, a clear positive correlation has been observed between the survival of cancer patients and participation in therapeutic exercise activities, with an emphasis on aerobic work, as a factor in reducing mortality. After surgery, it is common for patients to experience a decrease in functional capacity due to systemic inflammation and surgical stress (5.Carli et al. 2015). Generally, recovery of basic physical performance levels can take several months. Consequently, insufficient physical functional capacity has been identified as an important predictor of morbidity and mortality after cancer surgery (6.Pouwels et al. 2015). That is why the process of improving functional capacity before surgery, in order to optimize physiological reserves and cope with surgical stress, has been termed "prehabilitation" (7.Gillis et al. 2014).

Prehabilitation is a cutting-edge tool in cancer treatment, designed as a single or multi-modal intervention with the aim of reducing complications and the length of hospital stays.

hospitalization and overall costs, as well as improving the results of the intervention and patients' quality of life. Prehabilitation can address both physical and mental recovery, as well as tailor nutritional advice to the patient (8.Chen et al., 2017). The concept of prehabilitation is based on the principle that structured and sustained exercise over a period of weeks leads to improved cardiovascular, respiratory, and muscular conditioning, associated with a lower rate of postoperative complications (9.Kulkarni et al., 2010) and earlier restoration of functional capacity (6.Pouwels et al., 2015). This concept is strongly related to the observation of poor physical condition, as well as other modifiable factors such as preoperative anemia and nutritional status, which are risk factors linked to serious postoperative complications (10. Bojesen et al., 2022). In the case of CRC, there are a large number of postoperative complications, with a 30% to 40% decrease in functional walking ability after surgery in others. Therefore, physical therapy within the prehabilitation phase in cancer patients is one of the main pillars for improving postoperative outcomes, through functional exercise programs and therapeutic education.

Within this rehabilitation treatment, therapeutic exercise for cancer patients undergoing surgery has been proven to be safe, acceptable, and feasible, with statistically significant improvements in functional capacity and respiratory capacity after surgery (11.Awasthi et al., 2019). Several studies have examined various factors relevant to exercise planning during this process. In particular, it has been observed that active, supervised physical exercise produces changes in functional capacity, i.e., it accelerates the postoperative return to initial activities to a greater extent than unsupervised exercise (12.Gomez et al., 2016).

The treatment of CRC in a public health system hospital in Spain has a considerable **socioeconomic impact**, both in terms of direct and indirect costs, which can generate a significant long-term economic burden. In a retrospective study that evaluated resource use and direct medical costs in a cohort of 699 patients diagnosed and treated for CRC during the period 2000-2006, variations in costs were observed depending on the stage at diagnosis and the stage of care. In the diagnostic phase, it ranged from €20,708 (in situ) to €47,681 (stage III) as a long-term average. On the other hand, in terms of treatment stages, the average cost of the initial period represented 24.8% of the total long-term average cost, while the costs associated with the continuous and advanced care stages represented 16.9% and 58.3%, respectively (Corral et al., 2016). The patient population is constantly increasing, which **could have significant economic and social implications for** national **health systems**. The introduction of new therapeutic strategies not only has the potential to improve individuals' quality of life, but also represents an interesting opportunity to reduce healthcare costs.

CRC offers a variety of treatment options; however, many conventional therapies still lack solid evidence of their effectiveness. This adds complexity and prolongs the process of managing this disease. Given these circumstances, **there is an urgent need to develop additional strategies for prevention and prehabilitation, as well as for post-surgical cancer treatment and nutritional counseling**. Current care in the public health system for CRC patients is often limited by challenges related to treatment efficacy, waiting times, and the quality of care provided, as well as others related to economic viability or the need for patients to live in close proximity to the relevant healthcare facilities (14.Haines et al., 2010).

For all these reasons, **telerehabilitation, which is based on the provision of remote rehabilitation services through the use of digital technology, is presented as an innovative alternative** (15.Russell et al., 2007), offering solutions to overcome challenges related to distance, time, and costs (16.McCue et al., 2010). This modality has the potential to significantly improve access to physical therapy, especially for those living in rural areas, while also enabling rapid and effective communication with physical therapists.

rapid and effective communication with physical therapists. In addition, it contributes to reducing costs and waiting lists in health systems at the national level.

Given the significant impact that cancer treatment has on patients' quality of life, access to **tele-rehabilitation systems** that facilitate the assessment and treatment of the sequelae resulting from this process becomes a **very useful tool for encouraging patients to actively participate in their own care**. These systems can be used for prevention, information, diagnosis, and continuous monitoring, providing immediate *feedback* on the evolution of the patient's condition. One of the significant challenges of conventional rehabilitation is the lack of adherence by patients, a barrier that telerehabilitation has the potential to overcome (17.Sabaté et al., 2003).

For all the above reasons, demonstrating the clinical efficacy of telerehabilitation programs compared to conventional rehabilitation is vitally important in order to assess their impact on the quality of life of cancer patients. This innovative strategy represents an alternative for improving the quality of life of cancer patients. Studies have demonstrated the feasibility and cost-effectiveness of these programs in different populations, such as diabetic patients (18.Kesavadev et al., 2012) and newborns (19.Isetta et al., 2013).

Thus, the implementation of telerehabilitation would represent a significant improvement in the care process at the Royo Villanova Hospital in Sector 1 of the SALUD and would also serve as an innovative model within the public health system, as there are currently no similar projects in other public centers in Aragon.

4.3 Bibliography (must be referenced in the text above)

1. Araghi, M., Soerjomataram, I., Jenkins, M., Brierley, J., Morris, E., Bray, F., & Arnold, M. (2019). Global trends in colorectal cancer mortality: projections to the year 2035. *International journal of cancer*, *144*(12), 2992–3000.
2. Abarca, Carmen Paz, & Fernández A., Macarena. (2021). Low anterior resection syndrome in patients with middle and lower rectal cancer: What else can we do? *Journal of Surgery*, *73*(1), 80-90
3. Koopmanschap, M. A., van Exel, J. N., van den Berg, B., & Brouwer, W. B. (2008). An overview of methods and applications to value informal care in economic evaluations of healthcare. *PharmacoEconomics*, *26*(4), 269–280.
4. Gupta, S., Coronado, G. D., Argenbright, K., Brenner, A. T., Castañeda, S. F., Dominitz, J. A., Green, B., Issaka, R. B., Levin, T. R., Reuland, D. S., Richardson, L. C., Robertson, D. J., Singal, A. G., & Pignone, M. (2020). Mailed fecal immunochemical test outreach for colorectal cancer screening: Summary of a Centers for Disease Control and Prevention-sponsored Summit. *CA: a cancer journal for clinicians, 70(*4), 283–298.
5. Carli, F., & Scheede-Bergdahl, C. (2015). Prehabilitation to enhance perioperative care. *Anesthesiology clinics*, *33*(1), 17–33.
6. Pouwels, S., Fiddelaers, J., Teijink, J. A., Woorst, J. F., Siebenga, J., & Smeenk, F. W. (2015). Preoperative exercise therapy in lung surgery patients: A systematic review. *Respiratory medicine*, *109*(12), 1495–1504.
7. Gillis, C., Li, C., Lee, L., Awasthi, R., Augustin, B., Gamsa, A., Liberman, A. S., Stein, B., Charlebois, P., Feldman, L. S., & Carli, F. (2014). Prehabilitation versus rehabilitation: a randomized control trial in patients undergoing colorectal resection for cancer. *Anesthesiology, 121(*5), 937–947.
8. Chen, B. P., Awasthi, R., Sweet, S. N., Minnella, E. M., Bergdahl, A., Santa Mina, D., Carli, F., & Scheede-Bergdahl, C. (2017). Four-week prehabilitation program is sufficient to modify exercise behaviors and

improve preoperative functional walking capacity in patients with colorectal cancer. *Supportive care in cancer: official journal of the Multinational Association of Supportive Care in Cancer*, *25*(1), 33–40.
9. Kulkarni, S. R., Fletcher, E., McConnell, A. K., Poskitt, K. R., & Whyman, M. R. (2010). Pre-operative inspiratory muscle training preserves postoperative inspiratory muscle strength following major abdominal surgery - a randomized pilot study. *Annals of the Royal College of Surgeons of England, 92(*8), 700–707.
10. Bojesen, R. D., Grube, C., Buzquurz, F., Miedzianogora, R. E. G., Eriksen, J. R., & Gögenur, I. (2022). Effect of modifying high-risk factors and prehabilitation on the outcomes of colorectal cancer surgery: controlled before and after study. *BJS open,* 6*(*3), zrac029.
11. Awasthi, R., Minnella, E. M., Ferreira, V., Ramanakumar, A. V., Scheede-Bergdahl, C., & Carli, F. (2019). Supervised exercise training with multimodal pre-habilitation leads to earlier functional recovery following colorectal cancer resection. *Acta anaesthesiologica Scandinavica, 63(*4), 461–467.
12. Gomez, I., Szekanecz, É., Szekanecz, Z., & Bender, T. (2016). Daganatos betegek fizioterápiája [Physio-therapy of cancer patients]. *Orvosi hetilap*, *157*(31), 1224–1231.
13. Corral, J., Castells, X., Molins, E., Chiarello, P., Borras, J. M., & Cots, F. (2016). Long-term costs of colorectal cancer treatment in Spain. *BMC health services research*, *16*, 56.
14. Haines, T. P., Sinnamon, P., Wetzig, N. G., Lehman, M., Walpole, E., Pratt, T., & Smith, A. (2010). Multi-modal exercise improves quality of life of women being treated for breast cancer, but at what cost? Randomized trial with economic evaluation. *Breast cancer research and treatment, 124(*1), 163–175.
15. Russell T. G. (2007). Physical rehabilitation using telemedicine. *Journal of telemedicine and tele-care*, *13*(5), 217–220.
16. McCue, M., Fairman, A., & Pramuka, M. (2010). Enhancing quality of life through telerehabilitation. *Physical medicine and rehabilitation clinics of North America*, *21*(1), 195–205.
17. Burkhart, P. V., & Sabaté, E. (2003). Adherence to long-term therapies: evidence for action. *Journal of nursing scholarship: an official publication of Sigma Theta Tau International Honor Society of Nursing*, *35*(3), 207.
18. Kesavadev, J., Shankar, A., Pillai, P. B., Krishnan, G., & Jothydev, S. (2012). Cost-effective use of tele-medicine and self-monitoring of blood glucose via Diabetes Tele Management System (DTMS) to achieve target glycosylated hemoglobin values without serious symptomatic hypoglycemia in 1,000 subjects with type 2 diabetes mellitus--a retrospective study. *Diabetes technology & therapeutics, 14(*9), 772–776.
19. Isetta, V., Lopez-Agustina, C., Lopez-Bernal, E., Amat, M., Vila, M., Valls, C., Navajas, D., & Farre, R. (2013). Cost-effectiveness of a new internet-based monitoring tool for neonatal post-discharge home care. *Journal of Medical Internet Research, 15(*2), e38.

4.4 Hypothesis (statement to be proven)

With regard to CCR surgery and its impact on quality of life, the effects of a physiotherapy intervention with telerehabilitation, complemented by the usual care in conventional treatment, can reduce postoperative complications and hospital stay, allowing the patient to achieve an optimal functional status and, therefore, a speedy recovery of their quality of life, as well as providing improved care for patients in general.

- **H_0:** The implementation of a physical therapy program carried out through asynchronous telerehabilitation, compared to the conventional group, does not reduce postoperative complications.
- **H_1:** The implementation of a physiotherapy program carried out through asynchronous telerehabilitation, compared to the conventional group, reduces postoperative complications.

4.5 Objectives

Primary:

- To determine whether the asynchronous telerehabilitation program, with prehabilitation and post-surgical rehabilitation, is more effective than conventional treatment in patients undergoing surgery for colorectal cancer, in reducing postoperative complications.

Secondary:

- To determine whether asynchronous telerehabilitation produces changes that improve body composition.
- Assess whether asynchronous telerehabilitation improves aspects related to cardiorespiratory capacity, muscle strength, and functional capacity.
- To analyze whether asynchronous telerehabilitation improves psychosocial aspects in patients with cancer.
- Identify whether asynchronous telerehabilitation improves patients' health-related quality of life.
- Determine whether asynchronous telerehabilitation increases adherence to the multimodal protocol through recruitment and satisfaction rates.
- Evaluate whether asynchronous telerehabilitation improves the acceptability of the treatment provided and the usability of telemedicine in healthcare terms.
- Analyze the feasibility and cost-effectiveness of the physical therapy program through tele-rehabilitation and determine the budgetary impact of its implementation at Royo Villanova Hospital.

4.6 Methodology (**all** of the following fields must be detailed**):**
Study design
Participants: inclusion/exclusion criteria; recruitment method (who makes initial contact with participants and how, presentation of study information material, if any), sample size (and justification), randomization (if applicable)
Sources of information: detailed variables (data to be collected), origin of the data, when and how it is collected, what time period it refers to.
Procedures: clearly differentiate between purely healthcare procedures and those specific to the research, present surveys or forms to be used (link in the case of online surveys), risk assessment of experimental procedures and measures to minimize risk.
Statistical analysis Limitations
of the study
In the case of biological samples: specify the type and number of samples, how they are collected, where and who analyzes them
analyzed, when they are destroyed (or final destination).

Design:
The project will consist of a simple blind Randomized Clinical Trial (RCT) with blinded evaluators.

Participants:
The recruitment strategy will be carried out in the General and Digestive Surgery Department at Royo Villanova Hospital in Zaragoza with patients diagnosed with CRC, under the supervision of Dr. BLAS Juan Luis, head of surgery at Royo Villanova Hospital. A parallel group design will be used, so each participant will be exposed to only one intervention, differentiating between a conventional program through a guidebook and an experimental program through an *online* tele-rehabilitation platform.

Among the participants recruited by Dr. BLAS, a minimum number of patients diagnosed with CRC will also be patients who attend the clinic of Dr. GRACIA Paola (physician in the Endocrinology and Nutrition Section of CME Grande Covián at Royo Villanova Hospital), who treats patients at medium and high nutritional risk or who have some type of nutritional deficiency. Therefore, we will have patients diagnosed only with CRC and in good nutritional status, and patients diagnosed with CRC and with a nutritional deficit. For this reason, we will seek to balance the groups with the presence of these two groups of patients, and subsequently, in the statistical analysis, these patients will be analyzed in a secondary analysis.

The sample size was calculated using existing bibliographic data on the *Comprehensive Complication Index* (CCI) scale, which has a high degree of reliability among evaluators (Slankamenac et al., 2014). The following values were taken into account:

- *Alpha* value: 0.05.
- Power value: 0.8.
- Beta error: 0.2.
- Minimum difference in means: 22.
- Standard deviation: 18.
- Due to possible loss of subjects: the total sample size is increased by 30%.

The study will include a total of 80 patients, divided into two groups:

- Control or conventional group: 40 patients.
- Intervention or experimental group: 40 patients.

Both groups will be correlated to determine the significant relationship between tele-rehabilitation physiotherapy intervention and improvement in the parameters to be evaluated. Descriptive statistics will also be used to report on the feasibility of recruitment and adherence to the rehabilitation program. The effect size will be calculated using *Cohen's d* to determine clinical significance.

Inclusion criteria:

- Age between 18 and 80 years.
- Participants who understand Spanish.
- Patients undergoing scheduled CCR surgery at Royo Villanova Hospital.
- Patients attending their first consultation at the General and Digestive Surgery Department, under the supervision of Dr. Blas, head of surgery at Royo Villanova Hospital in Zaragoza.
- Participants with functional independence that allows them to perform walking and pulmonary function tests.
- Patients with a preoperative *American Society of Anesthesiologists* (ASA) score of I, II, or III.
- Participants who agree to participate and sign the informed consent form.

Exclusion criteria:

- Patients over 80 years of age.
- Patients with an *ASA* preoperative assessment score of IV.
- Patients suffering from any injury, pathology, or inflammatory processes that make it impossible to exercise.
- Patients with central and/or peripheral neurological diseases that prevent them from following the rehabilitation program.
- Patients with unstable cardiac comorbidities, such as arrhythmias, high blood pressure, angina pectoris, or other pathologies that contraindicate moderate-intensity training.
- Patients diagnosed with psychiatric illness, as determined by a psychiatrist.
- Patients without access to mobile internet or a computer with internet at home.
- Patients who score≤ 24 on the *Mini-Mental State Examination* (MMSE).
- Individuals who are unable to follow oral and written instructions in Spanish.
- Patients who refuse to participate in the study or who have not signed the informed consent forms.

Discontinuation criteria:

- The patient's own decision to withdraw from the study.
- Lack of commitment to treatment (< 80% participation rate).

Randomization:
Once the patient meets the inclusion criteria specified above, randomization will be performed using a computer program and will be carried out by age group and gender.
Participants will sign their written informed consent and will be randomly assigned to the control group or the intervention group using sealed envelopes prepared at the University of Zaragoza, distributed in chronological order of recruitment at the office of Dr. BLAS Juan Luis.

Data will be collected to evaluate the effectiveness of prehabilitation and post-surgery rehabilitation treatment compared to the conventional program during the period of up to 30 days after colorectal cancer surgery. In addition, these patients will be followed up for 3 months after discharge to analyze the percentage of postoperative complications after this rehabilitation treatment.

Sources of information:
How will the study be conducted?
Patients diagnosed with CRC will be summoned and given the *American Society of Anesthesiologists* (ASA) preoperative assessment scale. All patients who meet the inclusion criteria will be randomized and placed in two parallel groups, so that each participant will be exposed to only one intervention:

- Control group: will receive a conventional program through a guidebook with guidelines on therapeutic education, breathing exercises, aerobic exercises, and therapeutic strength training.
- Intervention group: will receive an experimental program consisting of the same treatment as

The control group, on the other hand, will be monitored via a digital tele-rehabilitation platform. The physical therapist will install the tele-rehabilitation application together with the patient, check that it is working properly, and explain to the patient how to use it (the platform will not collect any personal data; the patient will install the platform using their identification code and will have access to the entire rehabilitation program, but at no time will they be required to record any personal data). The therapeutic education session and the various videos in the exercise program will be asynchronous.

All participants will be informed of the physiotherapy treatment they will receive and will be able to ask any questions they may have at any time during the study. Patients in the intervention group will be able to send control messages via the web platform to exchange messages between physiotherapist and patient and resolve any problems with performing the proposed exercises, assess the level of fatigue during the program, and analyze any incidents. On the other hand, patients in the control group will have instructions in the brochure on how to assess their level of fatigue and how to progress with the exercises, as well as a phone number for one of the researchers to call if they encounter any setbacks, have any questions, or need any assistance.

The rehabilitation program will be the same for both groups of patients, with the only difference being the channel of delivery (asynchronous telerehabilitation or brochure) and communication (messages on the platform or researcher's phone). It will be carried out over a period of six weeks (two weeks of prehabilitation and four weeks post-surgery), with sessions scheduled four days a week. As mentioned above, the program will consist of the following for both groups:

- Therapeutic education on healthy habits, facilitating early active movement exercises and walking to prevent complications such as atelectasis
  and deep vein thrombosis (DVT). It also includes health education for the patient on wound care and other aspects of self-care that they will need when they return home.
- Respiratory physiotherapy exercises will be performed slowly and in a relaxed manner, in a comfortable position and in a comfortable environment. The activities to be performed include abdominal-diaphragmatic breathing exercises
  and rib cage expansion with directed breathing.

On the other hand, the dynamic exercise program includes three different intensity levels, each consisting of between three and six exercises, following the WHO guidelines on Physical Activity and Sedentary Behaviors:

- Aerobic exercise, accumulating a minimum of 150 to 300 minutes of moderate-intensity aerobic activity throughout the week, or a minimum of 75 to 150 minutes of vigorous-intensity aerobic activity, or an equivalent combination of moderate- and vigorous-intensity activities, so that you can progress as your fitness improves.
  , or an equivalent combination of moderate and vigorous intensity activities, so that you can progress as your physical condition improves.
- Strength training: at least two days a week, you should perform moderate- or higher-intensity muscle-strengthening activities to work all major
  muscle groups.

This approach will be implemented in both the prehabilitation phase and post-surgical rehabilitation, adapting to the patient's needs. However, it should be noted that patients who attend Dr. Gracia's clinic will follow the nutritional guidelines indicated by the Endocrinology and Nutrition Section of the C.M.E. Grande Covián and the Royo Villanova Hospital.

All patients will be evaluated by specialized physical therapists, and the assessment procedure will be as follows:

- T_1: pre-intervention, assessment before starting treatment in the prehabilitation phase.
- T_2 : assessment on the same day of hospital discharge, once the patient has undergone colorectal resection.
- T_3: assessment 30 days after surgery.
- T_4 : follow-up assessment 3 months after surgery, at the end of the study program study.

Data will be collected to evaluate the effectiveness of prehabilitation treatment and post-surgery rehabilitation compared to the conventional program using a guidebook during the period up to 30 days after colorectal cancer surgery. In addition, these patients will be followed up for 3 months after discharge to analyze the percentage of postoperative complications after this rehabilitation treatment.

- **Primary variable:**
  - Postoperative complications: these will be evaluated using the *Comprehensive Complication Index* (CCI). It classifies complication scenarios on a visual analog scale from 0 to 100; a value of 0 reflects the absence of complications, while a CCI of 100 indicates that the patient has died due to the onset of complications. This formula generates an objective value for postoperative morbidity and mortality and includes all complications that the patient may have experienced (Slankamenac et al., 2013).

- **Secondary variables:**
  - Sociodemographic characteristics: sociodemographic information will be collected from participants at
    Through an *ad hoc* questionnaire: age, gender, weight, height, BMI calculation, place of residence, etc. (San Mauro et al., 2013).
  - Body composition: changes in body composition will be assessed using bioelectrical impedance analysis (BIA) with a Tanita BC-601 scale. In addition, waist circumference will also be measured (Holms et al., 2021).
  - Nutritional ultrasound: the nutritional ultrasound technique will examine muscle changes associated with malnutrition and will be applied to the *rectus femoris* muscle, as it is one of the muscles most frequently referenced in terms of strength and functional capacity (Beunza et al., 2021).
  - Lung capacity: this will be assessed by spirometry, using the criteria established by the Spanish Society of Pulmonology (SEPAR).
  - Muscle strength: for upper limbs, it will be measured using the *Hand Grip Test* (Martín et al., 2015) and for lower limbs, the Sit to Stand Test (Bohannon et al., 1995).
  - Functional capacity: this will be assessed using *the Timed Up and Go Test* (Pereiro et al., 2021), *Six Minute Walking Test* (Triguero-Cánovas et al., 2023), modified Borg Scale (Pires et al., 2022), and the International Physical Activity Questionnaire-Short Form (Lee et al., 2021).
  - Psychosocial factors: *Mini-Mental Adjustment to Cancer Scale* questionnaires will be administered to assess cognitive and behavioral responses to cancer (Calderon et al., 2021), *Hospital Anxiety and Depression Scale* questionnaires will be administered to detect possible cases of anxiety and depression (López-Roig et al., 2019), and *The Pittsburgh Sleep Quality Index* questionnaires will be administered to assess sleep quality (Buysse et al., 1998).
  - Quality of life: this will be measured using the self-administered *EuroQol-5D* questionnaire. This is a generic, standardized tool designed to describe and assess health-related quality of life. It consists of a descriptive system with five dimensions: mobility, self-care, activities of daily living, pain, and anxiety/depression. In addition to a visual analog scale (Cabases et al., 2014).
  - Adherence: this will be assessed using the entries in the paper *logbooks* for the conventional group and the entries in the *notes* section of the telerehabilitation platform for the experimental group.
  - Acceptance of treatment: this will be measured using the *Stanford Expectations of Treatment Scale* self-assessment questionnaire, which examines patients' expectations regarding treatment outcomes.
  - Usability of telemedicine in terms of experience in the use of telematic platforms will be measured using the Telehealth Usability Questionnaire (TUQ), translated and adapted into Spanish.
  - Feasibility of the study: this will be calculated using the recruitment rate, the adherence index, and the report of satisfaction with the treatment received at the end of the study. Patient satisfaction will be measured using an *ad hoc* Likert scale questionnaire.

Likewise, in order to determine the economic impact of adding a physiotherapy prehabilitation program through telerehabilitation to the usual clinical practice of healthcare centers, the Royo Villanova Hospital in Zaragoza will collaborate in determining the allocation and assessing the unit costs, which include both direct and indirect costs related to treatment, medication, hospital stay, and patient care. In the economic evaluation, the **Incremental Cost-Effectiveness** Ratio **(**ICER) will be calculated, expressed in **euros/Quality-Adjusted Life Years (**QALYs).

Statistical analysis:

Statistical analysis will be performed using IBM-SPSS Statistics software version 28. The Kolmogorov-Smirnov test will be used to determine the normality of the data. Intra-group variables will be measured using the Student's t-test for related samples and the Mann-Whitney U test for non-parametric data. Analyses will be performed between groups and within intervention groups using a mixed model of analysis of variance (ANOVA) for repeated measures with Bonferroni post hoc pairwise comparisons when a normal distribution is detected. A nonparametric analysis will be performed when a non-normal distribution is assumed, using the Mann-Whitney U test for between-group comparisons and the Friedman test with Tukey's test to highlight within-group differences. Dichotomous variables will be analyzed using the chi-square test. A significance level of 95% ($p \leq 0.05$) will be assumed. Descriptive statistics will also be used to report on the feasibility of recruitment and adherence to the program. Variables will be described as mean and standard deviation (SD) or median and interquartile range. If there are more than 15% dropouts, an intention-to-treat (ITT) analysis will be performed. The effect size will be calculated using Cohen's d to determine clinical significance: insignificant, small, medium, and large differences will be reflected in effect sizes of <0.2, 0.2-0.5, 0.5-0.8, and >0.8, respectively.

Applicability and Usefulness of Results:

The implementation of telerehabilitation can have a significant clinical impact on various aspects of patient care and recovery. The following describes the clinical benefits or impacts that this study may contribute to telerehabilitation:

- ✓ **Benefits in clinical application:** if the positive effect of the treatment in the experimental group is corroborated, the results could be directly applicable in clinical practice to improve the care of patients undergoing oncological processes.
  of patients undergoing cancer treatment.
- ✓ **Wide accessibility:** it could facilitate the coverage of rehabilitation services globally, eliminating geographical barriers and improving the availability of care in areas that are difficult to access.
- ✓ **Reduced waiting times:** it could streamline therapeutic services, minimizing waiting times for patients.
- ✓ **Personal**ization **and adaptation:** if the study shows a positive impact on health, individualized rehabilitation programs tailored to the specific needs of each patient could be implemented.
  patient.
- ✓ **Continuous monitoring:** Through remote monitoring, healthcare professionals could constantly monitor patients' progress to help prevent complications
  post-surgery complications.
- ✓ **Health policy**: if the study shows a positive impact on the health of the population, the results could be applied in the formulation of health policies to promote similar approaches in
  medical care and rehabilitation.
- ✓ **Economic efficiency:** if a positive effect is confirmed, it could contribute to cost reduction by minimizing expenses related to transportation and hospital stays.
- ✓ **Emotional and educational support:** It could incorporate emotional support and therapeutic education services, strengthening the management of stress and anxiety related to the disease.
- ✓ **Benefits for future research** focused on oncological processes.

Limitations of the study:

- Limited scientific evidence on the use of telerehabilitation.
- Follow-up will only be carried out in the experimental group, which may affect adherence in the experimental group.
- This study carries the risk of memory bias in the control group participants, as they may not fill in their diaries every day and may take all their notes at the end, which may be
  incorrect. In the experimental group, this memory bias can be overcome because control messages will be exchanged between the physical therapist and the patient via the web platform, but in the control group it will have to be considered a limitation.
- To avoid bias in the assessment of the intervention, the physical therapist responsible for performing the assessments will not know the assignment of each subject.
- In order to reduce analysis bias in the results, cases that drop out of the study due to problems with the platform, lack of motivation to follow
  the prescribed program, or any other incident, provided that they complete the post-intervention assessment, as the aim is to identify limitations to adherence in order to propose improvements in future randomized clinical trials.

4.7 Ethical aspects (risk/benefit balance, **justification in case of requesting exemption from** informed **consent**, healthcare implications, implications for the participant or their family, compensation for participants, insurance policy).

This study complies with the fundamental principles established in the Declaration of Helsinki, the Council of Europe Convention on Human Rights and Biomedicine, the UNESCO Universal Declaration, and the law on the protection of participants' personal data: EU Regulation 679/2016 on personal data protection (GDPR) and Organic Law 3/2018 of December 5 on Personal Data Protection and Guarantee of Digital Rights (LOPDGDD). All personal data will remain anonymous and confidential.

Informed consent

All patients will sign an informed consent form before participating in the study, i.e., on the first day of the initial assessment. Their participation will be conscious, free, and voluntary. Participants will not receive any financial or other compensation for their participation.

Risks and contingencies

The planned intervention does not pose any health risks to any of the participants, as the exclusion criteria have been adjusted to avoid the participation of patients for whom intensive intervention could be contraindicated. All patients will have the telephone number of the principal investigator (indicated on the participant information sheet) and the referral hospital in case of any incident.
In addition, the experimental group will have a communication channel through the platform.
As registered physical therapists, all researchers have civil liability insurance.

Data protection

The Organic Law on Data Protection will be complied with at all times. Each participant will be assigned an identification code (IC) from the list in order of call, so that their anonymity is guaranteed at all times, and so that no data on the subject is included in the study database and no one except the principal investigator can access the participant's identity.
Data collection will be carried out by a researcher from the team in an encrypted data collection notebook (CRD) with password access. The data collection computer will remain at all times at the researcher's home, where no other people will have access to it. The original paper documents will be collected by the internal project manager, who will keep them in a locked drawer in a secure location. This
person will be the only one with access to the key and the documents. Data analysis will be carried out by a member

of the research team, who will only have access to the DCR with the pseudonymized data once the field phase with the participants' ICs has been completed, without any personal data.

The entire privacy policy of the HEFORA platform can be consulted at www.hefora.net. FISIO CONSULTORES S.L. is responsible for processing the personal data of platform users, and the data is processed in accordance with Regulation (EU) 2016/679 of the European Parliament and of the Council of April 27, 2016, on Data Protection (GDPR). As a user, you have the right to access, rectify, and delete your data, as well as other rights indicated in the additional information available via email. info@hefora.com

In accordance with Regulation (EU) 2016/679 of the European Parliament and of the Council of April 27, 2016, on Data Protection (GDPR) and Organic Law 3/2018, of December 5, on Personal Data Protection and Guarantee of Digital Rights, the participant is informed that the data controller for their personal data will be the UNIVERSITY OF ZARAGOZA.

With regard to the University of Zaragoza:
You may exercise your rights of access, rectification, erasure, and portability of your data, as well as your rights to restrict and object to its processing, in accordance with the provisions of the General Data Protection Regulation (GDPR), by contacting the internal data controller for this project, whose contact details are provided at the top of this document, or by sending an email to the Data Protection Officer at the University of Zaragoza (dpd@unizar.es). If your request is not dealt with, you can lodge a complaint with the Spanish Data Protection Agency (https://www.aepd.es). You can find additional and detailed information on this data processing in the University of Zaragoza's Inventory of Processing Activities, accessible at the following link: Inventory of processing activities | Data Protection Unit (unizar.es).

Participants may withdraw from the study at any time by notifying the principal investigator, although they are informed that their data cannot be deleted in order to guarantee the validity of the research and ensure compliance with the legal obligations of the data controller.

Healthcare implications
The Royo Villanova Hospital in Zaragoza will serve as the reference center where participants will be recruited and assessments will be carried out. The Tele-Rehabilitation program will be carried out at home or in the patient's environment, so it will not interfere with the clinical activities of the center. On assessment days, the evaluating physical therapist will be allowed to devote their time to this task, as indicated on the authorization form.

4.8 Schedule and work plan:

- Stages of development, duration, start and end dates,
- Places where the project is expected to be carried out, facilities that will be used.

Actions	Person responsible	Period of implementation (months)															
		2023	2024												2025		
		D	E	F	M	M	M	J	J	A	S	N	N	D	E	F	M
Final Protocol Drafting	IP+ CCM	X															
Assessment Training	IP+ F	X	X														
Recruitment	IP+ M		X	X	X	X	X	X	X	X	X	X					
Protocol implementation (Treatment)	F			X	X	X	X	X	X	X	X	X	X				
Follow-up	IP				X	X	X	X	X	X	X	X	X	X	X	X	
Database development	IP+ CCM			X	X	X	X	X	X	X	X	X	X	X	X	X	
Quality Control	+ IP CCM	X	X	X	X	X	X	X	X	X	X	X	X	X	X	X	X
Data analysis	IP+ CCM													X	X	X	
Publication of results	All																X

PI = Principal Investigator.
MQC = Methodological Quality Center.
M = Head of Surgery at Royo Villanova Hospital, Dr.
Blas F = Specialist Physical Therapist

PLEASE REMEMBER to submit the documentation, as applicable, along with this form for project evaluation (digital format)**:**

1. Commitment of the principal investigator and collaborators (Annex I)
2. CVs of all researchers (principal investigator and collaborators)
3. Participant Information Sheet and Informed Consent Form (see template on the CEICA website) **or** Request for HIP/CI waiver and authorization for access to records (e.g., medical history).
4. Financial report/source of funding
5. Authorization for the use of resources (Annex II)
6. Insurance policy certificate (if applicable).
7. CE marking certificate and technical data sheet (if it is a medical device).
8. Commitment to use pseudonymized data **ONLY if section 3.3** (use of secondary source data) **is complied with** (available on the CEICA website)
9. Affidavit in the case of observational studies with medicinal products (EOM) without commercial interest, if applicable (see template on the CEICA website)
10. If the study is carried out at the University, the University's authorization for the processing of personal data must be submitted, unless data from the Aragon Health System is used.
11. If the study is conducted in a school, residence, association, sports club, private clinic, or similar facility, authorization from the person in charge of the facility must be submitted in order to conduct the study (free format).

APPENDIX I. COMMITMENT OF THE PRINCIPAL INVESTIGATOR

Mr. José Manuel Burgos Bragado, with Tax ID Number XXXXXXXXD,
From the Service/Department: Department of Physiatry and Nursing. Faculty of Health Sciences
From the Center/Institution: University of Zaragoza.

CERTIFIES

That he is aware of and agrees to participate as Principal Investigator in the study entitled:

Tele-Rehabilitation in cancer patients: optimization of prehabilitation and rehabilitation after colorectal resection. Randomized Clinical Trial.

- It undertakes to ensure that each participant is treated and monitored in accordance with the protocol authorized by the Research Ethics Committee of the Community of Aragon and by the competent authority.
- That it will respect the ethical and legal standards applicable to this type of study.
- That it will guarantee the confidentiality of the data collected, in compliance with LO 3/2018 on the protection of personal data.
- That it will report annually to the CEICA on the progress of the trial, as well as if any modifications are made to the protocol or if it is interrupted.
- That, once the study is completed, it will provide a copy of the final report and any publications derived from it.

That the study will be carried out with the participation of the following collaborating researchers:

First and last names:	Tax ID number:	Signature:
Sandra Calvo Carrión	XXXXXXXXK	
Carolina Jiménez Sánchez	XXXXXXXXZ	
Juan Luis Blas Laina	XXXXXXXXV	
Paula Gracia Gimeno	XXXXXXXXM	
Jorge Alamillo Salas	XXXXXXXXC	
Daniel Fernández Sanchis	XXXXXXXXK	
Beatriz Carpallo Porcar	XXXXXXXXQ	
Natalia Brandín de la Cruz	XXXXXXXXX	

Accepted by: Head of Service/Health Center Coordinator*/Head of Department (University). **First and Last Name: Noelia Herrero Arenas**

Signature:

***** If there are several Health Centers in the same sector, the **Director of Primary Care for the Sector** must sign. If it involves several sectors, the SALUD Manager must sign.

Signed by the Principal Investigator: José Manuel Burgos Bragado, on November 5, 2023.

Any personal data contained in this communication will be incorporated into the processing system for which the Aragon Health Sciences Institute (IACS) is responsible. The data will be processed for the management and monitoring of studies evaluated by CEICA. The data will be deleted once the request has been responded to and/or processed and is no longer necessary. You have the right to access, rectify, and delete the data, as well as other rights granted to you by data protection regulations before the IACS, with address at the Aragon Biomedical Research Center. Avda. San Juan Bosco, nº 13, 500009, Zaragoza, or by requesting it via email at protecciondedatos.iacs@aragon.es.

APPENDIX II. AUTHORIZATION FOR THE USE OF RESOURCES IN RESEARCH

This document must be signed by the head of service or equivalent institutional representative of the center where the study will be conducted.<br>*The purpose of this document is to ensure that the person responsible for the institution where the study is being conducted is aware of*<br>*the research and accepts the use of resources or the expense that it may entail for the institution, so as to ensure the proper and efficient use of resources such as professionals' time, equipment and devices, complementary tests, etc.*

M r / M s ________________________ , as head of the

Service/Center/Department/Institution: ___

I DECLARE THAT

I acknowledge and agree to the study mentioned below being carried out, under the conditions that have been explained to me:
PROJECT TITLE:
PRINCIPAL INVESTIGATOR: Error! Reference source not found.

The study has external funding:

☐ No

☐ Yes. Specify source and attach budget: ___________________________________

The study involves the collaboration of resources, tests, or personnel from other services

☐ No

☐ Yes. Specify resources, tests, or personnel and service: _______________________

In this case, signature of the Head of Service involved: _____

The study involves the use of resources* from the service or institution

☐ No

☐ Yes, paid for by the researcher. Specify resources and approximate total cost:

☐ Yes, paid for by the institution[1]. Specify resources and approximate total cost:

* Include in this section the use of facilities or the performance of tests that involve an expense beyond the normal operation of the institution. In this case, an additional signature must be obtained (see below).

And for the record, I sign

this: on ______________

[1]In this case, also obtain the signature of the medical director of the center or equivalent:
