# Supplementary material for: Correction: Asynchronous telerehabilitation in prehabilitation and postoperative recovery for colorectal cancer: A protocol for a randomized controlled trial
Source: PLoS One. 2026 Jun 3;21(6):e0350693. doi: 10.1371/journal.pone.0350693 (PMC13232795; doi:10.1371/journal.pone.0350693)
Supplement: S4 File — (PDF) [file pone.0350693.s005.pdf]

**TÍTULO**	**Tele-Rehabilitación en pacientes oncológicos: optimización de la prehabilitación y rehabilitación tras una resección colorrectal. Ensayo Clínico Aleatorizado.**
**VERSIÓN Y FECHA**	Versión 3. 13 de noviembre de 2023
**CENTRO DONDE SE REALIZA EL ESTUDIO**	Sección de *Cirugía* General y del Aparato Digestivo en el ***Hospital Royo Villanova* de Zaragoza**

**INVESTIGADOR PRINCIPAL DEL PROYECTO**	
**NOMBRE Y APELLIDOS***	José Manuel Burgos Bragado
**DNI***	XXXXXXXXX
**E-MAIL***	xxxxxxxx@usj.es
**TELÉFONO***	XXX XXX XXX
**PUESTO DE TRABAJO**	Docente Universitario en la Universidad San Jorge.<br>Doctorando en la Universidad de Zaragoza.
**SERVICIO/DEPARTAMENTO**	Grado en Fisioterapia
**CENTRO/ FACULTAD-UNIVERSIDAD/ OTRO**	UNIVERSIDAD SAN JORGE

**PROMOTOR (Imprescindible para ensayos clínicos y estudios observacionales con medicamentos)**	
**IDENTIFICACIÓN EMPRESA/ NOMBRE Y APELLIDOS***	No hay
**NIF/DNI***	
**E-MAIL***	
**TELÉFONO***	
**CONTACTO DE LA PERSONA ENCARGADA DE GESTIONAR LA SOLICITUD**<br>*Si lo desea, añada los datos de contacto de la persona encargada de gestionar los detalles de la solicitud*	
**NOMBRE Y APELLIDOS**	José Manuel Burgos Bragado
**E-MAIL**	xxxxxxxxxxxxxxxx@gmail.com
**TELÉFONO**	XXX XXX XXX

Los datos de carácter personal que pudieran constar en esta comunicación serán incorporados al sistema de tratamiento del que es responsable el Instituto Aragonés de Ciencias de la Salud (IACS). Los datos serán tratados para la gestión y seguimiento de los estudios evaluados por el CEICA. Los datos serán suprimidos cuando se haya dado respuesta a la gestión y/o tramitación de la solicitud y hayan dejado de ser necesarios. Tiene derecho a acceder, rectificar y suprimir los datos, así como los demás derechos que le otorga la normativa de protección de datos ante el IACS, con domicilio en el Centro de Investigación Biomédica de Aragón. Avda. San Juan Bosco, nº 13, 500009, Zaragoza o solicitándolo a través del mail protecciondedatos.iacs@aragon.es.

GLOSARIO

- Anonimización: proceso por el cual deja de ser posible establecer por medios razonables el nexo entre un dato y el sujeto al que se refiere. Es aplicable también a la muestra biológica.
- BIGAN: plataforma de big data sanitario (gestionada por el IACS) que permite acceder a los datos del Sistema Aragonés de Salud de forma seudonimidaza para su uso en gestión e investigación.
- Biobanco: establecimiento público o privado, sin ánimo de lucro, que acoge una o varias colecciones de muestras biológicas de origen humano con fines de investigación biomédica, organizadas como una unidad técnica con criterios de calidad, orden y destino.
- Centro: Institución donde se realiza un estudio (hospital, centro de salud, residencia, facultad, clínica privada colegio, etc). **En caso de duda se debe hacer referencia al lugar de donde proceden los participantes** (hospital, colegio, club deportivo, etc)
- Compromiso de Confidencialidad: Documento que, de forma obligatoria, deben firmar los alumnos y residentes que desarrollan alguna actividad en el sistema público de salud (modelo establecido en la Orden SSI/81/2017).
- Consentimiento informado: manifestación de la voluntad libre y consciente válidamente emitida por una persona capaz, o por su representante autorizado, precedida de la información adecuada.
- Dictamen del CEICA: documento que acredita que el CEICA ha evaluado un proyecto de investigación y que dicho proyecto cumple con las normas legales aplicables y los criterios éticos.
- Encuesta: recogida de información en soporte físico o digital, con o sin interacción directa con el sujeto fuente.
- Entrevista: recogida de información con interacción directa con el sujeto fuente, mediante respuestas verbales.
- Equipo de investigación: Conjunto de investigadores que realizan de forma conjunta un proyecto concreto.
- Fuente primaria de datos: Cuando los datos se recogen directamente del participante en el estudio y con motivo del estudio.
- Fuente secundaria de datos: Cuando se utilizan para el estudio datos ya recogidos (y registrados, por tanto) que se obtuvieron con una finalidad diferente al estudio (asistencial, docente, etc.).
- Grupo de investigación: Conjunto de investigadores con una trayectoria común (publicaciones, financiación) dirigidos o coordinados por un Investigador Principal, agrupados en torno a una temática de investigación y no necesariamente por su vinculación asistencial o departamental.
- Hoja de información al participante: Documento por el que se informa a los potenciales participantes de la naturaleza del estudio, para que puedan otorgar su consentimiento informado.
- Intervención (estudio de intervención): cualquier actuación que se vaya a realizar sobre una persona debido a su participación en un estudio (puede ser un tratamiento farmacológico, fisioterapéutico, una intervención educativa, conductual, psicológica).
- Investigador principal: Investigador que lidera el proyecto y se hace responsable de su diseño, realización y difusión de los resultados. Si el estudio es multicéntrico debe haber un investigador principal en cada centro que se responsabilice de los pacientes, datos y/o muestras.
- Muestra biológica: cualquier material biológico de origen humano susceptible de conservación y que pueda albergar información sobre la dotación genética característica de una persona
- Práctica clínica habitual: Procedimientos que se llevan a cabo con motivos puramente asistenciales, de forma independiente de la participación o no de una persona en un estudio de investigación.
- Proyecto de investigación: procedimiento científico destinado a recabar información y formular hipótesis sobre un determinado fenómeno social o científico.
- Promotor: individuo, empresa, institución u organización responsable de iniciar, gestionar, organizar y financiar un estudio.
- Seudonimización: el tratamiento de datos personales de manera tal que ya no puedan atribuirse a un interesado sin utilizar información adicional, siempre que dicha información adicional figure por separado y esté sujeta a medidas técnicas y organizativas destinadas a garantizar que los datos personales no se atribuyan a una persona física identificada o identificable.

1. ALCANCE Y FINANCIACIÓN DEL PROYECTO	
¿Es un proyecto multicéntrico? Sí ☐ No ☒	En caso afirmativo, se debe presentar el listado de centros completo y rellenar un compromiso del equipo investigador **por cada centro de Aragón** (ver anexo I)
¿Dispone de financiación específica para el estudio? Sí ☐ No ☒	En **todos los casos** se debe rellenar el anexo II: autorización uso de recursos. Además, en caso afirmativo, se debe presentar el presupuesto y la fuente de financiación.

2. CARACTERÍSTICAS DEL ESTUDIO	
**2.1 ¿Se trata de una investigación con medicamentos?** Sí ☐ No ☒	
En caso afirmativo, escoja una opción:<br><br>1) ☐ Se trata de un estudio observacional respecto al tratamiento con medicamentos (EOM)<br>En este caso, especificar:<br>☐ Recogida de datos prospectivos ☐ Recogida de datos retrospectivos ☐ Recogida transversal de datos<br><br>2) ☐ Se trata de un estudio de intervención: ensayo clínico con medicamentos<br>En este caso se debe presentar según instrucciones de la AEMPS (https://www.aemps.gob.es/medicamentos-de-uso-humano/investigacionclinica_medicamentos/ensayosclinicos/#n-espanola)	
**2.2 ¿Se trata de una investigación con productos sanitarios o dispositivos médicos?** Sí ☐ No ☒	
En caso afirmativo, escoja una opción:<br><br>1) ☐ Se trata de un estudio observacional respecto al uso del producto sanitario<br><br>2) ☐ Se trata de un estudio de intervención: ensayo clínico con productos sanitarios<br>En este caso se debe presentar según PNT del CEICA para este tipo de estudios (https://www.iacs.es/investigacion/comite-de-etica-de-la-investigacion-de-aragon-ceica/)	
**2.3 ¿Se trata de una investigación con procedimientos invasivos?**<br>(Definición: toda intervención realizada con fines de investigación que implique un riesgo físico o psíquico para el participante).<br><br>En caso afirmativo se debe contratar una póliza de seguros o justificar riesgo mínimo	Sí ☐ No ☒
**2.4 ¿En la investigación se incluyen menores de edad o personas incapaces de dar su consentimiento?**<br><br>En caso afirmativo se debe presentar un documento de información y consentimiento informado dirigida al tutor/representante legal/familiar y otro dirigido al menor de edad (adaptado a su capacidad). Revisar plantilla CEICA.	Sí ☐ No ☒

2. CARACTERÍSTICAS DEL ESTUDIO	
**2.5 ¿Se utilizan muestras biológicas en el estudio?**	Sí ☐ No ☒
En caso afirmativo, escoja una o varias opciones:<br><br>1) ☐ Se utilizan muestras de excedentes asistenciales con consentimiento para el proyecto<br>Se debe presentar el documento de información y consentimiento (plantilla CEICA)<br><br>2) ☐ Se utilizan muestras de excedentes asistenciales sin consentimiento<br>Se debe justificar adecuadamente en el apartado de aspectos éticos (art. 58.2 Ley 14/2007)<br><br>3) ☐ Se recogen muestras específicamente para este estudio<br>Se debe presentar el documento de información y consentimiento (plantilla CEICA)<br><br>4) ☐ Se crea una colección de muestras privada<br>Para la creación de una nueva colección, presentar la documentación necesaria para su evaluación e indicar nº de registro (nº______________) (ver web CEICA)<br><br>5) ☐ Se utilizan muestras ya recogidas en una colección de muestras privada<br>En este caso, identificar nº de colección y responsable:______________________<br><br>6) ☐ Se solicitan muestras a un Biobanco autorizado. En este caso, identificar el Biobanco:__________<br>Se debe presentar la solicitud al biobanco	
**2.6 ¿Se realizan análisis genéticos?**	Sí ☐ No ☒
**2.7 ¿Se utilizan embriones, células embrionarias humanas, células o tejidos fetales humanos o bien células humanas pluripotentes obtenidas mediante reprogramación celular?**<br>Debe contactar con el IACS o con la institución responsable para posteriores autorizaciones	Sí ☐ No ☒

3. TRATAMIENTO DE DATOS PERSONALES

Se considera **Dato personal** cualquier dato (edad, sexo) o cualquier información (numérica, alfabética, gráfica, acústica) sobre una persona física identificada o identificable; se considerará identificable toda persona cuya identidad pueda determinarse, directa o indirectamente (es decir, que no se haya anonimizado de forma irreversible en origen)

**3.1 ¿Se recogen o tratan datos de carácter personal en el estudio?**	Sí ☒ No ☐

En caso afirmativo, marca la opción aplicable:

☒Se solicita el consentimiento informado del interesado
Presentar el documento de información y consentimiento (plantilla CEICA)

☐Se utilizan datos obtenidos con otra finalidad que han sido seudonimizados (por ej. Historia clínica, otra investigación, otros registros) conforme a la D.A. 17 de la Ley Orgánica 3/2018.

☐Otros. Especificar:

Marque las **categorías** a las que pertenecen los datos recogidos:

☐ **datos identificativos** (Nombre, dirección, email, DNI, nº Historia clínica, teléfono, firma, IP, geolocalización, imagen/voz, otros)
☒ **datos personales:** fecha nacimiento, lugar de nacimiento, nombre padres, lugar de trabajo, datos económicos, sexo, estado civil, hijos, titulación académica, otros.
☐ **datos de opinión**
☐ **datos especialmente sensibles**: salud, etnia, religión, opinión política, vida u orientación sexual, afiliación sindical, necesidades educativas especiales

3.2 Si los datos se recogen directamente del interesado (fuente primaria), especificar el procedimiento (Ejemplo: entrevista, encuesta en papel, correo electrónico, teléfono, aplicaciones web, …)
Entrevista personal con el interesado.

3.3 Si no se recogen directamente del interesado (fuente secundaria), marque la opción y especifique:

☐ Se reutilizan datos de otra investigación similar, para la cual se obtuvo el consentimiento del interesado. Se debe presentar permiso del responsable de los datos, el modelo de consentimiento con el que fueron obtenidos y el compromiso de utilización de los datos seudonimizados (ver en la web).
☐ Se utilizan datos obtenidos con otro fin y sin consentimiento para investigación (por ej, Historia clínica u otro registro)
En este caso, indicar:

- ☐ El investigador (si es personal del centro) accede directamente a la historia clínica. Presentar permiso del responsable de los datos (si se trata de la historia clínica, se debe presentar la autorización de la dirección para el acceso a datos para este estudio)
- ☐ El investigador recibe los datos ya seudonimizados Presentar el compromiso de utilización de los datos seudonimizados (ver en la web).

Nota: **los investigadores que no tengan relación laboral con el hospital/centro** no tienen acceso a la historia clínica, por lo que SIEMPRE que se use esta fuente deben obtener los datos seudonimizados.
En todos los casos, explicar: origen de los datos, responsable de los mismos
Todos los datos serán obtenidos de la entrevista personal con el interesado, y trasladados al CRD de forma seudonimizada. Los CRD serán conservados siguiendo las medidas de protección adecuadas.
☐ Datos procedentes de BIGAN

Presentar informe de la Unidad de Biocomputación (link a la solicitud) y el compromiso de utilización de los datos seudonimizados (ver en la web)

3.4 Una vez obtenida la información y los datos ¿cómo se garantiza la privacidad de los participantes?

☐ Sólo se utilizan datos agregados (es decir, datos que corresponden a grupos de personas y no a cada una de esas personas)

☐ Los datos se anonimizan (los datos no pueden asociarse a una persona identificada o identificable por haberse destruido el nexo irreversiblemente con toda información que identifique al sujeto)

☒ los datos se seudonimizan o codifican (se sustituyen los identificadores directos por un código/seudónimo que sólo conoce el equipo investigador)

Explicar cómo y quién realiza la medida adoptada:
Asignación de un código alfanumérico de identificación. El investigador José Manuel Burgos realizará dicho procedimiento. Todo documento en papel será custodiado en la universidad, en un lugar bajo llave. Los datos seudonimizados estarán organizados en un libro Excel y un archivo SPSS, que contendrán una contraseña para su acceso.

3.5 Plazo de conservación de los datos: especificar fecha de destrucción:

Septiembre de 2027 cuando se espera se hayan publicado todos los datos extraídos de este proyecto.

Generalmente se considera adecuado conservar hasta la publicación, si no, se debe justificar

3.6 Encargados del tratamiento (no cumplimentar en caso de datos anónimos)

Se entiende por **encargado de tratamiento** a toda persona física o jurídica, autoridad pública, servicio u otro organismo que trate datos personales por cuenta del responsable del tratamiento.
Si un tercero (ajeno a la institución) trata datos del proyecto, será necesario firmar un **contrato de encargado de tratamiento**. Un modelo se puede descargar de https://seguridad.salud.aragon.es/plantillas/

Indicar qué personas van a tratar los datos recogidos, especificando quién tendrá acceso a los datos identificativos:
José Manuel Burgos tendrá acceso a todos los datos a través de la entrevista al interesado que recogerá en los CRD, y los cuáles estarán codificados con un código alfanumérico. José Manuel Burgos será el encargado del tratamiento y Sandra Calvo será la responsable del tratamiento desde la Universidad de Zaragoza. El resto de los investigadores solo tendrán acceso a los datos seudonimizados.

¿Todas las personas autorizadas a tratar los datos están sujetas a un acuerdo de confidencialidad firmado con el centro?
Sí ☒ No ☐

3.7 ¿Se van a ceder datos a terceros? Sí ☐ No ☒

En caso de cesión se debe especificar:

- los datos cedidos son: ☐ identificados, ☐ seudonimizados, ☐ anonimizados

- a quién se ceden:

- qué datos se ceden:

- con qué finalidad:

- Explique cómo se seudonimizan o se anonimizan los datos:

Cada usuario/a recibirá un código alfanumérico de identificación en el estudio que no incluirá ningún dato que permita su identificación personal (CRD codificado). El IP (José Manuel Burgos) dispondrá de un listado independiente que permitirá la conexión de los códigos de identificación de los participantes en el estudio con los personales de los mismos. Dicho documento estará archivado en el ordenador portátil corporativo de este investigador con clave de acceso privada.

El IP realizará el volcado de los datos de todos los participantes en el estudio, recogidos en papel de manera seudonimizada en un libro Excel y un archivo SPSS que se pondrán a disposición de los investigadores responsables del manejo estadístico de los datos.

- Si hay transferencias internacionales: especificar empresa y país (en este caso, se debe solicitar el consentimiento expreso del participante para esta cesión)

TRATAMIENTO DE DATOS PERSONALES	
**3.8 ¿Se van a realizar grabaciones (audio/vídeo)?**<br>Recuerde que se necesita consentimiento expreso del interesado para realizar grabaciones, esta información se debe incluir en el documento de consentimiento	Sí ☐ No ☒

En caso de grabación, se debe especificar:

- Dónde se van a conservar, quién tiene acceso y las medidas de seguridad a aplicar:

- Plazo de conservación de las grabaciones:

- con qué finalidad:

- si se utilizan aplicaciones informáticas o almacenamiento en la "nube", se debe indicar quién es el proveedor de servicios y donde está su residencia legal, así como el enlace a su política de privacidad:

3.9 Medidas de seguridad de la información: descripción de los sistemas informáticos que se van a utilizar

Se recuerda que los servidores que contengan datos personales deben estar ubicados en el territorio de la UE (RDL 14/2019)

- Sistema en el que se van a guardar los datos (ordenador personal, servidores corporativos, empresa u organismo externo, proveedor de servicios en la "nube", etc.)

Ordenador corporativo Unizar con nº inventario 258280. Datos seudonimizados en la nube (acceso a web de Google Drive para cuentas Unizar), accesibles mediante los correos corporativos de los/las investigadores/as y sus contraseñas. Dichos datos seudonimizados estarán organizados en un libro excel y un archivo SPSS, que contendrán una contraseña para su acceso.

- Aplicativos que se van a utilizar para el tratamiento de datos (excel, spss, etc.)

Se protegerán mediante contraseña.

- Si se utilizan aplicaciones informáticas online o almacenamiento en la "nube", se debe indicar quién es el proveedor de servicios y donde está su residencia legal, así como el enlace a su política de privacidad.

Proveedor de servicios: Microsoft. https://policies.google.com/privacy. Google Ireland Limited para los usuarios de los servicios de Google que se encuentren en el Espacio Económico Europeo o en Suiza, con domicilio en Gordon House, Barrow Street, Dublín 4, Irlanda.

3.10 Medidas de seguridad de la información: Dispositivos

- Indicar si se va a utilizar algún tipo de dispositivo extraíble (USB portátil, disco duro externo, etc.) y si se van a encriptar

Copia de seguridad en disco duro externo extraíble de José Manuel Burgos, documentos encriptados con zip, mediante carpeta comprimida y contraseña de acceso.

- En el caso de que no se utilicen sistemas informáticos corporativos, indicar si se hacen copias de seguridad.

- Indique las medidas de seguridad para documentos en formato papel (custodia, acceso).

Los documentos en formato papel serán custodiados en la universidad, en lugar bajo llave.

Recomendaciones generales sobre uso de datos

- No utilizar redes Wifi para transmitir información sensible.

- Utilizar contraseñas fuertes y cambiarlas periódicamente.
- Encriptar siempre la información sensible que vaya a enviarse por correo electrónico.
- Procurar que las versiones de los sistemas operativos y los aplicativos estén siempre actualizados.
- En los ordenadores personales, utilizar siempre antivirus y que este actualizado.
- No abrir nunca ficheros adjuntos a correos electrónicos en los que no identifiquemos al remitente.
- No utilizar redes sociales para comunicar información sensible.
- El teléfono móvil es un dispositivo poco seguro para el manejo de información sensible, y los antivirus que se pueden instalar ofrecen poca protección.
- Utilizar siempre que se pueda aplicaciones corporativas
- El uso de USB u otros dispositivos extraíbles está altamente desaconsejado

4. DESCRIPCIÓN DEL PROYECTO DE INVESTIGACIÓN (completar los campos o adjuntar protocolo completo con la información equivalente)

4.1 Tareas del equipo investigador

Explicar brevemente quién participa en el estudio, en calidad de qué y qué tareas va a realizar, así como su filiación (puesto de trabajo actual). Se debe presentar el cv y la firma de todos ellos en el anexo I
Si el estudio es multicéntrico debe presentarse un Anexo I por cada centro

- **José Manuel Burgos Bragado**. Fisioterapeuta. Profesor del Grado en Fisioterapia. Facultad de Ciencias de la Salud. Universidad San Jorge. Grupo de Investigación iPhysio, Universidad San Jorge de Zaragoza. Grupo de Investigación *iHealthy* del Instituto de Investigación Sanitaria de Aragón (IISA25). Doctorando Universidad de Zaragoza.

  Tareas: Investigador Principal. Elaboración de la memoria. Selección de los participantes, y seudonimizacion de éstos. Realización de la intervención del estudio.
- **Sandra Calvo Carrión**. Fisioterapeuta. Profesora del Grado en Fisioterapia. Facultad de Ciencias de la Salud. Universidad de Zaragoza. Grupo de Investigación *iHealthy* del Instituto de Investigación Sanitaria de Aragón (IISA25).

  Tareas: Investigadora. Diseño metodológico. Revisión final de la memoria y futuras publicaciones.
- **Carolina Jiménez Sánchez**. Fisioterapeuta. Profesora del Grado en Fisioterapia. Facultad de Ciencias de la Salud. Universidad San Jorge. Grupo de Investigación iPhysio, Universidad San Jorge de Zaragoza. Grupo de Investigación *iHealthy* del Instituto de Investigación Sanitaria de Aragón (IISA25).

  Tareas: Investigadora. Diseño metodológico. Análisis de resultados. Revisión final de la memoria y futuras publicaciones.
- **Juan Luis Blas Laina.** Jefe del Servicio de Cirugía General y Aparato Digestivo, Hospital Royo Villanova de Zaragoza.

  Tareas: Investigador. Reclutamiento de participantes en el estudio.
- **Paula Gracia Gimeno**. Licenciada en Medicina y Cirugía en la Facultad de Medicina de Zaragoza. Médico del Servicio de Endocrinología y Nutrición, Hospital Royo Villanova de Zaragoza.

  Tareas: Investigadora. Reclutamiento de participantes en el estudio.
- **Jorge Alamillo Salas**. Licenciado en Medicina y Cirugía en la Facultad de Medicina de Zaragoza. Médico del Servicio de Rehabilitación, Hospital Royo Villanova de Zaragoza.

Tareas: Investigador. Reclutamiento de participantes en el estudio.

- **Daniel Fernández Sanchis**. Farmacéutico y Nutricionista. Profesor del Grado en Farmacia. Facultad de Ciencias de la Salud. Universidad San Jorge. Grupo de Investigación iHealthy del Instituto de Investigación Sanitaria de Aragón (IISA25).

  Tareas: Investigador. Realización de los informes de coste efectividad.

- **Beatriz Carpallo Porcar**. Fisioterapeuta. Profesora del Grado en Fisioterapia. Facultad de Ciencias de la Salud. Universidad San Jorge. Grupo de Investigación iPhysio, Universidad San Jorge de Zaragoza. Grupo de Investigación iHealthy del Instituto de Investigación Sanitaria de Aragón (IISA25).

  Tareas: Investigadora. Realización de las valoraciones a los participantes del estudio.

- **Natalia Brandín de la Cruz**. Fisioterapeuta. Profesora del Grado en Fisioterapia. Facultad de Ciencias de la Salud. Universidad San Jorge. Grupo de Investigación iPhysio, Universidad San Jorge de Zaragoza. Grupo de Investigación *iHealthy* del Instituto de Investigación Sanitaria de Aragón (IISA25).

  Tareas: Investigadora. Realización de las valoraciones a los participantes del estudio.

4.2 Justificación del estudio: Antecedentes, estado actual del tema, relevancia (Citar las referencias bibliográficas en el apartado siguiente)

Durante las últimas décadas, los avances en las terapias anticáncer, que incluyen técnicas quirúrgicas, radioterapia y fármacos citotóxicos, han logrado un progreso significativo en términos de aumento de supervivencia y disminución de la recidiva local en el cáncer colorrectal (CCR), evitando la necesidad de una colostomía (1.Araghi et al. 2019). Sin embargo, este vital progreso médico no ha ido acompañado de una mejoría en los resultados funcionales de los pacientes sometidos a una cirugía, los cuales comúnmente experimentan complicaciones postoperatorias (CP) y una notable disminución en su calidad de vida (2.Abarca et al. 2021). Este tipo de tumores malignos conlleva una carga significativa para los sistemas de atención primaria y hospitalaria, y también repercute drásticamente en el ámbito laboral (3.Koopmanschap et al. 2008).

Un CCR es un tumor maligno que aparece cuando un pólipo se desarrolla en el colón o en el recto y representa la segunda neoplasia maligna más prevalente, así como la cuarta causa principal de muerte por cáncer en el mundo, con casi 1,9 millones de casos nuevos y 935.000 muertes en 2020 (4.Gupta et al. 2020). En el año 2017, en España se diagnosticaron un total de 41.441 nuevos casos de CCR y se registraron 14.700 fallecimientos relacionados con esta enfermedad. No obstante, la Sociedad Española de Oncología Médica (SEOM) señala que, de forma general, la supervivencia por cáncer en España ha experimentado un aumento significativo en las últimas décadas gracias a los avances terapéuticos, medidas preventivas y programas de cribado o de detección temprana. Además, se ha observado una clara correlación positiva entre la supervivencia de pacientes con cáncer y la participación en actividades de ejercicio terapéutico, con un énfasis en el trabajo aeróbico, como un factor reductor en cuanto a la mortalidad. Tras una intervención quirúrgica, es común que los pacientes experimenten una disminución de la capacidad funcional debido a la inflamación sistémica y al estrés quirúrgico (5.Carli et al. 2015). Generalmente, la recuperación de los niveles básicos de rendimiento físico puede requerir varios meses. Por consiguiente, la insuficiencia en la capacidad funcional física ha sido identificada como un importante predictor de morbilidad y mortalidad posterior a la cirugía oncológica (6.Pouwels et al. 2015). Es por ello que el proceso de mejorar la capacidad funcional antes de la cirugía, con el fin de optimizar las reservas fisiológicas y afrontar el estrés quirúrgico, ha sido denominado "prehabilitación" (7.Gillis et al.2014).

La prehabilitación es una herramienta de vanguardia en el tratamiento oncológico, diseñada como intervención unimodal o multimodal con el objetivo de reducir complicaciones, la duración de la estancia

hospitalaria y los costes globales, y también mejorar los resultados de la intervención y la calidad de vida de los pacientes. La prehabilitación puede abordar tanto la recuperación física como la mental, así como adaptar un asesoramiento nutricional al paciente (8.Chen et al., 2017). El concepto de prehabilitación se basa en el principio de que el ejercicio estructurado y sostenido durante un período de semanas, conduce a un mejor acondicionamiento cardiovascular, respiratorio y muscular, asociándose con una menor tasa de complicaciones postoperatorias (9.Kulkarni et al., 2010) y a una restauración más temprana de la capacidad funcional (6.Pouwels et al., 2015). Este concepto está fuertemente relacionado con la observación de una mala condición física, así como otros factores modificables como la anemia preoperatoria y el estado nutricional, que constituyen factores de riesgo vinculados a complicaciones postoperatorias graves (10.Bojesen et al., 2022). En el caso del CCR, se encuentran un gran número de complicaciones postoperatorias, con una disminución del 30% al 40% en la capacidad funcional para caminar después de la cirugía en otras. Por lo tanto, la fisioterapia dentro de la fase de prehabilitación en pacientes oncológicos es uno de los pilares principales para mejorar los resultados postoperatorios, mediante programas de ejercicios funcionales y educación terapéutica.

Dentro de este tratamiento de rehabilitación, se ha comprobado que el ejercicio terapéutico para pacientes oncológicos sometidos a cirugía es seguro, aceptable y factible, con mejoras estadísticamente significativas en la capacidad funcional y en la capacidad respiratoria después de la cirugía (11.Awasthi et al., 2019). Diversos estudios han examinado varios factores relevantes en la planificación del ejercicio durante este proceso, en particular, se ha observado que el ejercicio activo y supervisado a nivel físico produce cambios en la capacidad funcional, es decir, acelera el retorno postoperatorio a las actividades iniciales en mayor medida en comparación con el ejercicio no supervisado (12.Gomez et al., 2016).

El tratamiento del CCR en un hospital del sistema de salud público en España tiene un **impacto socioeconómico** considerable, tanto en términos de costos directos como indirectos, los cuales pueden generar una carga económica significativa a largo plazo. En un estudio retrospectivo que evaluó el uso de recursos y los costos médicos directos en una cohorte de 699 pacientes diagnosticados y tratados de CCR durante el período 2000-2006, se observaron variaciones en los costes según la fase en el momento del diagnóstico y la fase de atención asistencial. En la fase de diagnóstico, osciló entre 20708€ (in situ) y 47681€ (estadio III) como promedio a largo plazo. Por otro lado, en cuanto a las fases de tratamiento, el coste promedio del período inicial representó el 24.8% del coste promedio total a largo plazo, mientras que los costes asociados a las fases de atención continua y avanzada representaron el 16.9% y el 58.3%, respectivamente (13.Corral et al., 2016). La población de pacientes está en constante aumento, lo que **podría generar importantes implicaciones económicas y sociales para los sistemas de salud** en el ámbito nacional. La introducción de nuevas estrategias terapéuticas no solo tiene el potencial de mejorar la calidad de vida de los individuos, sino que también representa una interesante oportunidad para la reducción de los costes sanitarios.

El CCR plantea una variedad de opciones de tratamiento, sin embargo, muchas terapias convencionales carecen aún de evidencia sólida en cuanto a su efectividad. Esto añade complejidad y prolonga el proceso de gestión de esta enfermedad. Dadas estas circunstancias, **se destaca la urgente necesidad de desarrollar estrategias adicionales tanto para la prevención como para la prehabilitación, así como para el tratamiento postquirúrgico del cáncer y el asesoramiento nutricional**. La atención actual en el sistema de salud pública para pacientes con CCR a menudo se ve limitada por desafíos relacionados con la eficacia del tratamiento, los tiempos de espera y la calidad del cuidado proporcionado, así como otros relacionados con su viabilidad económica, o con la necesidad de que los pacientes vivan en las proximidades de las instalaciones de atención médica correspondientes (14.Haines et al., 2010).

Por todo ello, **la tele-rehabilitación, que se basa en la prestación de servicios de rehabilitación a distancia mediante el uso de tecnología digital, se presenta como una innovadora alternativa** (15.Russell et al., 2007), ofreciendo soluciones para superar los desafíos relacionados con la distancia, el tiempo y los costes (16.McCue et al., 2010). Esta modalidad tiene el potencial de mejorar significativamente el acceso a la fisioterapia, especialmente para aquellos que residen en áreas rurales, al mismo tiempo que permite una comunicación

rápida y efectiva con los fisioterapeutas. Además, contribuye a la reducción de los costes y a la disminución de las listas de espera en los sistemas de salud a nivel nacional.

Dada la marcada repercusión que el tratamiento oncológico tiene en la calidad de vida de los pacientes, el acceso a **sistemas de tele-rehabilitación** que faciliten la evaluación y el tratamiento de las secuelas derivadas de este proceso, se convierte en una **herramienta de gran utilidad para fomentar la participación activa de los pacientes en su propio cuidado**. Estos sistemas pueden ser empleados para la prevención, la información, el diagnóstico y el seguimiento continuo, proporcionando un *feedback* inmediato sobre la evolución de la condición del paciente. Uno de los desafíos significativos de la rehabilitación convencional es la falta de adherencia por parte de los pacientes, una barrera que la tele-rehabilitación tiene el potencial de superar (17.Sabaté et al., 2003).

Por todo lo anterior, demostrar la eficacia clínica de los programas de tele-rehabilitación en comparación con la rehabilitación convencional es de vital importancia para evaluar su impacto en la calidad de vida de los pacientes oncológicos. Esta estrategia innovadora representa una alternativa para mejorar la calidad de vida de los pacientes oncológicos. Existen estudios que han demostrado la viabilidad y la rentabilidad de estos programas en poblaciones diferentes, como en pacientes diabéticos (18.Kesavadev et., 2012) y en neonatos (19.Isetta et al., 2013).

De esta forma, la implementación de la tele-rehabilitación representaría una mejora significativa en el proceso asistencial del Hospital Royo Villanova del sector 1 del SALUD y, además, serviría como un modelo innovador dentro del sistema público de salud, ya que hasta la fecha no existen proyectos similares en otros centros públicos de Aragón.

4.3 Bibliografía (debe estar referenciada en el texto anterior)

1. Araghi, M., Soerjomataram, I., Jenkins, M., Brierley, J., Morris, E., Bray, F., & Arnold, M. (2019). Global trends in colorectal cancer mortality: projections to the year 2035. *International journal of cancer*, *144*(12), 2992–3000.
2. Abarca, Carmen Paz, & Fernández A., Macarena. (2021). Síndrome de resección anterior baja en pacientes con cáncer de recto medio e inferior ¿Qué más podemos hacer?. *Revista de cirugía*, *73*(1), 80-90
3. Koopmanschap, M. A., van Exel, J. N., van den Berg, B., & Brouwer, W. B. (2008). An overview of methods and applications to value informal care in economic evaluations of healthcare. *PharmacoEconomics*, *26*(4), 269–280.
4. Gupta, S., Coronado, G. D., Argenbright, K., Brenner, A. T., Castañeda, S. F., Dominitz, J. A., Green, B., Issaka, R. B., Levin, T. R., Reuland, D. S., Richardson, L. C., Robertson, D. J., Singal, A. G., & Pignone, M. (2020). Mailed fecal immunochemical test outreach for colorectal cancer screening: Summary of a Centers for Disease Control and Prevention-sponsored Summit. *CA: a cancer journal for clinicians*, *70*(4), 283–298.
5. Carli, F., & Scheede-Bergdahl, C. (2015). Prehabilitation to enhance perioperative care. *Anesthesiology clinics*, *33*(1), 17–33.
6. Pouwels, S., Fiddelaers, J., Teijink, J. A., Woorst, J. F., Siebenga, J., & Smeenk, F. W. (2015). Preoperative exercise therapy in lung surgery patients: A systematic review. *Respiratory medicine*, *109*(12), 1495–1504.
7. Gillis, C., Li, C., Lee, L., Awasthi, R., Augustin, B., Gamsa, A., Liberman, A. S., Stein, B., Charlebois, P., Feldman, L. S., & Carli, F. (2014). Prehabilitation versus rehabilitation: a randomized control trial in patients undergoing colorectal resection for cancer. *Anesthesiology*, *121*(5), 937–947.
8. Chen, B. P., Awasthi, R., Sweet, S. N., Minnella, E. M., Bergdahl, A., Santa Mina, D., Carli, F., & Scheede-Bergdahl, C. (2017). Four-week prehabilitation program is sufficient to modify exercise behaviors and

improve preoperative functional walking capacity in patients with colorectal cancer. *Supportive care in cancer : official journal of the Multinational Association of Supportive Care in Cancer*, *25*(1), 33–40.
9. Kulkarni, S. R., Fletcher, E., McConnell, A. K., Poskitt, K. R., & Whyman, M. R. (2010). Pre-operative inspiratory muscle training preserves postoperative inspiratory muscle strength following major abdominal surgery - a randomised pilot study. *Annals of the Royal College of Surgeons of England*, *92*(8), 700–707.
10. Bojesen, R. D., Grube, C., Buzquurz, F., Miedzianogora, R. E. G., Eriksen, J. R., & Gögenur, I. (2022). Effect of modifying high-risk factors and prehabilitation on the outcomes of colorectal cancer surgery: controlled before and after study. *BJS open*, *6*(3), zrac029.
11. Awasthi, R., Minnella, E. M., Ferreira, V., Ramanakumar, A. V., Scheede-Bergdahl, C., & Carli, F. (2019). Supervised exercise training with multimodal pre-habilitation leads to earlier functional recovery following colorectal cancer resection. *Acta anaesthesiologica Scandinavica*, *63*(4), 461–467.
12. Gomez, I., Szekanecz, É., Szekanecz, Z., & Bender, T. (2016). Daganatos betegek fizioterápiája [Physiotherapy of cancer patients]. *Orvosi hetilap*, *157*(31), 1224–1231.
13. Corral, J., Castells, X., Molins, E., Chiarello, P., Borras, J. M., & Cots, F. (2016). Long-term costs of colorectal cancer treatment in Spain. *BMC health services research*, *16*, 56.
14. Haines, T. P., Sinnamon, P., Wetzig, N. G., Lehman, M., Walpole, E., Pratt, T., & Smith, A. (2010). Multimodal exercise improves quality of life of women being treated for breast cancer, but at what cost? Randomized trial with economic evaluation. *Breast cancer research and treatment*, *124*(1), 163–175.
15. Russell T. G. (2007). Physical rehabilitation using telemedicine. *Journal of telemedicine and telecare*, *13*(5), 217–220.
16. McCue, M., Fairman, A., & Pramuka, M. (2010). Enhancing quality of life through telerehabilitation. *Physical medicine and rehabilitation clinics of North America*, *21*(1), 195–205.
17. Burkhart, P. V., & Sabaté, E. (2003). Adherence to long-term therapies: evidence for action. *Journal of nursing scholarship : an official publication of Sigma Theta Tau International Honor Society of Nursing*, *35*(3), 207.
18. Kesavadev, J., Shankar, A., Pillai, P. B., Krishnan, G., & Jothydev, S. (2012). Cost-effective use of telemedicine and self-monitoring of blood glucose via Diabetes Tele Management System (DTMS) to achieve target glycosylated hemoglobin values without serious symptomatic hypoglycemia in 1,000 subjects with type 2 diabetes mellitus--a retrospective study. *Diabetes technology & therapeutics*, *14*(9), 772–776.
19. Isetta, V., Lopez-Agustina, C., Lopez-Bernal, E., Amat, M., Vila, M., Valls, C., Navajas, D., & Farre, R. (2013). Cost-effectiveness of a new internet-based monitoring tool for neonatal post-discharge home care. *Journal of medical Internet research*, *15*(2), e38.

4.4 Hipótesis (afirmación que se pretende demostrar)

Con relación a la cirugía del CCR y la afectación que tiene sobre la calidad de vida, los efectos de una intervención de fisioterapia con tele-rehabilitación, complementándolo con la atención habitual en el tratamiento convencional, puede reducir las complicaciones postoperatorias y la estancia hospitalaria, permitiendo al paciente conseguir un estado funcional óptimo y, por tanto, una pronta recuperación de su calidad de vida, además, de proporcionar una mejoría asistencial a los pacientes en general.

- **H_0:** La implementación de un programa fisioterapéutico realizado a través de tele-rehabilitación asincrónica, comparado con el grupo convencional, no reduce las complicaciones postoperatorias.
- **H_1:** La implementación de un programa fisioterapéutico realizado a través de tele-rehabilitación asincrónica, comparado con el grupo convencional, reduce las complicaciones postoperatorias.

4.5 Objetivos

Primario:

- Determinar si el programa de tele-rehabilitación asincrónica, con prehabilitación y rehabilitación postquirúrgica, es más efectivo que el tratamiento convencional en pacientes sometidos a cirugía por cáncer colorrectal, para reducir las complicaciones postoperatorias.

Secundarios:

- Determinar si la tele-rehabilitación asincrónica, produce cambios para mejorar la composición corporal.
- Valorar si la tele-rehabilitación asincrónica, mejora los aspectos relacionados con la capacidad cardiorrespiratoria, la fuerza muscular y la capacidad funcional.
- Analizar si la tele-rehabilitación asincrónica, mejora los aspectos psicosociales en los pacientes con procesos oncológicos.
- Identificar si la tele-rehabilitación asincrónica, aumenta la calidad de vida relacionada con la salud de los pacientes.
- Determinar si la tele-rehabilitación asincrónica, aumenta el nivel de adherencia al protocolo multimodal mediante tasas de reclutamiento y de satisfacción.
- Evaluar si la tele-rehabilitación asincrónica, mejora la aceptabilidad respecto al tratamiento efectuado, y la usabilidad de la telemedicina en términos sanitarios.
- Analizar la viabilidad y el coste-efectividad del programa de fisioterapia a través de la Tele-Rehabilitación y determinar el impacto presupuestario de su implantación en el Hospital Royo Villanova.

4.6 Metodología (se deben detallar **todos** los campos siguientes**):**
Diseño del estudio
Participantes: criterios de inclusión/exclusión; modo de reclutamiento (quién y cómo realiza el contacto inicial con los participantes, presentar material de difusión del estudio, si lo hay), tamaño muestral (y su justificación), aleatorización (si procede)
Fuentes de información: variables detalladas (datos a recoger), origen de los datos, cuándo y cómo se recogen, a qué periodo de tiempo se refieren.
Procedimientos: detallar de forma diferenciada los procedimientos puramente asistenciales de los propios de la investigación, presentar encuestas o formularios que se vayan a utilizar (link en caso de ser encuestas online), valoración del riesgo de los procedimientos experimentales y medidas para minimizarlo.
Análisis estadístico
Limitaciones del estudio
En caso de muestras biológicas: detallar tipo y número de muestras, cómo se recogen, dónde y quién las analiza, cuándo se destruyen (o destino final)

Diseño:
El proyecto consistirá en la realización de un Ensayo Cínico Aleatorizado (ECA) simple ciego con cegamiento de los evaluadores.

Participantes:
La estrategia de reclutamiento se realizará en la Sección de Cirugía General y del Aparato Digestivo en el Hospital Royo Villanova de Zaragoza con pacientes diagnosticados de CCR, bajo la supervisión del Dr. BLAS Juan Luis, jefe de cirugía del Hospital Royo Villanova. Se realizará un diseño de grupos paralelos, por lo que cada participante estará expuesto sólo a una intervención, diferenciando un programa convencional a través de un folleto-guía y un programa experimental a través de una plataforma *on-line* de Tele-Rehabilitación.

Entre los participantes reclutados por el Dr. BLAS, un mínimo de pacientes diagnosticados de CCR también serán pacientes que acuden a la consulta de la Dra. GRACIA Paola (médica de la Sección de Endocrinología y Nutrición CME Grande Covián del Hospital Royo Villanova) que trata a los pacientes de riesgo nutricional medio y alto o por tener algún tipo de déficit nutricional. Por tanto, contaremos con pacientes tan solo diagnosticados de CCR y un buen estado nutricional, y pacientes diagnosticados con CCR y con un déficit a nivel nutricional. Por ello, se buscará que los grupos estén balanceados con presencia de estos dos grupos de pacientes y, posteriormente en el análisis estadístico, estos pacientes serán analizados en un análisis secundario.

El cálculo del tamaño muestral es realizado con los datos bibliográficos existentes sobre la escala *Comprehensive Complication Index* (CCI) que cuenta con alto grado de fiabilidad entre evaluadores (Slankamenac et al., 2014). Se han tenido en cuenta los siguientes valores:

- Valor de *alpha*: 0.05.
- Valor de la potencia: 0.8.
- Error beta: 0,2.
- Diferencia mínima de medias: 22.
- Desviación estándar: 18.
- Por posible pérdida de sujetos: se aumenta el total de la muestra en un 30%.

El estudio comprenderá a un total de 80 pacientes, divididos en dos grupos:

- Grupo control o convencional: 40 pacientes.
- Grupo intervención o experimental: 40 pacientes.

Se correlacionarán ambos grupos para conocer la relación significativa entre la intervención con fisioterapia de tele-rehabilitación y la mejoría en los parámetros a evaluar. Se utilizarán además estadísticos descriptivos para informar acerca de la viabilidad del reclutamiento y la adherencia al programa de rehabilitación. El tamaño del efecto será calculado mediante la *d* de *Cohen* para determinar la significación clínica.

Criterios de inclusión:

- Edad entre 18 y 80 años.
- Participantes que comprendan el idioma español.
- Pacientes que se vayan a someter a cirugía programada de CCR en el Hospital Royo Villanova.
- Pacientes que acuden a la primera consulta de la Sección de Cirugía General y del Aparato Digestivo, bajo la supervisión del Dr. Blas, jefe de cirugía del Hospital Royo Villanova de Zaragoza.
- Participantes con independencia funcional que le permita realizar pruebas de marcha y función pulmonar.
- Pacientes con una puntuación en la escala *American Society of Anesthesiologists* (ASA) de valoración preoperatoria de I, II o III.
- Participantes que están de acuerdo en participar y firmen el consentimiento informado.

Criterios de exclusión:

- Pacientes mayores de 80 años.
- Pacientes con una puntuación en la escala *ASA* de valoración preoperatoria de IV.
- Pacientes que padezcan alguna lesión o patología o procesos inflamatorios que imposibiliten la práctica de ejercicio.
- Pacientes con enfermedades neurológicas a nivel central y/o periférico que les impidan seguir el programa de rehabilitación.
- Pacientes con comorbilidades cardíacas inestables, como arritmias, alta presión arterial, angina de pecho u otras patologías que tengan contraindicado el entrenamiento de intensidad moderada.
- Pacientes diagnosticados con enfermedad psiquiátrica, emitido por un psiquiatra.
- Pacientes sin acceso a internet móvil u ordenador con internet en el domicilio.
- Pacientes que obtengan una puntuación ≤ 24 en el *Mini-Examen Cognoscitivo* (MEC).
- Personas que no sean capaces de seguir las instrucciones orales y escritas en español.
- Negativa del paciente a participar en el estudio o que no hayan firmado los consentimientos informados.

Criterios de abandono:

- Decisión propia del paciente de retirarse del estudio.
- Falta de compromiso al tratamiento (tasa de participación < 80%).

Aleatorización:
Una vez el/la paciente cumpla con los criterios de inclusión especificados anteriormente se procederá a la aleatorización utilizando un programa informático y se realizará por bloque de edad y género.
Los participantes firmarán su consentimiento informado por escrito y serán asignados de forma aleatoria al grupo control o al grupo intervención mediante sobres cerrados y preparados en la Universidad de Zaragoza, repartiéndose por orden cronológico de captación en la consulta del Dr. BLAS Juan Luis.

Se recopilarán datos que permitirán evaluar la efectividad del tratamiento de prehabilitación y la rehabilitación postcirugía en comparación con el programa convencional durante el período de hasta 30 días posteriores a la cirugía del cáncer colorrectal. Además, se llevará a cabo un seguimiento de estos pacientes durante 3 meses posteriores al alta para analizar el porcentaje de complicaciones postoperatorias tras este tratamiento de rehabilitación.

Fuentes de información:
¿Cómo se ejecutará el estudio?
Se citará a los pacientes diagnosticados de CCR y se les pasará la escala *American Society of Anesthesiologists* (ASA) de valoración preoperatoria. Todos los pacientes que cumplan los criterios de inclusión serán aleatorizados y ubicados en dos grupos paralelos, por lo que cada participante estará expuesto sólo a una intervención:

- Grupo control: recibirán un programa convencional a través de un folleto-guía con pautas en educación terapéutica, ejercicios respiratorios, ejercicios aérobicos y entrenamiento terapéutico de fuerza.
- Grupo intervención: recibirán un programa experimental que consistirá en el mismo tratamiento que

el grupo control, en cambio se realizará a través de una plataforma digital de tele-rehabilitación. El fisioterapeuta instalará junto con el paciente la aplicación de tele-rehabilitación, este comprobará que funciona correctamente y detallará al paciente como utilizarla (la plataforma no recabará ningún dato personal, el paciente se instalará la plataforma a través de su código identificatorio, y tendrá acceso a todo el programa de rehabilitación, pero en ningún momento deberá grabar ningún dato personal). La sesión de educación terapéutica y los diferentes vídeos del programa de ejercicios serán por vía asincrónica.

Todos los participantes serán informados del tratamiento fisioterapéutico que se les aplicará, y podrán realizar, en cualquier momento del estudio, las preguntas necesarias. Los pacientes del grupo intervención podrán enviar mensajes de control a través de la plataforma web para intercambiar mensajes entre fisioterapeuta-paciente y solucionar posibles problemas con la realización de los ejercicios propuestos, valorar el nivel de fatiga durante la realización del programa y analizar posibles incidencias. Por otro lado, los pacientes del grupo control tendrán marcado en el folleto cómo evaluar su nivel de fatiga y cómo poder progresar con los ejercicios, además de un número de teléfono de uno de los investigadores para llamar si les surgiese algún contratiempo, duda o necesitasen cualquier aspecto.

El programa de rehabilitación que será igual en ambos grupos de pacientes, con la única salvedad del canal de realización (tele-rehabilitación asincrónica o folleto) y de comunicación (mensajes en la plataforma o teléfono del investigador), se llevará a cabo durante un período de seis semanas (dos semanas de prehabilitación y cuatro semanas postquirúrgicas), con sesiones programadas de cuatro días a la semana. El programa se compondrá como se ha dicho anteriormente para ambos grupos de:

- Educación terapéutica en hábitos saludables para la salud, facilitando la realización temprana de ejercicios de movimiento activo y la deambulación para prevenir complicaciones como las atelectasias y la trombosis venosa profunda (TPV). Además, incluye educación sanitaria al paciente respecto al cuidado de las heridas y a otros aspectos de autocuidado que va a necesitar cuando regrese a casa.
- Ejercicios de fisioterapia respiratoria, se realizarán de forma lenta y relajada, en una posición cómoda y en un ambiente confortable. Las actividades a realizar incluyen ejercicios de respiración abdomino-diafragmática y expansión costal con respiraciones dirigidas.

Por otro lado, el programa de ejercicios dinámicos incluye tres niveles de intensidad diferentes, cada uno compuesto entre tres y seis ejercicios, siguiendo las directrices de la OMS sobre Actividad Física y Comportamientos Sedentarios:

- Ejercicios aeróbicos, acumulando a lo largo de la semana un mínimo de entre 150 y 300 minutos de actividad aeróbica de intensidad moderada, o bien un mínimo de entre 75 y 150 minutos de actividad aeróbica de intensidad vigorosa, o bien una combinación equivalente de actividades de intensidad moderada y vigorosa, para que pueda ir avanzando conforme vayan mejorando su condición física.
- Entrenamiento de fuerza, como mínimo dos días a la semana deberán realizar actividades de fortalecimiento muscular de intensidad moderada o más elevada para trabajar todos los grandes grupos musculares.

Este enfoque se implementará tanto en la fase de prehabilitación como en la rehabilitación postquirúrgica, adaptándose a las necesidades del paciente. No obstante, cabe mencionar que los pacientes que acuden a la consulta de la Dra. Gracia, seguirán las pautas nutricionales indicadas por la Sección de Endocrinología y Nutrición del C.M.E. Grande Covián y del Hospital Royo Villanova.

Todos los pacientes serán evaluados por fisioterapeutas especializados y el procedimiento de valoración será el siguiente:

- T_1: pre-intervención, valoración antes de empezar el tratamiento en la fase de prehabilitación.
- T_2: valoración el mismo día del alta hospitalaria, una vez que el paciente se haya sometido a la resección colorrectal.
- T_3: valoración a los 30 días de postoperatorio.
- T_4: valoración de seguimiento a los 3 meses de la intervención quirúrgica, finalizando el programa de estudio.

Se recopilarán datos que permitirán evaluar la efectividad del tratamiento de prehabilitación y la rehabilitación postcirugía en comparación con el programa convencional a través de un folleto-guía durante el período de hasta 30 días posteriores a la cirugía del cáncer colorrectal. Además, se llevará a cabo un seguimiento de estos pacientes durante 3 meses posteriores al alta para analizar el porcentaje de complicaciones postoperatorias tras este tratamiento de rehabilitación.

- **Variable principal:**
  - Complicaciones postoperatorias: serán evaluadas a través de la *Comprehensive Complication Index* (CCI). Clasifica los escenarios de complicaciones en una escala analógica visual de 0 a 100; un valor de 0 refleja la ausencia de complicaciones, mientras que un CCI de 100 indica que el paciente ha fallecido debido a la aparición de las complicaciones. Esta fórmula que genera un valor objetivable sobre la morbimortalidad postoperatoria e incluye todas las complicaciones que haya podido presentar el paciente (Slankamenac et al., 2013).
- **Variables secundarias:**
  - Características sociodemográficas: se recogerá información sociodemográfica de los participantes a través de un cuestionario *ad hoc* : edad, género, peso, talla, cálculo del IMC, lugar de residencia, …etc. (San Mauro et al., 2013).
  - Composición corporal: los cambios de composición corporal se evaluarán mediante el análisis de impedancia bioeléctrica (BIA) con una báscula Tanita BC-601, Además, también se medirá el perímetro de la cintura (Holms et al., 2021).
  - Ecografía nutricional: la técnica de ecografía nutricional examinará los cambios musculares asociados a la desnutrición y se aplicará en el músculo *recto anterior del cuádriceps* debido a ser uno de los músculos más referenciados por la implicación de fuerza y la capacidad funcional (Beunza et al., 2021).
  - Capacidad pulmonar: será evaluada por medio de una espirometría, utilizando los criterios establecidos por la Sociedad Española de Neumología (SEPAR).
  - Fuerza muscular: para miembros superiores se medirá a través del *Hand Grip Test* (Martín et al., 2015) y para miembros inferiores el Sit to Stand Test (Bohannon et al., 1995).
  - Capacidad funcional: se evaluará mediante *Timed Up and Go Test* (Pereiro et al., 2021), *Six Minute Walking Test* (Triguero-Cánovas et al., 2023), Escala de Borg modificada (Pires et al., 2022) y el International Physical Activity Questionnaire-Short Form (Lee et al., 2021).
  - Factores psicosociales: se pasarán los cuestionarios de *Mini-Mental Adjustment to Cancer Scale* para evaluar las respuestas cognitivas y comportamentales del cáncer (Calderon et al., 2021), *Hospital Anxiety and Depression Scale* para detectar posibles casos de ansiedad y depresión (López-Roig et al., 2019) y *The Pittsburgs Sleep Quality Index* para evaluar la calidad del sueño (Buysse et al., 1998).
  - Calidad de vida: se medirá a través del cuestionario autoadministrado *EuroQol-5D*. Es una herramienta genérica y estandarizada, elaborada para describir y valorar la calidad de vida relacionada con la salud. Consta de un sistema descriptivo con cinco dimensiones: movilidad, autocuidado, actividades de la vida diaria, dolor y ansiedad/depresión. Además de una escala visual analógica (Cabases et al., 2014).
  - Adherencia: se evaluará con las anotaciones en los *diarios de registro* en papel para el grupo convencional, y con el registro del apartado de *anotaciones* dentro de la plataforma de tele-rehabilitación para el grupo experimental.
  - Aceptación del tratamiento: se medirá a través del cuestionario de auto-evaluación *Stanford Expectations of Treatment Scale* que examina las expectativas de los pacientes en relación a los resultados del tratamiento.
  - Usabilidad de la telemedicina en términos de experiencia en el uso de plataformas telemáticas que se medirá con el cuestionario Telehealth Usability Questionnaire (TUQ), traducido y adaptado al idioma español.
  - Viabilidad del estudio: se calculará mediante la tasa de reclutamiento, el índice de adherencia, así como el reporte de satisfacción con el tratamiento recibido al concluir el estudio. La satisfacción del paciente se medirá con un cuestionario *ad hoc* tipo escala Likert.

Así mismo, para determinar el impacto económico de la adición de un programa de prehabilitación fisioterápica a través de la tele-rehabilitación a la práctica clínica habitual de los centros sanitarios se contará con la colaboración del Hospital Royo Villanova de Zaragoza para determinar la asignación y valorar los costes unitarios, que incluyen tanto los costes directos como los indirectos relacionados con el tratamiento, la medicación, la estancia hospitalaria y la atención al paciente. En la evaluación económica, se calculará la relación **Coste-Efectividad Incremental** (RCEI) expresada en euros/**Años de Vida Ajustados por Calidad** (AVAC).

Análisis estadístico:
El análisis estadístico se realizará con el software IBM-SPSS Statistics versión 28. La prueba de Kolmogorov-Smirnov será utilizada para determinar la normalidad de los datos. Las variables intra-grupo se medirán con el test T-Student para muestras relacionadas y U-Mann Whitney para las no paramétricas. Se realizarán análisis entre grupos y dentro de los grupos de intervención utilizando un modelo mixto de análisis de varianza (ANOVA) para medidas repetidas con comparaciones post hoc por pares de Bonferroni cuando se detecte una distribución normal. Se realizará un análisis no paramétrico cuando se asuma una distribución no normal, utilizando la prueba U de Mann-Whitney para las comparaciones entre grupos y la prueba de Friedman con la prueba de Tukey para destacar las diferencias dentro del grupo. Las variables dicotómicas se analizarán mediante la prueba de chi-cuadrado. Se asumirá un nivel de significación del 95% ($p \leq 0{,}05$). Se utilizarán además estadísticos descriptivos para informar acerca de la viabilidad del reclutamiento y adherencia al programa. Las variables serán descritas en media y desviación típica (SD) o mediana y rango intercuartílico. Si hay más de un 15% de abandonos se hará un análisis por intención de tratar (ITT). El tamaño del efecto será calculado mediante la d de Cohen para determinar la significación clínica: las diferencias insignificantes, pequeñas, medianas y grandes se reflejarán en tamaños de efecto de <0,2, 0,2-0,5, 0,5-0,8 y >0,8, respectivamente.

Aplicabilidad y Utilidad de los Resultados:
La implementación de la tele-rehabilitación puede generar un impacto clínico significativo en varios aspectos de la atención médica y la recuperación de los pacientes. A continuación, se describen los beneficios o impactos clínicos que este estudio puede aportar con la actuación de la tele-rehabilitación:

- ✓ **Beneficios en la aplicación clínica:** si se corrobora el efecto positivo del tratamiento en el grupo experimental, los resultados podrían ser aplicables directamente en la práctica clínica para mejorar la atención de pacientes en procesos oncológicos.
- ✓ **Amplia accesibilidad:** podría facilitar la cobertura de servicios de rehabilitación a nivel global, eliminando barreras geográficas y mejorando la disponibilidad de atención en áreas de difícil acceso.
- ✓ **Reducción de tiempos de espera:** podría agilizar los servicios terapéuticos, minimizando los tiempos de espera para los pacientes.
- ✓ **Personalización y adaptación:** si el estudio muestra un impacto positivo en la salud, se podría implementar programas de rehabilitación individualizados y ajustados a las necesidades específicas de cada paciente.
- ✓ **Seguimiento continuo:** a través de la monitorización remota, los profesionales de la salud podrían realizar un seguimiento constante de la evolución de los pacientes, para ayudar a prevenir complicaciones posteriores a la cirugía.
- ✓ **Política de Salud**: si el estudio muestra un impacto positivo en la salud de la población, los resultados podrían ser aplicables en la formulación de políticas de salud para impulsar enfoques similares en la atención médica y la rehabilitación.
- ✓ **Eficiencia económica:** si se corrobora un efecto positivo, puede contribuir a la reducción de costes al minimizar los gastos relacionados con el transporte y la estancia hospitalaria.
- ✓ **Soporte emocional y educativos:** podría incorporar servicios de apoyo emocional y educación terapéutica, fortaleciendo la gestión del estrés y la ansiedad relacionados con la enfermedad.
- ✓ **Beneficios para futuras investigaciones** centradas en procesos oncológicos.

Limitaciones del estudio:

- Escasa evidencia científica sobre el uso de la tele-rehabilitación.
- El seguimiento se realizará en tan solo en el grupo experimental, esto puede afectar al incremento de la adherencia en el grupo experimental.
- El presente estudio cuenta con el riesgo del sesgo de memoria en los participantes del grupo control, porque podrían no rellanar los diarios en cada jornada y tomar todas las notas al final, pudiendo ser incorrectas. En el grupo experimental este sesgo de memoria podrá ser suplido ya que se intercambiarán mensajes de control a través de la plataforma web entre el fisioterapeuta y el paciente, pero en el grupo control habrá de contarse como una limitación.
- Para evitar un sesgo en la valoración de la intervención el fisioterapeuta encargado de realizar las valoraciones no conocerá la asignación de cada sujeto.
- Con el objetivo de disminuir el sesgo de análisis en los resultados no serán repuestos aquellos casos que abandonen el estudio por problemas con la plataforma, falta de motivación a la hora de realizar el programa prescrito o cualquier otra incidencia, siempre y cuando realicen la valoración post-intervención, ya que lo que se pretende es conocer las limitaciones para la adherencia con el objetivo de proponer mejoras en futuros ensayos clínicos aleatorizados.

4.7 Aspectos éticos (balance riesgo/beneficio, **justificación en caso de solicitar exención del consentimiento** informado, implicaciones asistenciales, implicaciones para el participante o su familia, compensación a los participantes, póliza de seguro).

Este estudio respeta los principios fundamentales establecidos en la Declaración de Helsinki, en el Convenio del Consejo de Europa relativo a derechos humanos y biomedicina, en la Declaración Universal de la UNESCO y la ley de protección de los datos personales de los participantes: Reglamento UE 679/2016 de protección de datos personales RGPD y Ley Orgánica 3/2018 de 5 de diciembre de Protección de Datos Personales y garantía de los derechos digitales LOPDGDD). Se mantendrá el anonimato y confidencialidad de todos los datos personales.

Consentimiento informado

Todos los pacientes firmarán un consentimiento informado antes de participar en el estudio, es decir, el primer día que se les haga la valoración inicial. Su participación será consciente, libre y voluntaria. Las participantes no recibirán ninguna compensación económica ni de ninguna otra índole por su participación.

Riesgos y contingencias

La intervención prevista no presenta riesgos para la salud de ninguno de los participantes, ya que se han tratado de ajustar los criterios de exclusión y evitar así la participación de pacientes en los cuáles una intervención intensiva pudiese estar contraindicada. Todos los pacientes dispondrán del teléfono del investigador principal (indicado en la hoja de información al participante), y del centro hospitalario de referencia ante cualquier incidencia.
Además, el grupo experimental, contará con un canal de comunicación a través de la plataforma.
Como fisioterapeutas colegiados, todos los investigadores cuentan con un seguro de responsabilidad civil.

Protección de datos

Se cumplirá en todo momento la Ley Orgánica de Protección de Datos. A cada participante se le asignará un código identificativo (CI) de la lista por orden de llamada, de manera que se garantice su anonimato en todo, y de forma que no se incluyan datos del sujeto en la base de datos del estudio y nadie salvo el investigador principal pueda acceder a la identidad del participante.
La recogida de datos la realizará un investigador del equipo en un cuaderno de recogida de datos (CRD) encriptado y con contraseña de acceso. El ordenador de recogida de datos permanecerá siempre en el domicilio del investigador en lugar sin acceso de otras personas. Los documentos originales en papel los recogerá la encargada interna del proyecto, quien los guardará en un cajón con llave en un lugar seguro. Esta persona será la única con acceso a la llave y a los documentos. El análisis de datos los realizará un miembro

del equipo investigador, quien sólo tendrá acceso al CRD con los datos seudonimizados una vez terminada la fase de campo con los CI de los participantes, sin ningún dato personal.

Toda la política de privacidad de la plataforma HEFORA se puede consultar en www.hefora.net. FISIO CONSULTORES S.L. es el responsable del tratamiento de los datos personales de los usuarios de la plataforma siendo los datos tratados de acuerdo con el Reglamento (UE) 2016/679 del Parlamento Europeo y del Consejo de 27 de abril de 2016 de Protección de Datos (RGPD). Como usuario tiene derecho a acceder, rectificar y suprimir los datos, así como otros derechos indicados en la información adicional a través de dirección de correo electrónico info@hefora.com

De acuerdo con el Reglamento (UE) 2016/679 del Parlamento Europeo y del Consejo de 27 de abril de 2016 de Protección de Datos (RGPD) y la Ley Orgánica 3/2018, de 5 de diciembre, de Protección de Datos Personales y garantía de los derechos digitales, el participante queda informado de que el responsable del tratamiento de sus datos personales será UNIVERSIDAD DE ZARAGOZA.

Con respecto a la Universidad de Zaragoza:
Podrá ejercer sus derechos de acceso, rectificación, supresión y portabilidad de sus datos, de limitación y oposición a su tratamiento, de conformidad con lo dispuesto en el Reglamento General de Protección de Datos (RGPD) ante el responsable interno de este proyecto, cuyos datos de contacto figuran en el encabezamiento de este documento, o dirigiendo un correo electrónico al Delegado/a de Protección de Datos de la Universidad de Zaragoza (dpd@unizar.es). Si no viera atendida su petición podrá dirigirse en reclamación a la Agencia Española de Protección de Datos (https://www.aepd.es). Podrá consultar información adicional y detallada de este tratamiento de datos en el Inventario de Actividades de Tratamiento de la Universidad de Zaragoza, accesible en el siguiente enlace: Inventario de actividades de tratamiento | Unidad de Protección de Datos (unizar.es).

El participante podrá retirarse del estudio en cualquier momento comunicándoselo al investigador principal, si bien queda informado de que sus datos no podrán ser eliminados para garantizar la validez de la investigación y garantizar el cumplimiento de los deberes legales del responsable.

Implicaciones asistenciales
El centro hospitalario Royo Villanova de Zaragoza servirá de centro de referencia de donde se reclutarán los participantes, y se realizarán las valoraciones. El programa de Tele-Rehabilitación se realizará en el ámbito domiciliario o en el contexto del paciente por lo que no interfiere en el desarrollo clínico del centro. Para los días de valoración el fisioterapeuta evaluador tendrá permiso para la dedicación a este fin, como así lo indica la hoja de autorización.

4.8 Cronograma y plan de trabajo:

- Etapas de desarrollo, duración, fechas de inicio y fin,
- Lugares donde se prevé realizar el proyecto, instalaciones que se utilizarán.

Acciones	Persona responsable	Periodo de Realización (meses)															
		2023	2024												2025		
		D	E	F	M	A	M	J	J	A	S	O	N	D	E	F	M
Redacción Protocolo Final	IP + CCM	X															
Entrenamiento de Valoración	IP + F	X	X														
Reclutamiento	IP + M		X	X	X	X	X	X	X	X	X	X					
Ejecución del protocolo (Tratamiento)	F			X	X	X	X	X	X	X	X	X	X				
Seguimiento	IP				X	X	X	X	X	X	X	X	X	X	X	X	
Elaboración Base de datos	IP + CCM			X	X	X	X	X	X	X	X	X	X	X	X	X	
Control de Calidad	IP + CCM	X	X	X	X	X	X	X	X	X	X	X	X	X	X	X	X
Análisis de datos	IP + CCM													X	X	X	
Publicación de los resultados	Todos																X

IP= Investigador Principal.
CCM= Centro de Calidad Metodológica.
M= Jefe de cirugía del Hospital Royo Villanova, Dr. Blas
F= Fisioterapeuta especialista

LE RECORDAMOS la documentación a presentar, según aplique, junto con este formulario para la evaluación del proyecto (formato digital)**:**

1. Compromiso del investigador principal y colaboradores (Anexo I)
2. CV de todos los investigadores (principal y colaboradores)
3. Hoja de Información al Participante y Consentimiento Informado (Ver plantilla en la web del CEICA) **o bien** Solicitud de dispensa de HIP/CI y autorización para acceso a los registros (por ej. historia clínica).
4. Memoria económica/ fuente de financiación
5. Autorización para el uso de recursos (Anexo II)
6. Certificado de póliza de seguro (si procede).
7. Certificado marcaje CE y ficha técnica (si es un producto sanitario).
8. Compromiso de uso de datos seudonimizados **SÓLO si cumple el apartado 3.3** (uso de datos de fuente secundaria) (disponible en la web del CEICA)
9. Declaración responsable en caso de estudios observacionales con medicamentos (EOM) sin interés comercial, si procede (ver modelo en la web del CEICA)
10. Si el estudio se realiza en la Universidad se debe presentar la autorización de la Universidad para el tratamiento de datos personales, salvo que se utilicen datos del Sistema Aragonés de Salud.
11. Si el estudio se realiza en algún centro escolar, residencia, asociación, club deportivo, clínica privada o similar, se debe presentar la autorización del responsable del centro para la realización del estudio (formato libre).

ANEXO I. COMPROMISO DEL INVESTIGADOR PRINCIPAL

Don José Manuel Burgos Bragado, con NIF XXXXXXXXD,
Del Servicio/Departamento: Departamento de Fisiatría y Enfermería. Facultad Ciencias de la Salud
Del Centro/Institución: Universidad de Zaragoza.

CERTIFICA

Que conoce y acepta participar como Investigador Principal en el estudio titulado:

Tele-Rehabilitación en pacientes oncológicos: optimización de la prehabilitación y rehabilitación tras una resección colorrectal. Ensayo Clínico Aleatorizado.

- Que se compromete a que cada participante sea tratado y controlado siguiendo lo establecido en el protocolo autorizado por el Comité de Ética de la Investigación de la Comunidad de Aragón y por la autoridad competente.
- Que respetará las normas éticas y legales aplicables a este tipo de estudios.
- Que garantizará la confidencialidad de los datos recogidos, cumpliendo con la LO 3/2018, de protección de datos de carácter personal.
- Que informará anualmente al CEICA de la marcha del ensayo, así como si se introducen modificaciones en el protocolo o se interrumpe.
- Que, una vez terminado el estudio, aportará copia de informe final y de las publicaciones derivadas.

Que dicho estudio se realizará contando con la participación de los siguientes investigadores colaboradores:

Nombre y apellidos:	NIF:	Firma:
Sandra Calvo Carrión	XXXXXXXXK	
Carolina Jiménez Sánchez	XXXXXXXXZ	
Juan Luis Blas Laina	XXXXXXXXV	
Paula Gracia Gimeno	XXXXXXXXM	
Jorge Alamillo Salas	XXXXXXXXC	
Daniel Fernández Sanchis	XXXXXXXXK	
Beatriz Carpallo Porcar	XXXXXXXXQ	
Natalia Brandín de la Cruz	XXXXXXXXX	

Siendo aceptada por: el Jefe de Servicio/ Coordinador del Centro de Salud*/ Jefe de Departamento (Universidad). **Nombre y Apellidos:** **Noelia Herrero Arenas**

Firma:

***** si se trata de varios Centros de Salud de un mismo sector, debe firmar el **Director de AP del Sector**. Si implica a varios sectores, debe firmar el Gerente del SALUD

Firmado por el Investigador Principal: José Manuel Burgos Bragado, a fecha de 05 de noviembre de 2023.

Los datos de carácter personal que pudieran constar en esta comunicación serán incorporados al sistema de tratamiento del que es responsable el Instituto Aragonés de Ciencias de la Salud (IACS). Los datos serán tratados para la gestión y seguimiento de los estudios evaluados por el CEICA. Los datos serán suprimidos cuando se haya dado respuesta a la gestión y/o tramitación de la solicitud y hayan dejado de ser necesarios. Tiene derecho a acceder, rectificar y suprimir los datos, así como los demás derechos que le otorga la normativa de protección de datos ante el IACS, con domicilio en el Centro de Investigación Biomédica de Aragón. Avda. San Juan Bosco, nº 13, 500009, Zaragoza o solicitándolo a través del mail protecciondedatos.iacs@aragon.es.

ANEXO II. AUTORIZACIÓN USO DE RECURSOS EN INVESTIGACIÓN

***Este documento debe ser firmado por el jefe de servicio o responsable institucional equivalente del centro donde se va a realizar el estudio*.**
El fin de este documento es garantizar que el responsable de la institución donde se realiza el estudio conoce la investigación y acepta el uso de recursos o el gasto que pueda suponer a la institución, de forma que se garantice un uso adecuado y eficiente de los recursos tales como tiempo de los profesionales, aparataje y dispositivos, pruebas complementarias, etc.

D/Dña ______________________________ , como responsable del

Servicio/Centro/Departamento/Institución: _____________

DECLARO QUE

conozco y acepto que se realice el estudio abajo mencionado, en las condiciones que me han sido expuestas:
TÍTULO DEL PROYECTO:
INVESTIGADOR PRINCIPAL: ¡Error! No se encuentra el origen de la referencia.

El estudio tiene financiación externa:
☐ No

☐ Si. Especificar fuente y adjuntar presupuesto: _____________________________

El estudio supone la colaboración de recursos, pruebas o personal de otros servicios

☐ No

☐ Si. Especificar recursos, pruebas o personal y servicio: ________________________

En este caso, firma del Jefe de Servicio implicado: _________

El estudio supone el uso de recursos* del servicio o la institución

☐ No

☐ Si, a cargo del investigador. Especificar recursos y coste total aproximado:

☐ Si, a cargo de la institución[1]. Especificar recursos y coste total aproximado:

* Incluir en este apartado el uso de instalaciones o la realización de pruebas que supongan un gasto más allá del funcionamiento habitual de la institución. En este caso, se debe recabar una firma adicional (ver abajo).

Y para que conste lo firmo:

a fecha de ____________

[1]En este caso, recoger además la firma del director médico del centro o equivalente:
